# Immuno-targeting the ectopic phosphorylation sites of PDGFRA generated by MAN2A1-FER fusion in HCC

**DOI:** 10.1097/HC9.0000000000000511

**Published:** 2024-07-31

**Authors:** Muhamuda Kader, Yan-Ping Yu, Silvia Liu, Jian-Hua Luo

**Affiliations:** 1 Department of Pathology, University of Pittsburgh School of Medicine, Pittsburgh, Pennsylvania, USA; 2 High Throughput Genome Center, Department of Pathology, University of Pittsburgh School of Medicine, Pittsburgh, Pennsylvania, USA

## Abstract

**Background:**

HCC is one of the most lethal cancers for humans. Mannosidase alpha class 2A member 1 (MAN2A1)-FER is one of the most frequent oncogenic fusion genes in HCC. In this report, we showed that MAN2A1-FER ectopically phosphorylated the extracellular domains of PDGFRA, MET, AXL, and N-cadherin. The ectopic phosphorylation of these transmembrane proteins led to the activation of their kinase activities and initiated the activation cascades of their downstream signaling molecules.

**Methods:**

A panel of mouse monoclonal antibodies was developed to recognize the ectopic phosphorylation sites of PDGFRA.

**Results and conclusions:**

The analyses showed that these antibodies bound to the specific phosphotyrosine epitopes in the extracellular domain of PDGFRA with high affinity and specificity. The treatment of MAN2A1-FER–positive cancer HUH7 with one of the antibodies called 2-3B-G8 led to the deactivation of cell growth signaling pathways and cell growth arrest while having minimal impact on HUH7ko cells where MAN2A1-FER expression was disrupted. The treatment of 2-3B-G8 antibody also led to a large number of cell deaths of MAN2A1-FER–positive cancer cells such as HUH7, HEPG2, SNU449, etc., while the same treatment had no impact on HUH7ko cells. When severe combined immunodeficiency mice xenografted with HEPG2 or HUH7 were treated with monomethyl auristatin E-conjugated 2-3B-G8 antibody, it slowed the progression of tumor growth, eliminated the metastasis, and reduced the mortality, in comparison with the controls. Targeting the cancer-specific ectopic phosphorylation sites of PDGFRA induced by MAN2A1-FER may hold promise as an effective treatment for liver cancer.

## Graphical abstract

**Figure:**
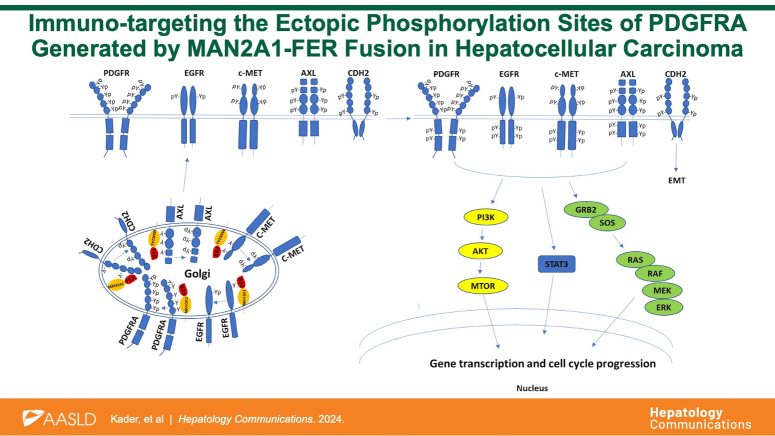

## INTRODUCTION

Liver cancer is one of the most lethal human malignancies. Worldwide, 830,200 people died from primary liver cancers in 2020.^1^ It is expected the mortality of primary liver cancer will rise by more than 55% by 2040. Among the primary liver cancers, HCC accounted for 90% of the liver cancer cases.^2^ Currently, the main approach in treating primary liver cancer is surgical interventions,^2^ either through liver transplantation or surgical resection. However, these options are only available to patients with early-stage liver cancer. Most liver cancers have insidious clinical courses. Many patients are at the advanced stages of liver cancer at the time of diagnosis. Even though extensive progress was made in cancer treatment through molecular targeting and immune modification, few effective treatments were developed for late-stage liver cancers.

FER tyrosine kinase is a downstream signaling molecule for several growth factor receptors and plays a role in cell-cell adhesion. In previous studies,^3^,^4^,^5^,^6^,^7^,^8^ we found that the C-terminus of FER kinase fuses with the N-terminus of mannosidase alpha class 2A member 1 (MAN2A1) and generates a chimera protein called MAN2A1-FER. The fusion genes were detected in 15%–80% of patients with HCC.^5^,^7^ The fusion protein retained the kinase domain of FER and displayed 3.8-fold kinase activity of the native FER protein. The introduction of MAN2A1-FER increased cancer cell proliferation in vitro and promoted cancer growth, invasion, and metastasis in xenografted liver cancer animal models.^7^ When coupled with somatic deletion of Pten, MAN2A1-FER expression induced spontaneous liver cancer in mice.^7^ Despite the apparent oncogenic activity of MAN2A1-FER, the mechanism of its tumor-promoting activity remains unclear. MAN2A1-FER chimera protein is translocated to the Golgi apparatus and ectopically phosphorylates the N-terminus of the epidermal growth factor receptor (EGFR). In this report, we showed that MAN2A1-FER ectopically phosphorylated the extracellular domains of multiple transmembrane proteins and activated the signaling cascades of these proteins. One of these proteins was the platelet-derived growth factor receptor (PDGFR). The ectopic phosphorylation sites of PDGFR were targetable by monoclonal antibodies specific for these abnormally phosphorylated epitopes, which were only present in MAN2A1-FER–positive cancer cells.

## METHODS

### Cell lines and tissue samples

The cell lines used in the study were purchased from the American Type Culture Collection (ATCC) and were cultured and maintained following the manufacturer’s recommendations. They were authenticated every 6 months through a Short Tandem Repeat test and were free of mycoplasma. All cell lines were <10 passages in the laboratory. Rabbit anti-FER antibodies were purchased from Invitrogen Inc (PA5-49788). Mouse anti-MAN2A1 monoclonal antibody was purchased from Santa Cruz Biotechnology, Inc. (SC377204 D5). Sixty adenocarcinomas of the colon and 60 breast cancer formalin-fixed and paraffin-embedded samples were obtained from the Pittsburgh biospecimen core of the University of Pittsburgh in compliance with institutional regulatory guidelines as well as the guidelines of the Declarations of Helsinki and Istanbul. The informed consent exemptions and protocol have been approved by the Institution Review Board of the University of Pittsburgh.

### RNA extraction, cDNA synthesis, TaqMan RT-PCR, and breakpoint Taqman PCR

Total RNA was extracted from cell pellets using TRIzol (Invitrogen).^9^,^10^,^11^,^12^,^13^,^14^,^15^,^16^,^17^ Two micrograms of RNA were used to synthesize the first-strand cDNA with random hexamer primers and Superscript II (Invitrogen). One microliter of each cDNA sample was used for TaqMan PCR with 50 heating cycles at 94°C for 30 seconds, 61°C for 30 seconds, and 72°C for 30 seconds using the primer sequences AGCGCAGTTTGGGATACAGCA/CTTTAATGTGCCCTTATATACTTCACC and the TaqMan probe: 5ʹ/56-FAM/TCAGAAACA/ZEN/GCCTATGAGGGAAATT/3IABkFQ/3ʹ in a thermocycler (QuantStudio 3 real-time PCR system, ThermoFisher, Inc or Mastercycler RealPlex2, Eppendorf, Inc). For breakpoint analysis, 1 μg of genome DNA was used for TaqMan PCR with the following conditions: 50 heating cycles at 94°C for 30 seconds, 61°C for 30 seconds, and 72°C for 30 seconds using the primer sequences CTCAAACTCCTGACCCCGTGA/GAACACAAACCCTTAGGGGGC and the following TaqMan probe: 5ʹ/56-FAM/CCACCTTCT/ZEN/AGCTATTGAGTAGC/3IABkFQ//3ʹ. All positive PCR results were Sanger-sequenced.

### Generation of vectors expressing HisTAG-PDGFRA, HisTAG-MET, HisTAG-N-cadherin, and HisTAG-AXL

For pET28-HisTAG-ΔPDGFRA^aa1-528^ construction, a PCR using the primers indicated in Supplemental Table S1, http://links.lww.com/HC9/B8 and cDNA of PDGFRA (Harvard University, DF/HCC) as the template, in the following condition: 1 cycle of 94^o^C for 1 minute, followed by 35 cycles at 94°C for 30 seconds, 68°C for 30 seconds, and 72°C for 3 minutes. The gel-purified PCR products were digested with *Eco*R1 and NOT1 and ligated into the similarly digested pET28(a)+ vector to generate the pET28-HisTAG-ΔPDGFRA^aa1-528^ vector. To construct pCMV13-HisTAG-ΔPDGFRA^aa1-528^ expression vector, a PCR was performed using the primer pair indicated in Supplemental Table S1, http://links.lww.com/HC9/B8 on the PDGFRA cDNA template. The PCR product was then digested with *Hin*dIII and *Xba*1. The digested product was ligated to a similarly digested pCMV13 vector to create pCMV13-ΔHisTAG-PDGFRA^aa1-528^ vector. Similar construction procedure was performed for pET28-HisTAG-ΔMET^aa1-646^, pCMV13-HisTAG--ΔMET^aa1-646^, pET28-HisTAG-ΔAXL^aa1-451^, pCMV13-HisTAG--ΔAXL^aa1-451^, pET28-HisTAG-ΔCDH2^aa1-724^, and pCMV13-HisTAG--ΔCDH2^aa1-724^, using the respective primers and cDNA templates indicated in Supplemental Table S1, http://links.lww.com/HC9/B8.

### In vitro kinase assay

GST, GST-MAN2A1-FER, and GST-FER were purified by a glutathione column and diluted to 1 ng/μL with 1× kinase assay buffer provided by the manufacturer (Cell Signaling, Inc). This was followed by combining 25 μL GST (50 ng) or GST-MAN2A1-FER (50 ng) or GST-FER (50 ng) and 25 μL substrate (3 μM poly EY[4:1], 1 µg HisTAG-ΔEGFR^aa1650^, 1 µg HisTAG-ΔPDGFRA^aa1-528^, 1 µg HisTAG--ΔMET^aa1-646^, 1 µg HisTAG--ΔCDH2^aa1-724^, or 1 µg HisTAG--ΔAXL^aa1-451^). The solutions were incubated at 37°C for 60 minutes. The reactions were terminated by adding 25 μL of 2N NaOH stop solution to each reaction well. The kinase activities were quantified using the kit and protocols of ADP-Glo Kinase Assay from Promega, Inc.

### Generation of monoclonal antibodies specific for phospho-HisTAG-ΔPDGFRA^aa1-528^

The phospho-HisTAG-ΔPDGFRA^aa1-528^ recombinant protein expressed from HUH7 was purified through the HisTAG column and used as an immunogen. Five female CL6/B6 mice (8 wk old) were each immunized through i.p. injection of 10 µg purified phospho-HisTAG-ΔPDGFRA^aa1-528^ protein (from pCMV13-HisTAG-ΔPDGFRA^aa1-528^ transformed HUH7) that had been emulsified in 0.1 mL of Freund’s adjuvant. These mice were subsequently boosted twice with 10 µg phospho-HisTAG-ΔPDGFRA^aa1-528^ in Freund’s incomplete adjuvant at 21-day intervals. A mouse with high reactivity (>1:100,000 by enzyme-linked immunosorbent assay) was chosen and injected with 20 µg phospho-HisTAG-ΔPDGFRA^aa1-528^ in 0.5 mL PBS 3 days before hybridoma fusion.

Once the animal was sacrificed, the spleen of the immunized mouse was removed. To separate spleen cells, the medium was then gently flushed through the spleen from different angles. Spleen cells were isolated and fused with SP2/0-Ag-14 myeloma cells in the presence of polyethylene glycol (PEG 1500) (Sigma) to produce hybridomas. Fused cells were cultured and selected in RPMI supplemented with 20% fetal bovine serum, 100 U/mL penicillin, 100 mg/mL streptomycin (Bioidea), 1× nonessential amino acids, 1 mM sodium pyruvate (Gibco), and 1× hypoxanthine, aminopterin, and thymidine solution (HAT, 50 stock solution, Sigma). The fused cells were plated onto 96-well plates and screened for monoclonal antibody production by ELISA starting on day 18 after fusion. The ELISA-positive hybridomas were diluted 3 times by limiting dilution technique at 50, 10, and 1 cells/96-well plates. The resultant stable colonies were expanded into 25-cm^2^ Falcon flasks. Hybridoma supernatants were screened for anti-phospho-HisTAG-ΔPDGFRA^aa1-528^ antibody by ELISA and immunoblot analyses. The positive clones were screened for reactivity against unphosphorylated HisTAG-ΔPDGFRA^aa1-528^. Clones positive for phospho-HisTAG-ΔPDGFRA^aa1-528^ (from pCMV13-HisTAG-ΔPDGFRA^aa1-528^ transformed HUH7) but negative for unphosphorylated HisTAG-ΔPDGFRA^aa1-528^ (from *Escherichia coli or HUH7ko* which was generated in our previous study^7^) were selected. These clones were further confirmed by immunofluorescence staining and immunoblot analyses. Five positive clones were selected for further assays. For 2-3B-G8-MMAE (monomethyl auristatin E) conjugation, the antibody MMAE conjugation kit (Mosiac, Inc) was used. MMAE conjugation helps to induce mitotic arrest when the antibody is internalized.^18^ The conjugation procedure followed the manufacturer’s recommendation.

### Antibody-antigen binding assays

The antibody at various concentrations (78 ng–10 µg/mL) was dissolved in 0.1 M potassium phosphate, 2 mM EDTA, pH 7.8, supplemented with 10 mg/mL bovine serum albumin. The solution was transferred and incubated for 1 hour at 20°C into the wells of a microtitration plate previously coated with phospho-HisTAG-ΔPDGFRA^aa1-528^ (at 100 µg/well in 50 mM sodium carbonate, pH 9.6 for 15 h at 4°C) for ELISA assay. The binding affinity was calculated using Scatchard analysis.^19^

### Bromodeoxyuridine cell cycle assays and cell death assay

The procedure of bromodeoxyuridine labeling was described previously.^7^,^11^,^13^,^16^,^20^,^21^,^22^,^23^,^24^ Cells were synchronized by culture in an fetal bovine serum-free medium for 48 hours, followed by replenishment with a medium containing 10% fetal bovine serum and bromodeoxyuridine for 4 hours. Cells were harvested for analysis with a FITC-bromodeoxyuridine antibody and propidium iodide nuclear staining (BD Biosciences). The distribution of cells in different cell cycle phases was analyzed using flow cytometry (BD FACSCalibur). For cell death assays, cells were treated with 2-3B-G8 antibody (10 µg/mL) or with PDGFRA (10 µg/mL, MA5-38592, Invitrogen, Inc), or with nonspecific mouse IgG (10 µg/mL) as the control. Cell cultures at 70%–80% confluence were treated with these antibodies for 18–24 hours. For the siRNA study, siPDGFRA (20 nM, Life Technology Inc) and universal negative control (20 nM, Sigma-Aldrich Inc) were transfected into cell culture through Lipofectamine 3000 for 48 hours. Cells were then harvested for cell death analysis with a phycoerythrin-annexin V apoptosis assay kit (BD Biosciences). Cells were resuspended in 100 µL of annexin V binding buffer (Invitrogen) and incubated with 5 µL of phycoerythrin-conjugated annexin V and 5 µL of propidium iodide for 20 minutes in the dark at room temperature. The binding assays were terminated by the addition of 400 µL of annexin V binding buffer. FACS analysis was performed using a BD FACSCalibur (BD Sciences). Ten thousand cells were acquired and sorted. WinMDI 2.9 software (freeware from Joseph Trotter) was used to analyze the data.

### Immunoblotting, immunostaining, and immunofluorescence staining

Immunoblotting procedures were previously described.^12^,^20^,^21^,^24^,^25^,^26^ For immunofluorescence staining, HUH7 or HUH7ko or HEP3B or HEP3B-MAN2A1-FER cells were fixed with 4% paraformaldehyde for 15 minutes. These cells were blocked with 10% goat serum 0.4% triton x-100 in PBS for 1 hour, followed by incubation with 2-3B-G8, 1-3C-F11, 2-3C-C5, 2-6B-D8, 1-4B-B10, or IgG antibody (100 ng/mL) in 2% goat serum in PBS for 1 hour. The cells were then incubated with FITC-conjugated goat anti-mouse antibody in 2% goat serum in PBS for an hour, followed by a DAPI incubation for 10 minutes. The imaging was taken with a fluorescence microscope (Olympus) or assayed for flow cytometry (BD FACSCalibur).

### Xenografted tumor growth and treatment

Male severe combined immunodeficiency (SCID) mice were used. The xenografting procedure was described previously.^11^,^12^,^13^,^16^,^27^,^28^,^29^ Briefly, **~**5×10^6^ viable HepG2, HUH7, and HUH7/ko cells suspended in 0.2 mL of Hanks’ balanced salt solution (Krackeler Scientific, Inc) were subcutaneously implanted in the abdominal flanks of SCID mice to generate 1 tumor per mouse. Two weeks after xenografting of HEPG2, the tumors reached an average size of 752 mm^3^. These mice were treated with 2-3B-G8-MMAE or IgG-MMAE (4 mg/kg) 2 times a week through tail vein applications. After 5 weeks, all surviving mice were killed, and necropsies were performed. For mice treated with control IgG, necropsies were performed when mice died from the xenografted cancers. Invasion was defined as tumor piercing through the confine of subcutaneous tissue. Metastasis was defined as tumor nodules in other organs identified in the autopsy separated from the primary tumor of the original inoculation site. The final measurements of tumor volumes exclude tumors invading the internal organ and metastasis. For HUH7 or HUH7ko xenografted mice, the treatment started when the tumor reached the average sizes of 117 or 107 mm^3^ (10 d), respectively. The earlier treatment of HUH7 tumors was essential due to the rapid growth nature of the cancer cell line. Similar treatment schemes as above were applied to these animals. All HUH7 control-treated animals died within 2 weeks of the treatment. The treatment and nontreatment groups were randomized and blinded to the researchers. These procedures have been approved by the Institutional Animal Care and Use Committee of the University of Pittsburgh.

## RESULTS

### Expression of MAN2A1-FER is frequent in human cancer cell lines

**FIGURE 1:**
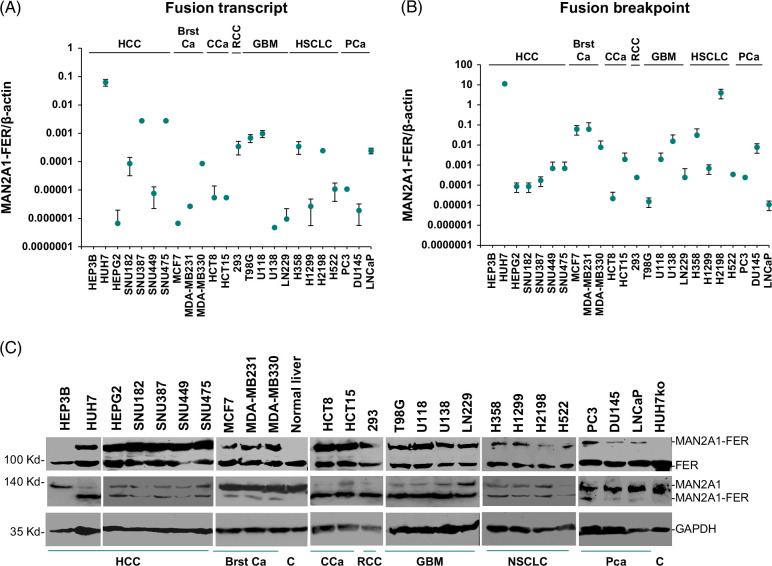
Frequent expression of MAN2A1-FER in liver cancer and other cancer cell lines. (A) Detection of MAN2A1-FER fusion transcripts in cell lines of HCC, Brst Ca, CCa, RCC, GBM, NSCLC, and PCa. Taqman qRT-PCR assays were performed in duplicate for each cell line. The copy number of the fusion transcript was normalized to that of β-actin from the same samples. Standard deviations were shown. (B) Detection of the breakpoint sequence in the genomes of cancer cell lines as of (A). Taqman real-time qPCR assays were performed in duplicate for each cell line. The copy number of the fusion transcript was normalized to that of β-actin from the same samples. Standard deviations were shown. (C) Detection of MAN2A1-FER fusion protein in the immunoblotting of cell lines by rabbit polyclonal antibodies specific for the C-terminus of FER and N-terminus of MAN2A1. The immunoblotting with antibody specific for GAPDH was loading control. C denotes control cells negative for MAN2A1-FER. Abbreviations: Brst Ca, breast cancer; CCa, colon cancer; GAPDH, glyceraldehyde-3-phosphate dehydrogenase; GBM, glioblastoma multiforme; MAN2A1, mannosidase alpha class 2A member 1; NSCLC, non-small–cell lung cancer; PCa, prostate cancer; qRT-PCR, quantitative real time reverse transcription polymerase chain reaction; RCC, renal cell carcinoma.

The generation of MAN2A1-FER gene fusion is the result of chromosome recombination in the 5q21-22 region.^6^,^7^,^8^ The expression of MAN2A1-FER was frequently detected in primary liver cancer and serum samples.^5^,^7^ To investigate the expression of MAN2A1-FER in liver cancer cell lines, 7 HCC cell lines were tested for MAN2A1-FER expression using TaqMan quantitative real time reverse transcription polymerase chain reaction. Six cell lines including HUH7, SNU387, SNU449, SNU475, SNU182, and HEPG2 were positive for MAN2A1-FER mRNA, while HEP3B was negative (Figure 1A and Supplemental Figure S1A, http://links.lww.com/HC9/B9). The expression levels of MAN2A1-FER mRNA varied significantly among different cell lines, with the highest MAN2A1-FER fusion mRNA expression detected in HUH7 cells and relatively low expression in MCF7 and U138 cells. The chromosome breakpoint of MAN2A1-FER was previously identified in HUH7 cells. To investigate whether other HCC cell lines also contained the same breakpoint as HUH7, Taqman quantitative real time reverse transcription polymerase chain reaction and Sanger’s sequencing were performed on the genome DNA of SNU387, SNU449, SNU475, SNU182, and HEPG2 cells. The results indicated that all these cell lines positive for MAN2A1-FER had an identical breakpoint, suggesting a common mechanism of chromosome recombination that led to the generation of MAN2A1-FER gene fusion (Figure 1B and Supplemental Figure S1B, http://links.lww.com/HC9/B9). Interestingly, the relative quantity of MAN2A1-FER breakpoint to β-actin also varied among cell lines: HUH7 and H2198 cell lines contained high numbers of breakpoints, while LNCaP and T98G had very few copies of the same breakpoint. Immunoblot analysis using the antibodies specific for the C-terminus of FER confirmed the fusion protein expression of MAN2A1-FER in all the HCC cell lines positive for the fusion genes while negative for HEP3B cells, HUH7ko cells where MAN2A1-FER expression was disrupted, and a normal liver sample. All samples expressed the wild-type FER. A similar finding was obtained for the antibodies specific to MAN2A1 (Figure 1C).

To investigate whether MAN2A1-FER gene fusion is widespread among human cancer cell lines, 3 prostate cancer (PC3, DU145, and LNCaP), 4 glioblastoma multiforme (98G, U118, U138, and LN 229), 3 breast cancer (MCF7, MDA-MB231, and MDA-MB330), 4 non-small–cell lung cancer (H358, H1299, H1298, and H522), 2 colon cancer (HCT8 and HCT15), and 1 renal cell carcinoma (293) cell lines were analyzed for MAN2A1-FER mRNA expression. The results showed that all these cancer cell lines were positive for MAN2A1-FER (Figure 1A and Supplemental Figure S1A, http://links.lww.com/HC9/B9). All cancer cell lines positive for MAN2A1-FER mRNA shared the same breakpoint in the chromosome level (Figure 1B and Supplemental Figures S1B, C, http://links.lww.com/HC9/B9). A distinct MAN2A1-FER chimera protein in the cell lines positive for MAN2A1-FER gene fusion was detected using the antibodies specific for the C-terminus of FER tyrosine kinase (Figure 1C). High-frequency expressions of MAN1A1-FER were also detected in colon and breast cancers (Supplemental Figure S2, http://links.lww.com/HC9/B9). These results suggest that MAN2A1-FER was frequent in human malignancies.

### MAN2A1-FER kinase phosphorylated multiple transmembrane proteins in vitro and in vivo

**FIGURE 2:**
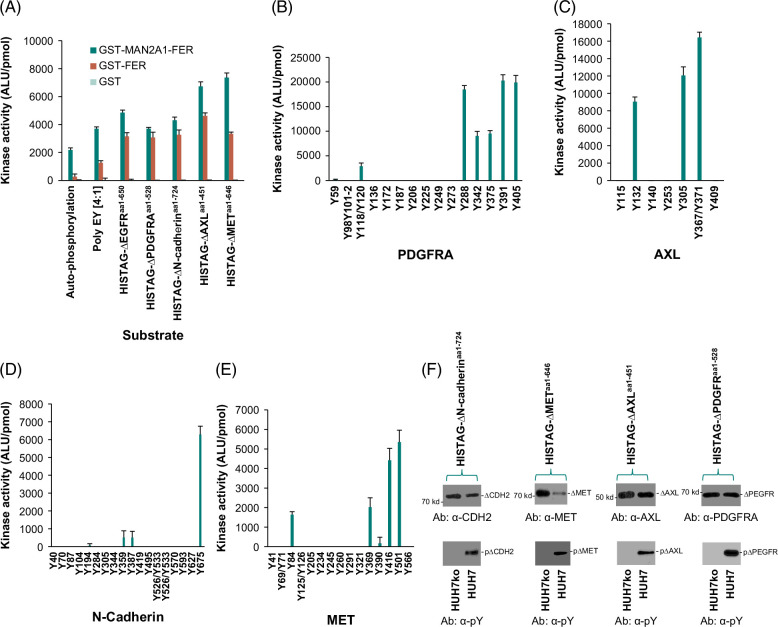
MAN2A1-FER phosphorylated the extracellular domains of EGFR, PDGFR, MET, AXL, and CDH2 in vitro and in vivo. (A) In vitro kinase assays of GST-FER and GST-MAN2A1-FER on substrates Poly E:Y (1:4), HisTAG-ΔEGFR^aa1650^, His TAG-ΔPDGFRA^aa1-528^, HisTAG-ΔCDH2^aa1-724^, HisTAG-ΔAXL^aa1-451^, or HisTAG-ΔMET^aa1-646^. Each experiment was performed in triplicate. Standard deviations were shown. (B) In vitro kinase assays of GST-MAN2A1-FER on tyrosine residue containing peptides (Supplemental Table S2, http://links.lww.com/HC9/B8) corresponding to PDGFRA extracellular domain. Triplicate experiments were performed. Standard deviations were shown. (C) In vitro kinase assays of GST-MAN2A1-FER on tyrosine residue containing peptides (Supplemental Table S2, http://links.lww.com/HC9/B8) corresponding to AXL extracellular domain. Triplicate experiments were performed. Standard deviations were shown. (D) In vitro kinase assays of GST-MAN2A1-FER on tyrosine residue containing peptides (Supplemental Table S2, http://links.lww.com/HC9/B8) corresponding to CDH2 extracellular domain. Triplicate experiments were performed. Standard deviations were shown. (E) In vitro kinase assays of GST-MAN2A1-FER on tyrosine residue containing peptides (Supplemental Table S2, http://links.lww.com/HC9/B8) corresponding to MET extracellular domain. Triplicate experiments were performed. Standard deviations were shown. (F) MAN2A1-FER phosphorylated the extracellular domains of CDH2, MET, AXL, and PDGFR in vivo. The His TAG-ΔPDGFRA^aa1-528^, HisTAG-ΔCDH2^aa1-724^, HisTAG-ΔAXL^aa1-451^, or HisTAG-ΔMET^aa1-646^ proteins were expressed through pCMV13 recombination constructs in HUH7 or HUH7ko cells where MAN2A1-FER was disrupted. The recombinant proteins were purified by the HisTAG column. The proteins were then immunoblotted with antibodies specific for the recombinant proteins (top) and the phosphotyrosine (bottom). Abbreviation: MAN2A1, mannosidase alpha class 2A member 1.

MAN2A1-FER chimera protein is mostly localized in the Golgi apparatus in an orientation that may contact the extracellular domains of multiple transmembrane proteins.^7^ To investigate whether MAN2A1-FER induced the phosphorylation of extracellular domains of transmembrane proteins, we ligated the extracellular domains of PDGFRA (aa1-528), MET (aa1-646), AXL (aa1-451), and N-cadherin (aa1-723) into pET28a(+) vector to produce HisTAG fusion proteins in *E. coli*. Kinase analysis indicated that both GST-FER and GST-MAN2A1-FER induced the phosphorylation of these truncated proteins, similar to the results obtained from synthetic substrate poly E:Y (4:1) or the N-terminus of EGFR (Figure 2A). The N-terminus of PDGFRA contained 17 tyrosine residues. To examine which tyrosine residue was phosphorylated, a series of 24–25 amino acid peptides corresponding to the regions containing tyrosine residues in the extracellular domain of PDGFRA were applied to the GST-MAN2A1-FER tyrosine kinase assay. The results showed that significant phosphorylation occurred at 7 tyrosine residues (Y180/Y120, Y288, Y342, Y375, Y391, and Y405; Figure 2B). For the extracellular domain of AXL, significant phosphorylation was identified at 4 positions (Y132, Y305, and Y367/Y371; Figure 2C), while for N-cadherin (Figure 2D) and MET (Figure 2E), the phosphorylation occurred at 3 (Y359, Y387, and Y675) and 5 (Y84, Y369, Y390, Y416, and Y501) positions, respectively. To examine whether the ectopic phosphorylation of PDGFRA N-terminus occurred in vivo, HisTAG-ΔPDGFR^aa1-528^ was constructed into pCMV13 vector to express as a truncated PDGFRA protein in HUH7 where MAN2A1-FER was positive. As shown in Figure 2F, the extracellular domain of PDGFRA was positive for tyrosine phosphorylation, as demonstrated by the recognition of the protein with an antiphosphotyrosine antibody. In contrast, the immunoblotting became negative in HUH7ko cells, where MAN2A1-FER expression was disrupted. Coimmunoprecipitation analyses showed that MAN2A1-FER-FLAG and PDGFRA were bound to each other at the cellular level (Supplemental Figure S3, http://links.lww.com/HC9/B9). Similar findings were identified in the extracellular domains of AXL (HisTAG-ΔAXL^aa1-451^), MET (HisTAG-ΔMET^aa1-646^), and N-Cadherin (HisTAG-ΔN-Cadherin^aa1-723^). These results suggest that the phosphorylation of extracellular domains of PDGFRA, AXL, MET, and N-cadherin were MAN2A1-FER dependent.

### MAN2A1-FER activated signaling cascades of multiple transmembrane proteins

**FIGURE 3:**
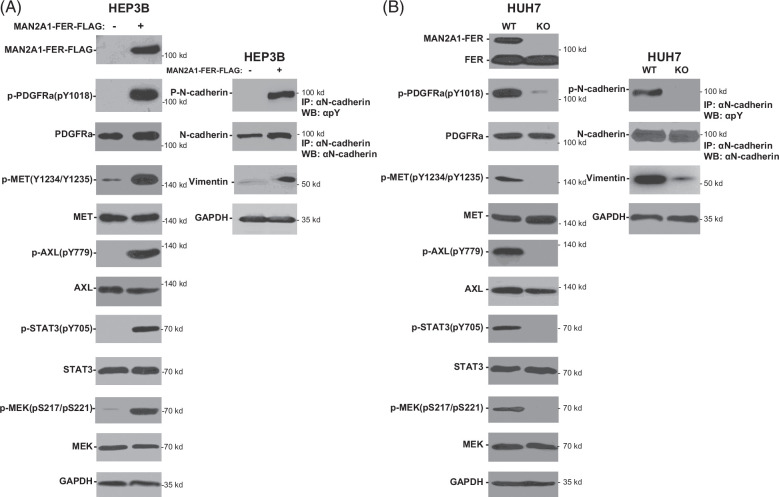
MAN2A1-FER activated the signaling cascades of PDGFR, EMT, AXL, and CDH2. (A) Immunoblotting of indicated proteins and phospho-proteins in HEP3B cells transformed with pCNDA4-MAN2A1-FER-FLAG/pCDNA6TO and induced with or without tetracycline. (B) Immunoblotting of indicated proteins and phospho-proteins in HUH7 cells or HUH7ko cells where MAN2A1-FER expression was disrupted by CRISPR-cas9 editing.^7^ Abbreviation: GAPDH, glyceraldehyde-3-phosphate dehydrogenase; MAN2A1, mannosidase alpha class 2A member 1.

To investigate the impact of ectopic phosphorylation on PDGFRA, HEP3B cells where MAN2A1-FER was negative were transformed with pCDNA4-MAN2A1-FER-FLAG/pCDNA6TO. The induction of expression of MAN2A1-FER-FLAG by tetracycline showed a dramatic increase in phosphorylation of tyrosine 1018 in PDGFRA. This was accompanied by an increase in the phosphorylation of MEK and STAT3 (Figure 3A). The expression of MAN2A1-FER-FLAG in HEP3B cells also increased the phosphorylation of tyrosine 1234/1235 of MET and tyrosine 779 of AXL and the activations of their common downstream signaling molecules of STAT3 and MEK. To examine whether the removal of MAN2A1-FER had the opposite effect on the activation of PDGFR, MET, and AXL, HUH7ko cells where MAN2A1-FER was disrupted were examined for the phosphorylation of Y1018 of PDGFRA. The results showed a significant decrease in PDGFRA activation based on the Y1018 phosphorylation level (Figure 3B). The downstream signaling molecules MEK and STAT3 also showed deactivation. The deactivation of MET and AXL signaling was also identified. The phosphorylation of N-cadherin by MAN2A1-FER-FLAG in HEP3B cells was accompanied by increased expression of vimentin (Figure 3A), while disruption of MAN2A1-FER in HUH7ko cells reduced the expression of the same protein (Figure 3B). These results indicated that MAN2A1-FER might be a key molecule in promoting multiple pro-growth and epithelial-mesenchyme transition in liver cancer cells.

### Mouse monoclonal antibody specific for ectopic phosphorylation of PDGFRA

**FIGURE 4:**
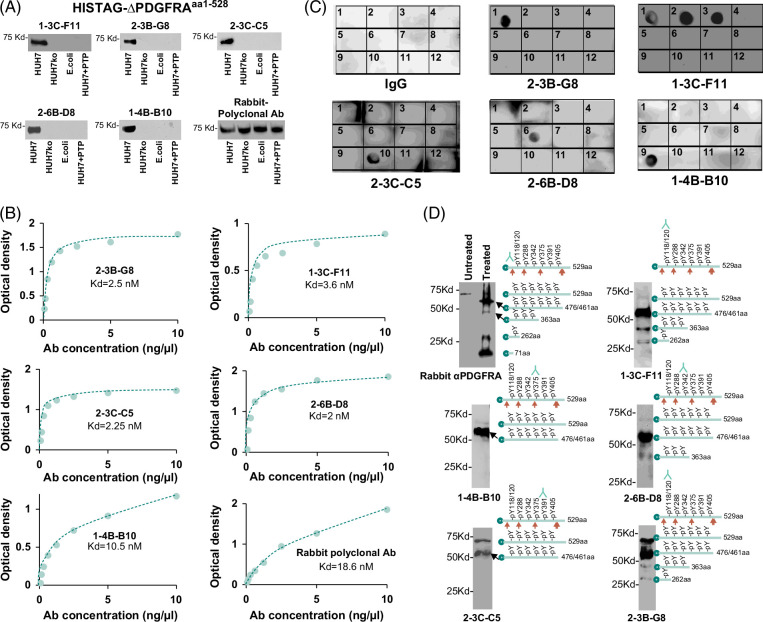
Binding epitopes and binding affinity of antibodies from hybridoma clones 2-3B-G8, 1-3C-F11, 2-3C-C5, 2-6B-D8, and 1-4B-B10. (A) Antibodies from clones 2-3B-G8, 1-3C-F11, 2-3C-C5, 2-6B-D8, and 1-4B-B10 bound phosphorylated HisTAG-ΔPDGFRA^aa1-528^ from HUH7 but not the unphosphorylated counterparts from *E. coli* or HUH7ko nor phosphorylated HisTAG-ΔPDGFRA^aa1-528^ treated with human protein tyrosine phosphatase 1B (PTP). Immunoblotting was performed using the indicated antibodies on HisTAG column-purified HisTAG-ΔPDGFRA^aa1-528^ protein expressed from HUH7, HUH7ko, or *E. coli*. HisTAG-ΔPDGFRA^aa1-528^ expressed from HUH7 but treated with PTP is a specificity control. (B) Antibodies from clones 2-3B-G8, 1-3C-F11, 2-3C-C5, 2-6B-D8, and 1-4B-B10 bound to phosphorylated HisTAG-ΔPDGFRA^aa1-528^ with high affinity. Binding assays between the indicated antibodies and the phosphorylated HisTAG-ΔPDGFRA^aa1-528^ from HUH7 were performed. The data were analyzed using standard Scatchard analysis. The dissociation constant (kd) was indicated for each antibody. (C) Antibodies from clones 2-3B-G8, 1-3C-F11, 2-3C-C5, 2-6B-D8, and 1-4B-B10 bound to specific phosphorylated tyrosine in the extracellular domain of PDGFRA. Immune dot blot analyses were performed on the tyrosine-phosphorylated peptides and their corresponding unphosphorylated controls (Supplemental Table S3, http://links.lww.com/HC9/B8) with the indicated antibodies. (D) Mapping of the antibody epitopes of 2-3B-G8, 1-3C-F11, 2-3C-C5, 2-6B-D8, and 1-4B-B10 in vivo. The protein extract of HUH7 transformed with pCMV13-HisTAG-ΔPDGFRA^aa1-528^ was partially digested with benzoic acid. The digested HisTAG-ΔPDGFRA^aa1-528^ fragments were purified by HisTAG column. Immunoblotting was performed after the protein fragments were resolved in 15% SDS-PAGE. HisTAG-ΔPDGFRA^aa1-528^ protein was indicated by blue ball (HisTAG) and bar (ΔPDGFRA^aa1-528^). The benzoic acid cleavage (tryptophan) positions in HisTAG-ΔPDGFRA^aa1-528^ were indicated by red arrows. The specific antibody epitope was indicated by a vertically flipped green Y.

The ectopic phosphorylation in the extracellular domains of growth factor receptors by MAN2A1-FER may present a targeting opportunity against these pathological phosphorylations. Next, we immunized the C57Bl6 mice with HisTAG-ΔPDGFRA^aa1-528^ (ectopically phosphorylated by MAN2A1-FER) expressed from HUH7 cells. After 3 rounds of immunization and antibody screening, the antiserum positive for HisTAG-ΔPDGFRA^aa1-528^ of HUH7 from 1 of the 5 animals was obtained. Hybridoma cells were generated after fusing the spleen lymphocytes from the animal with Sp2/0-Ag14 cells. The hybridoma clones were screened for antibodies positive for HisTAG-ΔPDGFRA^aa1-528^ of HUH7 but negative for HisTAG-ΔPDGFRA^aa1-528^ of HUH7ko or HisTAG-PDGFRA^aa1-528^ of *E. coli*. As shown in Figure 4A, 5 hybridoma clones were found to produce antibodies specific for the ectopically phosphorylated HisTAG-ΔPDGFRA^aa1-528^ from HUH7 cells but unreactive with HisTAG-ΔPDGFRA^aa1-528^ from HUH7ko or *E. coli*. To examine the binding affinity of these antibodies for the phosphorylated PDGFRA N-terminus, enzyme-linked immunosorbent assays were performed using the antibodies with different concentrations of the HisTAG-ΔPDGFRA^aa1-528^ from HUH7 cells. The results showed that these antibodies have dissociation constant (kd) values ranging from 2 to 10 nM (Figure 4B), suggesting a high affinity of the antibodies with the antigen.

To identify which phosphotyrosine residue in the extracellular domain of PDGFRA these antibodies bound, a series of phospho-peptides corresponding to 6 potential phosphotyrosine targets and their unphosphorylated counterparts were synthesized. As shown in Figure 4C, the antibody from clone 2-3B-G8 appeared to bind to phospho-peptide (pY118/pY120) corresponding to aa106-130 of PDGFRA. Interestingly, the antibody did not bind the same peptides that contain only pY118 (well 2) or pY120 (well 3), nor the unphosphorylated peptide (well 12). On the other hand, the antibody from the 1-3C-F11 clone bound to the same peptide containing pY118/pY120 (well 1), pY118 (well 2), or pY120 (well 3), but not the unphosphorylated control peptide (well 12). The antibody from clone 2-3C-C5 recognized the tyrosine-phosphorylated (pY391, well 10) peptide corresponding to aa382-402 of PDGFRA but not the unphosphorylated counterpart (well 11, Figure 4C). The antibody from clone 2-6B-D8 was specific for phosphotyrosine 342 (well 6) of PDGFRA, while the antibody from clone 1-4B-B10 was for phosphotyrosine 375 (well 9, Figure 4C). To examine whether these antibodies also recognized the same epitopes in vivo, the HisTAG-ΔPDGFRA^aa1-528^ from HUH7 cells were partially digested with benzoic acid, which specifically cleaved at the tryptophan residue in a protein sequence. The partially digested HisTAG-ΔPDGFRA^aa1-528^ was purified through the HisTAG column, resolved in 15% sodium dodecyl sulfate-polyacrylamide gel electrophoresis, and immunoblotted with each of these monoclonal antibodies. As shown in Figure 4D, all the results in vivo were consistent with those in vitro.

### The binding of monoclonal antibodies specific for the ectopically phosphorylated extracellular domain of PDGFRA was MAN2A1-FER fusion-dependent

**FIGURE 5:**
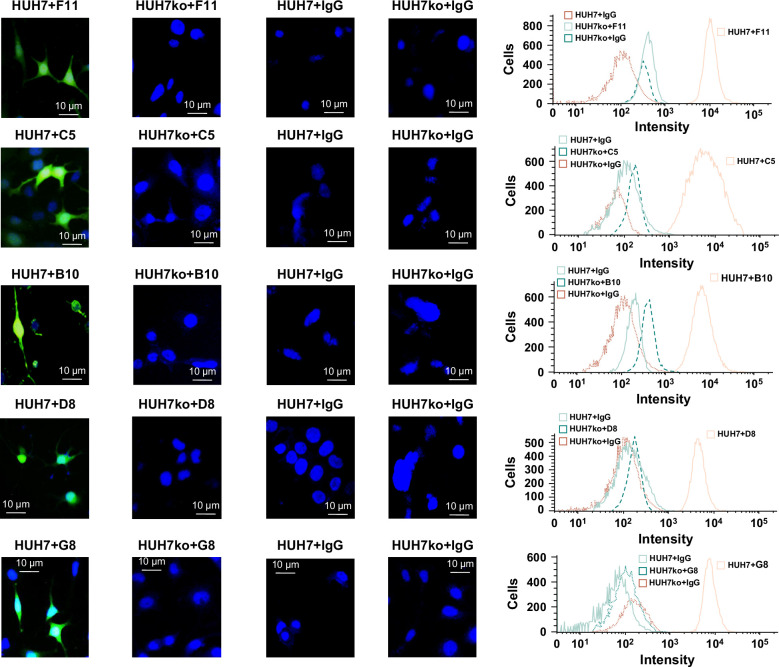
Antibodies from hybridoma clones 1-3C-F11, 2-3C-C5, 2-6B-D8, and 1-4B-B10 bound MAN2A1-FER–positive cells in vivo. Left: Images of immunofluorescence staining of HUH7 or HUH7ko cells where MAN2A1-FER was disrupted with IgG or antibodies from hybridoma clones 1-3C-F11 (F11, top), 2-3C-C5 (C5, second from the top), 1-4B-B10 (B10, third from the top), 2-6B-D8 (D8, fourth from the top), and 2-3B-G8 (bottom). Right: Flow cytometry analyses of the immunofluorescence staining from the left. The conditions of the immunofluorescence assays were described in detail in the Methods section. Abbreviation: MAN2A1, mannosidase alpha class 2A member 1.

**FIGURE 6:**
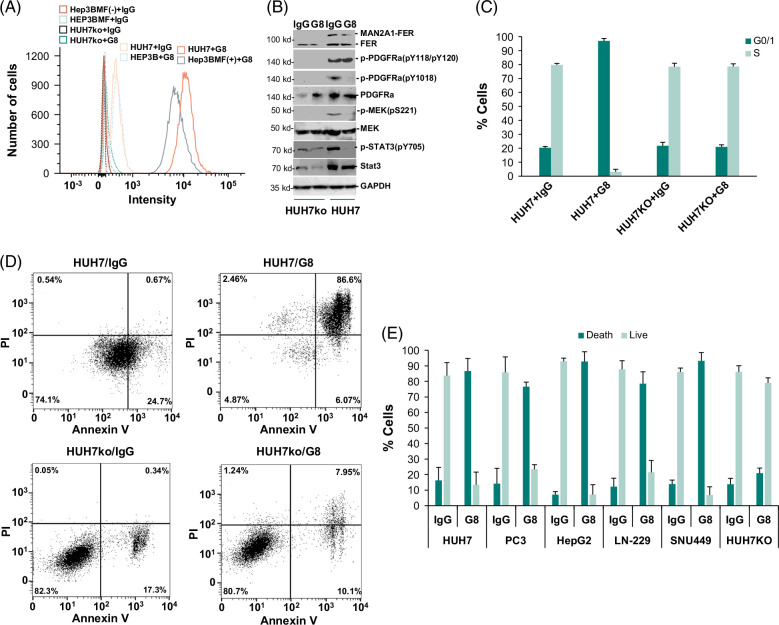
Antibodies from hybridoma clone 2-3B-G8 inhibited PDGFRA activation, caused cell growth arrest, and induced cell death of cancer cells positive for MAN2A1-FER. (A) Flow cytometry analyses of the immunofluorescence staining of antibodies from hybridoma clone 2-3B-G8 (G8) or nonspecific IgG. HEP3BMF(+) indicated HEP3B cells transformed with pCDNA4-MAN2A1-FER-FLAG/pCDNA6TO and induced with tetracycline, while HEP3BMF(−) was the uninduced control. (B) Immunoblotting of protein extracts from HUH7 or HUH7ko cells treated with 10 ng/mL IgG or 2-3B-G8 (G8). Antibodies specific for FER C-terminus, PDGFRA, phospho-PDGFRA (pY118/pY120), phospho-PDGFRA (pY1018), MEK, phospho-MEK(pS221), STAT3, phospho-STAT3(pY705), and GAPDH were applied. (C) Antibody from hybridoma clone 2-3B-G8 induced cell growth arrest in HUH7 cells but not HUH7ko cells. BrdU labeling of cell cycle analyses was performed. Triplicate experiments were performed. Standard deviations were shown. (D) Scatter plot analyses of annexin V and propidium iodide staining of HUH7 or HUH7ko cells treated with 10 ng/mL IgG or 2-3B-G8 (G8). (E) Antibody from hybridoma clone 2-3B-G8 caused cell death of MAN2A1-FER–positive cancers (HUH7, PC3, HepG2, LN-229, and SNU449) but had minimal impact on MAN2A1-FER–negative HUH7ko cells. Triplicate experiments were performed. Standard deviations were shown. Abbreviations: BrDU, bromodeoxyuridine; GAPDH, glyceraldehyde-3-phosphate dehydrogenase; MAN2A1, mannosidase alpha class 2A member 1.

To investigate the antibodies specific for the ectopically phosphorylated extracellular domain bound PDGFRA epitopes in live liver cancer cells, unfixed HUH7 cells were immunostained with antibodies from 1-3C-F11, 2-3C-C5, 1-4B-B10, 2-6B-D8, and 2-3B-G8 (Figures 5 and 6A). The results showed strong immunofluorescence staining of most HUH7 cells by all these antibodies. However, the immunostaining activity of these antibodies disappeared when the expression of MAN2A1-FER was disrupted in HUH7 cells, suggesting that the binding of these antibodies with HUH7 cells was dependent on the expression of MAN2A1-FER fusion protein.

### Monoclonal antibody from hybridoma clone 2-3B-G8 induced growth arrest and cell death of HUH7 cells

Antibody 2-3B-G8 stained strongly on HUH7 cells or HEP3B cells transformed with MAN2A1-FER-FLAG (Figure 6A) but was largely negative on HEP3B cells which were negative for MAN2A1-FER fusion or HUH7ko where MAN2A1-FER was disrupted in genome level. To investigate the impact of 2-3B-G8 antibody on signal transduction mediated by MAN2A1-FER, HUH7 cells were treated with 10 ng/mL 2-3B-G8 antibody for 6 hours. The results showed that treatment of 2-3B-G8 antibody significantly reduced the activation of PDGFRA, MEK, and STAT3 in HUH7 cells in comparison with the controls (Figure 6B). The impact of 2-3B-G8 antibody on HUH7 cells was concordant with the presence of pY118/pY120 epitope in PDGFRA. In contrast, the impact of 2-3B-G8 antibody on MEK and STAT3 activation was minimal in HUH7ko cells where pY118/pY120 epitope was absent. Cell cycle analyses showed that the treatment of 2-3B-G8 antibody on HUH7 blocked cell entry into the S phase by 25.6 fold (3.1% vs. 79.7%, *p* < 0.001) in comparison with the nonspecific IgG controls (Figure 6C). However, such dramatic impact disappeared when MAN2A1-FER was disrupted in HUH7 cell line, indicating that the growth inhibition by the 2-3B-G8 antibody was entirely dependent on MAN2A1-FER expression. To examine the consequence of 2-3B-G8 treatment on cell lines that were positive for MAN2A1-FER, several human cancer cell lines positive for MAN2A1-FER, including HUH7, HEPG2, SNU449, LN-229, and PC3, along with HUH7ko were examined for cell death after the treatment of 2-3B-G8 antibody. As shown in Figure 6D, the treatment of 2-3B-G8 antibody on HUH7 cells induced ~3.7 fold increase in cell death (95.1% vs. 25.5%, *p* < 0.01). However, this impact was largely eliminated in HUH7ko cells (19.3% vs. 17.7%, *p* = 0.27). Similar large increases in cell death were found for other liver cancer cell lines such as HEPG2 (13.1 fold, *p* < 0.01) and SNU449 (6.7 fold, *p* < 0.01), prostate cancer cell line PC3 (5.4 fold, *p* < 0.01) and glioblastoma cell line LN-229 (7.6 fold, *p* < 0.01), when treated with 2-3B-G8 antibody (Figure 6E). In contrast to 2-3B-G8, the treatment of neutralizing PDGFRA antibody induced cell growth arrest and cell death in HUH7 cells, which was positive for MAN2A1-FER but also induced similar cell growth arrest and cell deaths in MAN2A1-FER–negative HEP3B and HUH7ko cells (Supplemental Figure S4, http://links.lww.com/HC9/B9). Similar to PDGFRA neutralizing antibodies, siRNA specific for PDGFRA and tyrosine kinase inhibitor specific for PDGFR (AG1295) arrested cell growth and induced cell death of HUH7, HUH7ko, and HEP3B cells indiscriminately (Supplemental Figure S3, http://links.lww.com/HC9/B9). The impact of these treatments appeared MAN2A1-FER independent.

### Monoclonal antibody from hybridoma clone 2-3B-G8 alleviated tumor burden, reduced metastases, and decreased mortality of xenografted liver cancer cell lines

**FIGURE 7:**
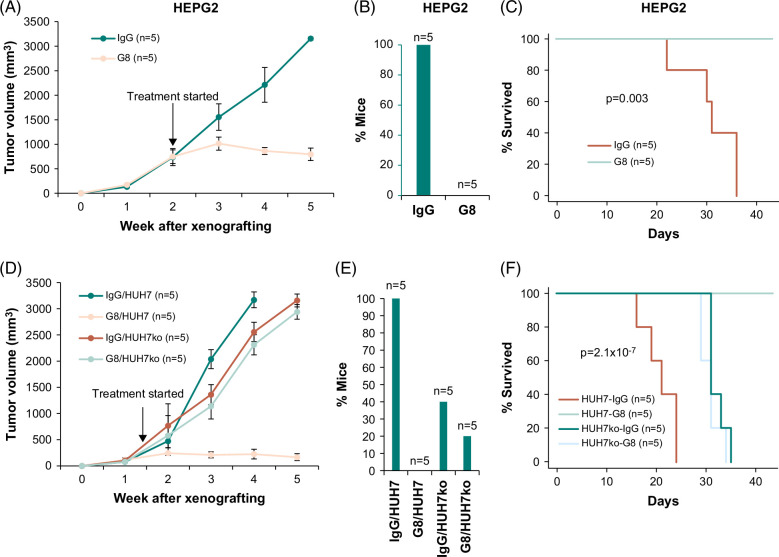
The therapeutic effect of an antibody from hybridoma clone 2-3B-G8 on HEPG2 and HUH7 xenografted cancers. (A) Antibody from hybridoma clone 2-3B-G8 reduced the tumor burden of HEPG2 xenografted cancer in SCID mice. Mice (n = 5) were treated with 2-3B-G8-MMAE 14 days after HEPG2 xenografting. Tail vein injection of the antibody was applied twice a week. IgG-MMAE treatment (n = 5) was the placebo control. (B) 2-3B-G8-MMAE eliminated the invasion/metastasis from HEPG2 cancer. Invasion was defined as tumor piercing through the confine of subcutaneous tissue. Metastasis was defined as tumor nodules in other organs identified in the autopsy separated from the primary tumor of the original inoculation site. (C) 2-3B-G8-MMAE reduced the mortality of the animals xenografted with HEPG2 cancer. Kaplan-Meier analyses were used to assess the animals’ survival over time. Mortality includes deaths of animals or animals in severe distress due to tumors that warranted euthanasia, consistent with the recommendation of the Panel on Euthanasia of the American Veterinary Medical Association. Log-Rank test was performed. The *p* value was indicated. (D) 2-3B-G8-MMAE reduced the tumor burden of HUH7 but not HUH7ko cancer, where MAN2A1-FER was disrupted. Mice (n = 5) were treated with 2-3B-G8-MMAE 14 days after HUH7 xenografting. Tail vein injection of the antibody was applied twice a week. IgG-MMAE treatment (n = 5) was the placebo control. Mice xenografted with HUH7ko cells treated with IgG-MMAE or 2-3B-G8-MMAE were the specificity controls. (E) 2-3B-G8-MMAE reduced invasion/metastasis of HUH7 but not HUH7ko cancers. Invasion was defined as tumor piercing through the confine of subcutaneous tissue. Metastasis was defined as tumor nodules in other organs identified in the autopsy separated from the primary tumor of the original inoculation site. (F) 2-3B-G8-MMAE decreased mortality of HUH7 but not HUH7ko cancers. Kaplan-Meier analyses were used to assess the animals’ survival over time. Mortality includes deaths of animals or animals in severe distress due to tumors that warranted euthanasia, consistent with the recommendation of the Panel on Euthanasia of the American Veterinary Medical Association. Log-Rank test was performed. The *p* value was indicated. Abbreviations: MMAE, monomethyl auristatin E; SCID, severe combined immunodeficiency.

To investigate whether the 2-3B-G8 antibody had therapeutic value in treating cancer positive for MAN2A1-FER, liver cancer cell line HEPG2 was xenografted into the subcutaneous region of SCID mice. When the HEPG2 tumor reached an average size of 752 mm^3^, the mice were treated with the MMAE conjugated 2-3B-G8 antibody through tail vein injection twice a week. As shown in Figure 7A, the treatment of 2-3B-G8-MMAE blunted the increase in tumor volume and produced a mild regression of the tumor size from its peak. In contrast, the IgG-MMAE-treated HEPG2 tumors continued their exponential growth. The 2-3B-G8-MMAE treated animals have no invasion or metastasis, while all the animals of the control group have some metastasis from the xenografted HEPG2 tumor (Figure 7B). All the control-treated animals died within 5 weeks of xenografting, while all 2-3B-G8-MMAE-treated animals survived the same period (Figure 7C). When SCID mice xenografted with HUH7 cells were treated with 2-3B-G8-MMAE, the growth of the tumors was also significantly slowed down (Figure 7D). This contrasted with the exponential nature of tumor growth in the IgG-MMAE control group. However, the inhibition effect of tumor growth by 2-3B-G8-MMAE was largely eliminated after MAN2A1-FER was disrupted. The 2-3B-G8-MMAE–treated animals had no metastasis, while all the control group mice had significant metastasis (Figure 7E). On the other hand, metastasis prevention using 2-3B-G8-MMAE was not significant in HUH7ko mice. All the HUH7 xenografted SCID mice treated by IgG-MMAE died 35 days after the tumor cell implantation, while all the mice treated by 2-3B-G8-MMAE survived the same period (Figure 7F). The survival impact by 2-3B-G8-MMAE, however, was not found for HUH7ko cells xenografted animals.

## DISCUSSION

MAN2A1-FER is one of the frequent fusions in human HCC and is also widely present in other human malignancies.^3^,^4^,^5^,^6^,^7^,^8^,^30^ The surprising finding in this study is the wide presence of the fusion gene in most human cancer cell lines, even though its relative expression levels varied significantly from cell line to cell line. Some cell lines have a high copy number of the breakpoint for the fusion gene, while others have much fewer. Many cell lines appear to contain <1 copy of the known breakpoint per genome, suggesting the presence of other unknown breakpoints for MAN2A1-FER. The underlying mechanism for the generation of the MAN2A1-FER breakpoint remains unclear. This study does not exclude the possibility of genome heterogeneity within a cancer cell line.

The previous report showed that MAN2A1-FER was translocated to the Golgi apparatus.^7^ Since the FER kinase domain is exposed in the lumens of the Golgi, it may have physical contact with the extracellular domains of a variety of transmembrane proteins due to the membrane protein glycosylation process brought by the MAN2A1 domain. In this study, we demonstrated that MAN2A1-FER phosphorylated the extracellular domains of some critical growth factor receptors and EMT regulators and activated the pro-growth and transformation signaling. To our knowledge, this is the first known example of the activation of multiple signaling pathways through ectopic phosphorylation by a pathological chimera protein. Such multipathway activations may have significant implications for liver cancer management and treatment. First, many of the previous observations on growth factor receptor activation in HCC could be the results of MAN2A-FER gene fusion due to the high frequency of these fusion genes in the HCC samples. Second, single pathway blockade through small molecules may not be sufficient to kill off HCC cancer cells that are positive for MAN2A1-FER because of multiple bypasses of the growth signaling.

PDGFR is one of the crucial growth factor receptors essential for the growth and invasion of liver cancers.^31^ Our study indicates that at least 6 tyrosine residues in the extracellular domain of PDGFRA were phosphorylated by MAN2A1-FER. The phosphorylation of these tyrosine residues clearly activated the kinase domain of PDGFRA. Although the physical mechanism leading to the activation of the PDGFR protein kinase remains unclear, the phosphorylation of the extracellular domain may make the extracellular domain of PDGFRA more soluble and acidic. These chemical changes in the protein may help to unfold the domain’s secondary structure. That, in turn, may induce allosteric change and lead to dimerization of PDGFR and the activation of its kinase domain. Indeed, a similar promotion of dimerization and activation of EGFR kinase by MAN2A1-FER through phosphorylation of EGFR tyrosine 88 in the extracellular domain was previously observed.^7^

At the cell level, the exposure of phosphotyrosine epitopes of PDGFRA on the cell surface may make the cancer cells distinctive from the normal hepatocytes immunologically. Since these epitopes are the results of the enzyme activity of MAN2A1-FER kinase, a large number of pathological phosphotyrosine epitopes of PDGFRA may be generated from a few copies of MAN2A1-FER protein. Possibly, many more similar phosphotyrosine epitopes are present in other membrane proteins in MAN2A1-FER–positive cells because the mannosidase and glycoside hydrolase domains of the fusion protein bring many “to be glycosylated” membrane proteins to the proximity of the FER kinase. Thus, developing immunological interventions against these cancer antigens could be an effective means to treat HCC. To our knowledge, the 5 monoclonal antibodies developed in this study are the first examples of immune reagents against tumor-specific antigens that are generated by ectopic phosphorylation. Unlike small molecules for PDGFR, which do not discriminate PDGFR from normal versus cancer cells, these antibodies do not recognize PDGFRA in the benign cells and thus have no impact on benign tissues. As a result, the side effects of these “drugs” may be much less than those of small molecules. This study may serve as proof of principle to show that the antibodies generated against these ectopic epitopes are effective in targeting cancers with high specificity. The immune intervention based on these antibodies may lay a foundation for future diagnosis and treatment for liver cancers that are positive for MAN2A1-FER gene fusion since these antibodies can be humanized to form diagnostic imaging reagents such as flourine^18^-labeled antibodies specific for MAN2A1-FER–positive cancers and to form therapeutic reagents such as drug-conjugated/radio-labeled antibodies, chimera antigen receptor T cells, or cancer cell targeting delivery vehicles in the cancer treatment.

## Supplementary Material

SUPPLEMENTARY MATERIAL

## References

[1] Rumgay H Arnold M Ferlay J Lesi O Cabasag CJ Vignat J. Global burden of primary liver cancer in 2020 and predictions to 2040. J Hepatol. 2022;77:1598–1606. 36208844 10.1016/j.jhep.2022.08.021 PMC9670241

[2] Llovet JM Kelley RK Villanueva A Singal AG Pikarsky E Roayaie S. Hepatocellular carcinoma. Nat Rev Dis Primers. 2021;7:6. 33479224 10.1038/s41572-020-00240-3 PMC13537433

[3] Yu YP Liu S Ren BG Nelson J Jarrard D Brooks JD. Fusion gene detection in prostate cancer samples enhances the prediction of prostate cancer clinical outcomes from radical prostatectomy through machine learning in a multi-institutional analysis. Am J Pathol. 2023;193:392–403. 36681188 10.1016/j.ajpath.2022.12.013 PMC10123524

[4] Yu YP Liu S Nelson J Luo JH. Detection of fusion gene transcripts in the blood samples of prostate cancer patients. Sci Rep. 2021;11:16995. 34417538 10.1038/s41598-021-96528-9 PMC8379170

[5] Yu YP Tsung A Liu S Nalesnick M Geller D Michalopoulos G. Detection of fusion transcripts in the serum samples of patients with hepatocellular carcinoma. Oncotarget. 2019;10:3352–3360. 31164957 PMC6534357

[6] Chen ZH Yu YP Zuo ZH Nelson JB Michalopoulos GK Monga S. Targeting genomic rearrangements in tumor cells through Cas9-mediated insertion of a suicide gene. Nat Biotechnol. 2017;35:543–550. 28459452 10.1038/nbt.3843 PMC5462845

[7] Chen ZH Yu YP Tao J Liu S Tseng G Nalesnik M. MAN2A1-FER fusion gene is expressed by human liver and other tumor types and has oncogenic activity in mice. Gastroenterology. 2017;153:1120–1132. 28245430 10.1053/j.gastro.2016.12.036 PMC5572118

[8] Yu YP Ding Y Chen Z Liu S Michalopoulos A Chen R. Novel fusion transcripts associate with progressive prostate cancer. Am J Pathol. 2014;184:2840–2849. 25238935 10.1016/j.ajpath.2014.06.025 PMC4188871

[9] Luo JH Yu YP Cieply K Lin F Deflavia P Dhir R. Gene expression analysis of prostate cancers. Mol Carcinog. 2002;33:25–35. 11807955 10.1002/mc.10018

[10] Luo JH Ren B Keryanov S Tseng GC Rao UN Monga SP. Transcriptomic and genomic analysis of human hepatocellular carcinomas and hepatoblastomas. Hepatology. 2006;44:1012–1024. 17006932 10.1002/hep.21328 PMC1769554

[11] Ren B Yu G Tseng GC Cieply K Gavel T Nelson J. MCM7 amplification and overexpression are associated with prostate cancer progression. Oncogene. 2006;25:1090–1098. 16247466 10.1038/sj.onc.1209134

[12] Ren B Yu YP Tseng GC Wu C Chen K Rao UN. Analysis of integrin alpha7 mutations in prostate cancer, liver cancer, glioblastoma multiforme, and leiomyosarcoma. J Natl Cancer Inst. 2007;99:868–880. 17551147 10.1093/jnci/djk199

[13] Yu YP Yu G Tseng G Cieply K Nelson J Defrances M. Glutathione peroxidase 3, deleted or methylated in prostate cancer, suppresses prostate cancer growth and metastasis. Cancer Res. 2007;67:8043–8050. 17804715 10.1158/0008-5472.CAN-07-0648

[14] Nalesnik MA Tseng G Ding Y Xiang GS Zheng ZL Yu Y. Gene deletions and amplifications in human hepatocellular carcinomas: Correlation with hepatocyte growth regulation. Am J Pathol. 2012;180:1495–1508. 22326833 10.1016/j.ajpath.2011.12.021 PMC3657620

[15] Yu YP Song C Tseng G Ren BG Laframboise W Michalopoulos G. Genome abnormalities precede prostate cancer and predict clinical relapse. Am J Pathol. 2012;180:2240–2248. 22569189 10.1016/j.ajpath.2012.03.008 PMC3385611

[16] Han YC Zheng ZL Zuo ZH Yu YP Chen R Tseng GC. Metallothionein 1 h tumour suppressor activity in prostate cancer is mediated by euchromatin methyltransferase 1. J Pathol. 2013;230:184–193. 23355073 10.1002/path.4169 PMC4080639

[17] Yu YP Ding Y Chen R Liao SG Ren BG Michalopoulos A. Whole-genome methylation sequencing reveals distinct impact of differential methylations on gene transcription in prostate cancer. Am J Pathol. 2013;183:1960–1970. 24113458 10.1016/j.ajpath.2013.08.018 PMC5745540

[18] Cunningham D Parajuli KR Zhang C Wang G Mei J Zhang Q. Monomethyl auristatin E phosphate inhibits human prostate cancer growth. Prostate. 2016;76:1420–1430. 27325602 10.1002/pros.23226 PMC5033698

[19] Scheinberg IH. Scatchard plots. Science. 1982;215:312–313. 17784358 10.1126/science.215.4530.312

[20] Luo JH Liu S Tao J Ren BG Luo K Chen ZH. Pten-NOLC1 fusion promotes cancers involving MET and EGFR signalings. Oncogene. 2021;40:1064–1076. 33323972 10.1038/s41388-020-01582-8 PMC7880894

[21] He DM Ren BG Liu S Tan LZ Cieply K Tseng G. Oncogenic activity of amplified miniature chromosome maintenance 8 in human malignancies. Oncogene. 2017;36:3629–3639. 28481876 10.1038/onc.2017.123 PMC5481462

[22] Zhang XY Tang LZ Ren BG Yu YP Nelson J Michalopoulos G. Interaction of MCM7 and RACK1 for activation of MCM7 and cell growth. Am J Pathol. 2013;182:796–805. 23313748 10.1016/j.ajpath.2012.11.020 PMC3586685

[23] Wang H Luo K Tan LZ Ren BG Gu LQ Michalopoulos G. p53-induced gene 3 mediates cell death induced by glutathione peroxidase 3. J Biol Chem. 2012;287:16890–16902. 22461624 10.1074/jbc.M111.322636 PMC3351337

[24] Zhu ZH Yu YP Shi YK Nelson JB Luo JH. CSR1 induces cell death through inactivation of CPSF3. Oncogene. 2009;28:41–51. 18806823 10.1038/onc.2008.359 PMC2918272

[25] Zuo ZH Yu YP Ren BG Liu S Nelson J Wang Z. Oncogenic activity of solute carrier family 45 member 2 and alpha-methylacyl-coenzyme A racemase gene fusion is mediated by mitogen-activated protein kinase. Hepatol Commun. 2022;6:209–222. 34505419 10.1002/hep4.1724 PMC8710797

[26] Han YC Yu YP Nelson J Wu C Wang H Michalopoulos GK. Interaction of integrin-linked kinase and miniature chromosome maintenance 7-mediating integrin {alpha}7 induced cell growth suppression. Cancer Res. 2010;70:4375–4384. 20460506 10.1158/0008-5472.CAN-09-4403 PMC3097463

[27] Shi YK Yu YP Tseng GC Luo JH. Inhibition of prostate cancer growth and metastasis using small interference RNA specific for minichromosome complex maintenance component 7. Cancer Gene Ther. 2010;17:694–699. 20539323 10.1038/cgt.2010.25 PMC4041301

[28] Yu G Tseng GC Yu YP Gavel T Nelson J Wells A. CSR1 suppresses tumor growth and metastasis of prostate cancer. Am J Pathol. 2006;168:597–607. 16436673 10.2353/ajpath.2006.050620 PMC1606498

[29] Jing L Liu L Yu YP Dhir R Acquafondada M Landsittel D. Expression of myopodin induces suppression of tumor growth and metastasis. Am J Pathol. 2004;164:1799–1806. 15111326 10.1016/S0002-9440(10)63738-8 PMC1615646

[30] Luo JH Liu S Zuo ZH Chen R Tseng GC Yu YP. Discovery and classification of fusion transcripts in prostate cancer and normal prostate tissue. Am J Pathol. 2015;185:1834–45. 25963990 10.1016/j.ajpath.2015.03.008 PMC4483459

[31] Wei T Zhang LN Lv Y Ma XY Zhi L Liu C. Overexpression of platelet-derived growth factor receptor alpha promotes tumor progression and indicates poor prognosis in hepatocellular carcinoma. Oncotarget. 2014;5:10307–10317. 25333264 10.18632/oncotarget.2537 PMC4279374

