# Supplementary material for: Immuno-targeting the ectopic phosphorylation sites of PDGFRA generated by MAN2A1-FER fusion in HCC
Source: Hepatol Commun. 2024 Jul 31;8(8):e0511. doi: 10.1097/HC9.0000000000000511 (PMC12333812; doi:10.1097/HC9.0000000000000511)
Supplement: SUPPLEMENTARY MATERIAL [file hc9-8-e0511-s001.pdf]

**Supplemental table 1 Primers for vector construction**

| Vector | Primer pair | cDNA |
|---|---|---|
| pET28-HisTAG-ΔPDGFRA$^{aa1-528}$ | AAGCTTGAATTCATGGGGACTTCCCATCCGGCGT/<br>TCTAGAGCGGCCGCAGCCACCGTGAGTTCAGAACG | PDGFRA |
| pCMV13-HisTAG-ΔPDGFRA$^{aa1-528}$ | AAGCTTGAATTCCATCATCATCATCATCACATGGGGACTTCCCATCCGGCGT/<br>TCTAGAGCGGCCGCAGCCACCGTGAGTTCAGAACG | PDGFRA |
| pET28-HisTAG-ΔMET$^{aa1-646}$ | AAGCTTGAATTCATGAAGGCCCCCGCTGTGCTT/<br>TCTAGAGCGGCCGCTGTCCCGTGGCCATTTGAAAT | MET |
| pCMV13-HisTAG-ΔMET$^{aa1-646}$ | AAGCTTGAATTCCATCATCATCATCATCACATGAAGGCCCCCGCTGTGCTT/<br>TCTAGAGCGGCCGCTGTCCCGTGGCCATTTGAAAT | MET |
| pET28-HisTAG-ΔAXL$^{aa1-451}$ | AAGCTTGAATTCATGGCGTGGCGGTGCCCCAGG/<br>TCTAGAGCGGCCGCCCACCAGGGCCACGAGAAGGC | AXL |
| pCMV13-HisTAG-ΔAXL$^{aa1-451}$ | AAGCTTGAATTCCATCATCATCATCATCACATGGCGTGGCGGTGCCCCAGG/<br>TCTAGAGCGGCCGCCCACCAGGGCCACGAGAAGGC | AXL |
| pET28-HisTAG-ΔCDH2$^{aa1-724}$ | AAGCTTGAATTCATGTGCCGGATAGCGGGAGCG/<br>TCTAGAGCGGCCGCGGCACCGGTGCCAAGCCCCGC | CDH2 |
| pCMV13-HisTAG-ΔCDH2$^{aa1-724}$ | AAGCTTGAATTCCATCATCATCATCATCACATGTGCCGGATAGCGGGAGCG/<br>TCTAGAGCGGCCGCGGCACCGGTGCCAAGCCCCGC | CDH2 |

**Supplemental table 2. Peptide sequences corresponding to extracellular domains of PDGFR, MET, AXL and CDH2**

| Gene | Peptide No. | Tyrosine position | Sequence |
|---|---|---|---|
| PDGFR | 1 | Y59 | CFGESEVSWQYPMSEEESSDV |
| | 2 | Y98/Y101-2 | SSASAAHTGLYTCYYNHTQTE |
| | 3 | Y118/Y120 | EENELEGRHIYIYVPDPDVAF |
| | 4 | Y136 | VAFVPLGMTDYLVIVEDDDSA |
| | 5 | Y172 | HNSEGVVPASYDSRQGFNGTF |
| | 6 | Y187 | GFNGTFTVGPYICEATVKGKK |
| | 7 | Y206 | KKFQTIPFNVYALKATSELDL |
| | 8 | Y225 | DLEMEALKTVYKSGETIVVTC |
| | 9 | Y249 | NNEVVDLQWTYPGEVKGKGIT |
| | 10 | Y273 | EIKVPSIKLVYTLTVPEATVK |
| | 11 | Y288 | PEATVKDSGDYECAARQATRE |
| | 12 | Y342 | VKHFVVEVRAYPPPRISWLKN |
| | 13 | Y375 | TDVEKIQEIRYRSKLKLIRAK |
| | 14 | Y391 | IRAKEEDSGHYTIVAQNEDAV |
| | 15 | Y405 | VAQNEDAVKSYTFELLTQVPS |
| MET | 1 | Y41 | AKSEMNVNMKYQLPNFTAETP |
| | 2 | Y69/Y71 | EHHIFLGATNYIYVLNEEDLQ |
| | 3 | Y84 | NEEDLQKVAEYKTGPVLEHPD |
| | 4 | Y125/Y126 | NINMALVVDTYYDDQLISCGS |
| | 5 | Y205 | FFVGNTINSSYFPDHPLHSIS |
| | 6 | Y234 | DGFMFLTDQSYIDVLPEFRDS |
| | 7 | Y245/Y249 | IDVLPEFRDSYPIKYVHAFES |
| | 8 | Y260 | VHAFESNNFIYFLTVQRETLD |
| | 9 | Y291 | FCSINSGLHSYMEMPLECILT |
| | 10 | Y321 | KEVFNILQAAYVSKPGAQLAR |
| | 11 | Y369 | RSAMCAFPIKYVNDFFNKIVN |
| | 12 | Y390 | KNNVRCLQHFYGPNHEHCFNR |
| | 13 | Y416 | SSGCEARRDEYRTEFTTALQR |
| | 14 | Y501 | IVEHTLNQNGYTLVITGKKIT |
| | 15 | Y566 | WTQQICLPAIYKVFPNSAPLE |
| AXL | 1 | Y115 | RITSLQLSDTGQYQCLVFLGHQTFV |
| | 2 | Y132 | QCLVFLGHQTFVSQPGYVGLEGLP |
| | 3 | Y140 | VGLEGLPYFLEEPEDRTVAANTPFN |
| | 4 | Y253 | LEVAWTPGLSGIYPLTHCTLQAVLS |
| | 5 | Y367/Y371 | RAPLQGTLLGYRLAYQGQDTPEVLM |
| | 6 | Y409 | GSVSNLTVCVAAYTAAGDGPWSLPV |
| CDH2 | 1 | Y40 | LCKTGFPEDVYSAVLSKDVHE |
| | 2 | Y70 | SNCNGKRKVQYESSEPADFKV |
| | 3 | Y87 | DFKVDEDGMVYAVRSFPLSSE |
| | 4 | Y104 | LSSEHAKFLIYAQDKETQEKW |
| | 5 | Y194 | SDRDKNLSLRYSVTGPGADQP |
| | 6 | Y284 | TVPEGSKPGTYVMTVTAIDAD |
| | 7 | Y305 | DPNALNGMLRYRIVSQAPSTP |
| | 8 | Y344 | AGLDREKVQQYTLIIQATDME |
| | 9 | Y359 | QATDMEGNPTYGLSNTATAVI |
| | 10 | Y387 | NPPEFTAMTFYGEVPENRVDI |
| | 11 | Y419 | QPHTPAWNAVYRISGGDPTGR |
| | 12 | Y495 | VTVIDVNENPYFAPNPKIIRQ |
| | 13 | Y526/Y533 | TTFTAQDPDRYMQQNIRYTKL |
| | 14 | Y526/Y533 | PDRYMQQNIRYTKLSDPANWL |
| | 15 | Y570 | RESPNVKNNIYNATFLASDNG |
| | 16 | Y593 | PMSGTGTLQIYLLDINDNAPQ |
| | 17 | Y627 | PNSINITALDYDIDPNAGPFA |
| | 18 | Y675 | LKIKFLEAGIYEVPIIITDSG |

**Supplemental table 3. Tyrosine phosphorylated peptides and their unphosphorylated controls corresponding to the extracellular domain of PDGFRA**

| Peptide No. | Position of Y phosphorylation | Peptide sequence |
|---|---|---|
| 1 | pY118/pY120 | QTEENELEGRHIpYIpYVPDPDVAFVP |
| 2 | pY118 | QTEENELEGRHIpYIYVPDPDVAFVP |
| 3 | pY120 | QTEENELEGRHIYIpYVPDPDVAFVP |
| 4 | pY288 | PEATVKDSGDpYECAARQATREVK |
| 5 | Y288 | PEATVKDSGDYECAARQATREVK |
| 6 | pY342 | VKHFVVEVRApYPPPRISWLKN |
| 7 | Y342 | VKHFVVEVRAYPPPRISWLKN |
| 8 | Y375 | TDVEKIQEIRYRSKLKLIRAK |
| 9 | pY375 | TDVEKIQEIRpYRSKLKLIRAK |
| 10 | pY391 | IRAKEEDSGHpYTIVAQNEDAV |
| 11 | Y391 | IRAKEEDSGHYTIVAQNEDAV |
| 12 | Y118/Y119 | QTEENELEGRHIYIYVPDPDVAFVP |
