# Supplementary material for: Immuno-targeting the ectopic phosphorylation sites of PDGFRA generated by MAN2A1-FER fusion in HCC
Source: Hepatol Commun. 2024 Jul 31;8(8):e0511. doi: 10.1097/HC9.0000000000000511 (PMC12333812; doi:10.1097/HC9.0000000000000511)

Supplemental figure 1. MAN2A1-FER fusion expression and breakpoints in human cancer cell lines.(A) Images of Taqman qRT-PCR of MAN2A1-FER and β-actin on mRNA of human cancer cell lines. The positions of the TaqMan probe and primers were indicated. (B) Images of Chromogram of Sanger's sequencing on the fusion juncture of MAN2A1-FER mRNA. The position of the fusion juncture was indicated. (C) Images of Taqman qPCR of MAN2A1-FER on genome DNA of human cancer cell lines. The positions of the TaqMan probe and primers were indicated. (D) Images of Chromogram of Sanger's sequencing on the genome breakpoint juncture of MAN2A1-FER. The position of the fusion juncture was indicated.

(A) Images of Taqman qRT-PCR of MAN2A1-FER and β-actin on mRNA of human cancer cell lines.

Supplemental figure 1A

MAN2A1-FER mRNA Taqman qRT-PCR experiment #1

MAN2A1-FER

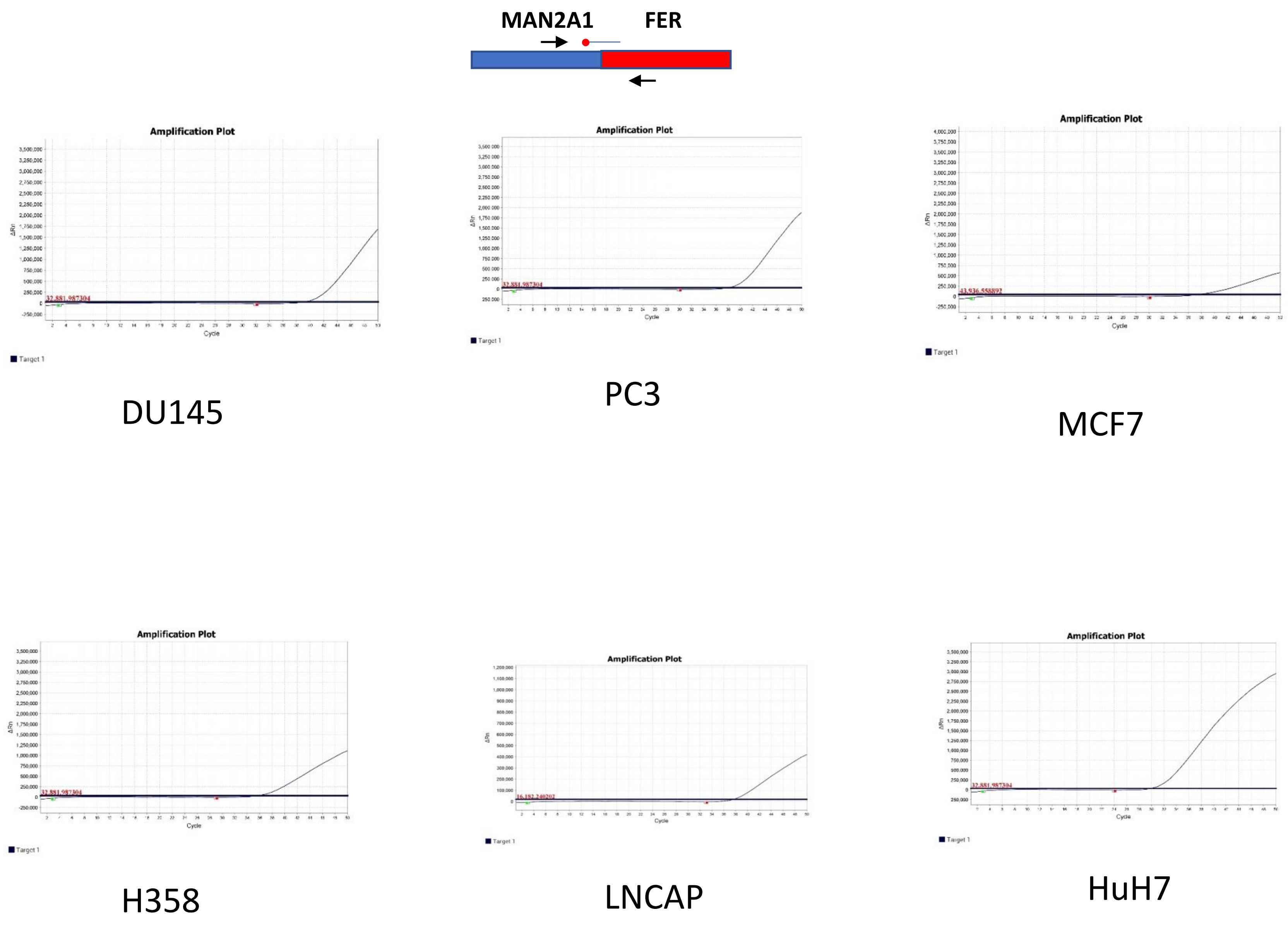

Supplemental figure 1A

MAN2A1-FER mRNA Taqman qRT-PCR experiment #1

MAN2A1-FER

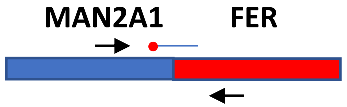

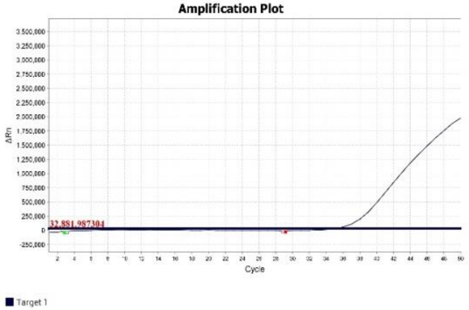

H1299

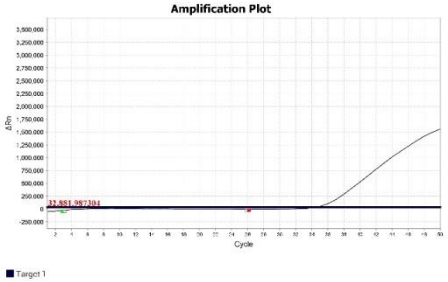

HCT8

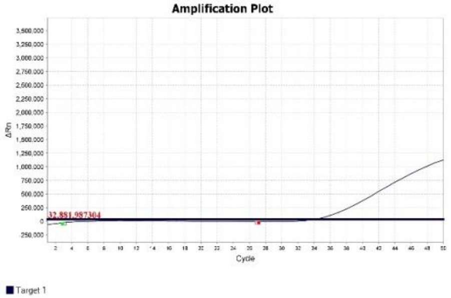

LN229

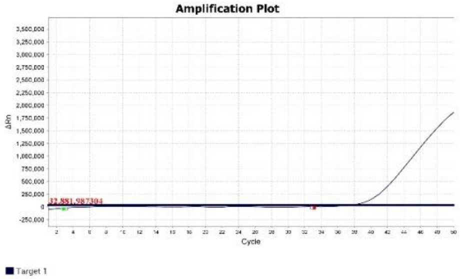

U138

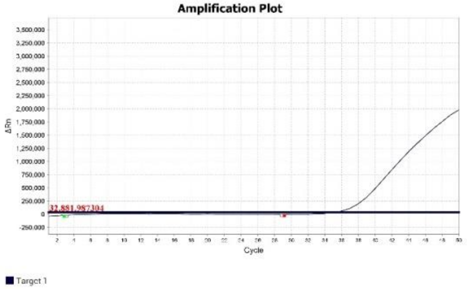

SNU387

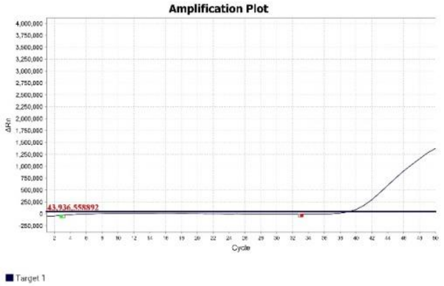

SNU449

Supplemental figure 1A

MAN2A1-FER mRNA Taqman qRT-PCR experiment #1

MAN2A1-FER

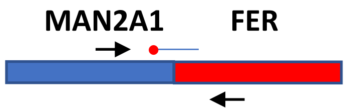

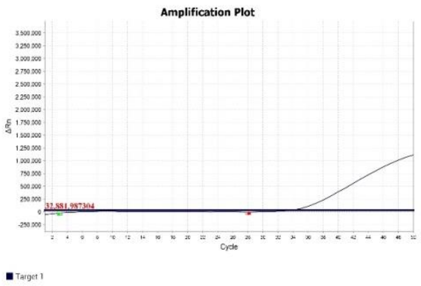

293WT

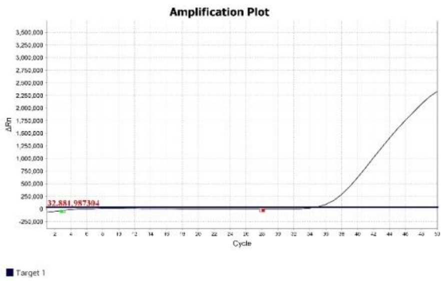

SNU475

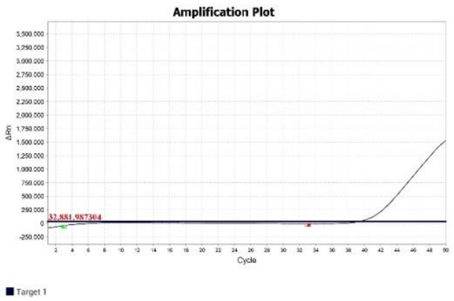

SNU182

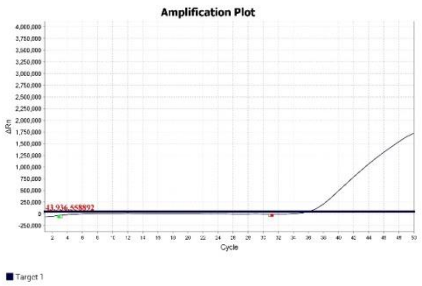

H2198

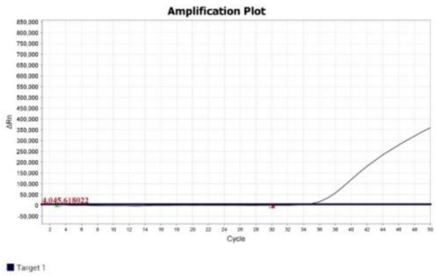

T98G

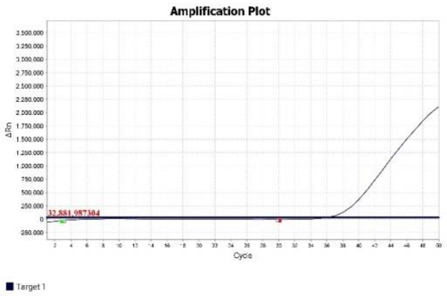

MB231

Supplemental figure 1A

MAN2A1-FER mRNA Taqman qRT-PCR experiment #1

MAN2A1-FER

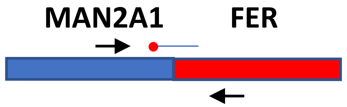

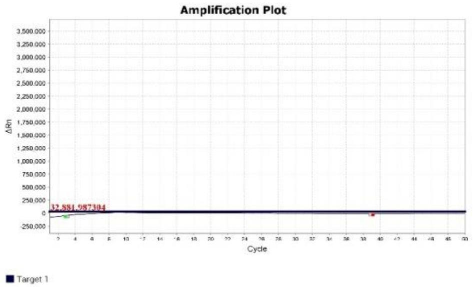

HeP3B

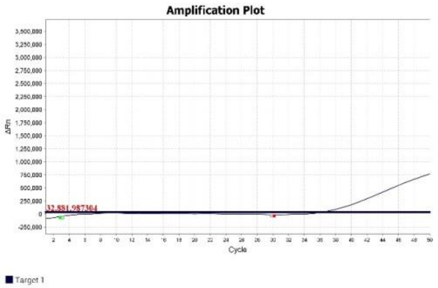

MDA-MB330

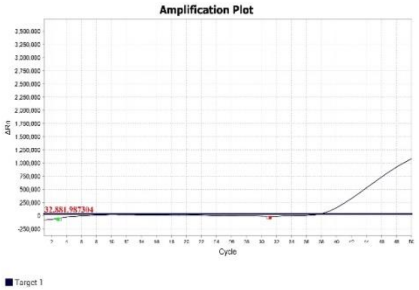

H522

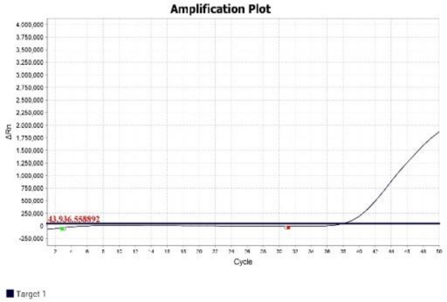

U118

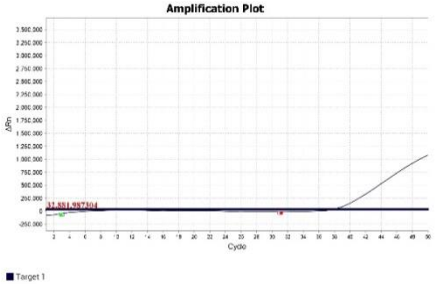

HepG2

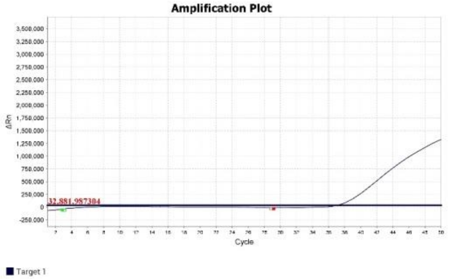

HCT15

Supplemental figure 1A

MAN2A1-FER mRNA Taqman qRT-PCR experiment #2

MAN2A1-FER

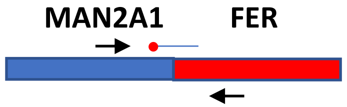

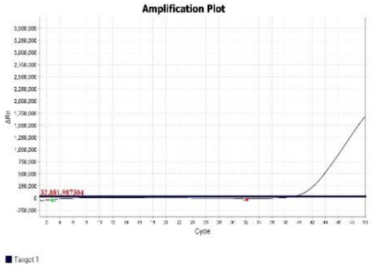

DU145

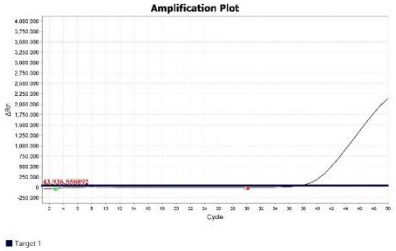

PC3

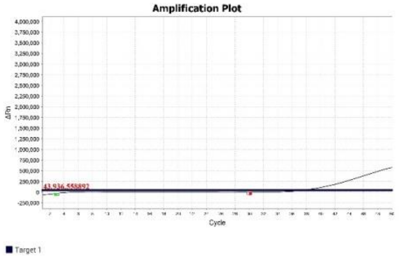

MCF7

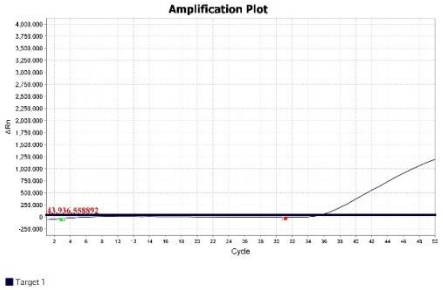

H358

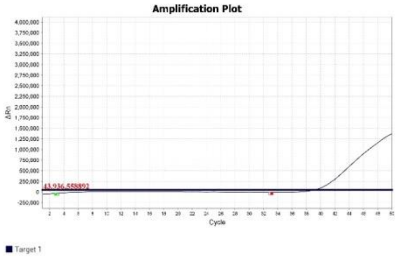

LNCAP

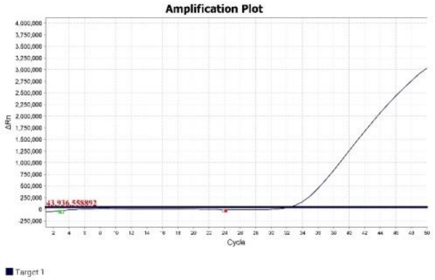

HuH7

Supplemental figure 1A

MAN2A1-FER mRNA Taqman qRT-PCR experiment #2

MAN2A1-FER

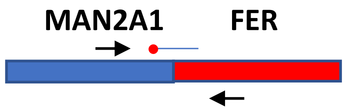

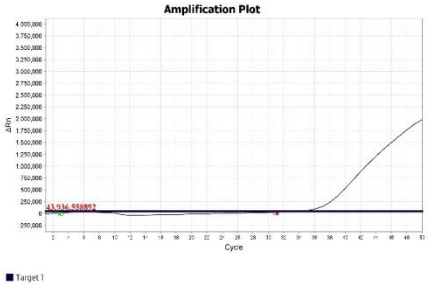

H1299

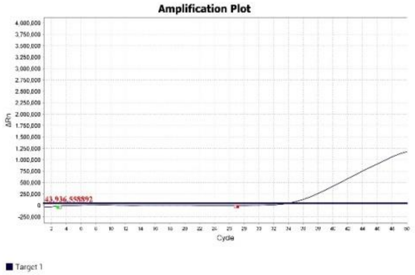

HCT8

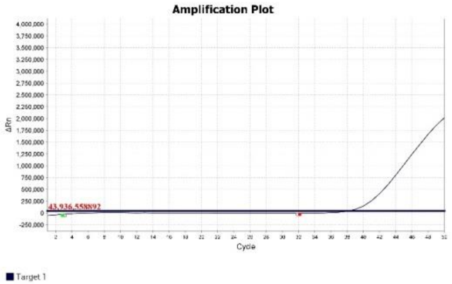

LN229

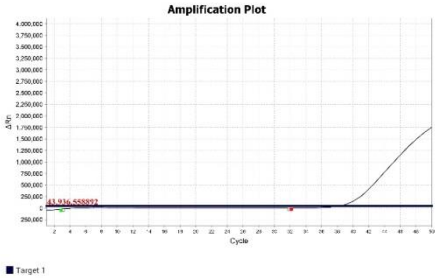

U138

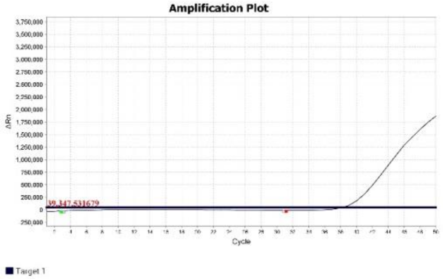

SNU387

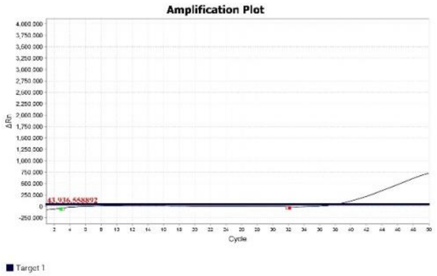

SNU449

Supplemental figure 1A

MAN2A1-FER mRNA Taqman qRT-PCR experiment #2

MAN2A1-FER

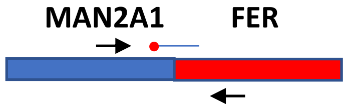

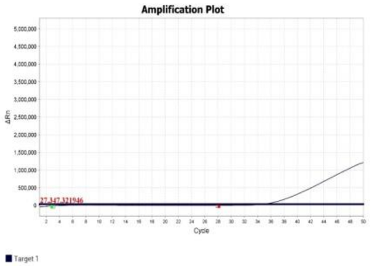

293WT

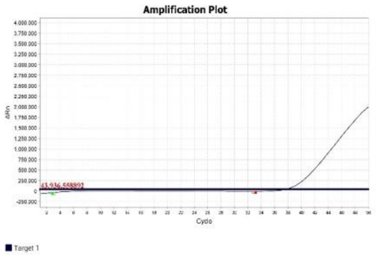

SNU475

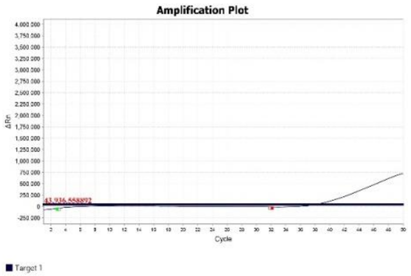

SNU182

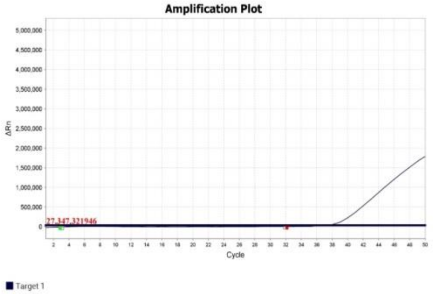

H2198

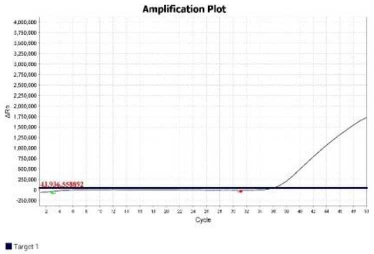

T98G

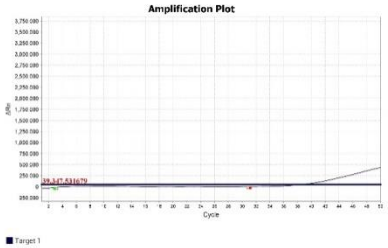

MB231

Supplemental figure 1A

MAN2A1-FER mRNA Taqman qRT-PCR experiment #2

MAN2A1-FER

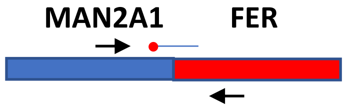

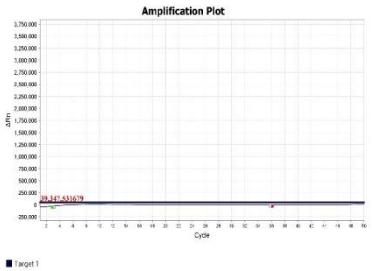

HeP3B

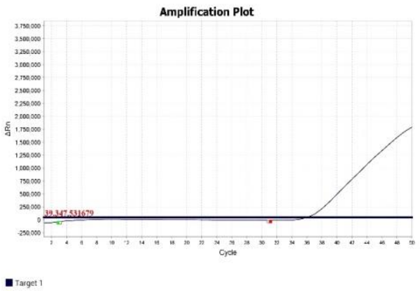

MDA-MB330

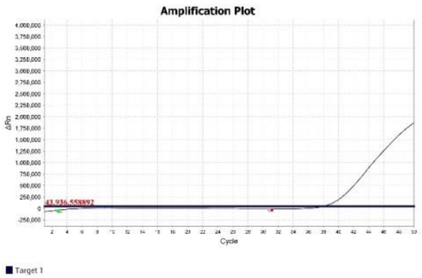

H522

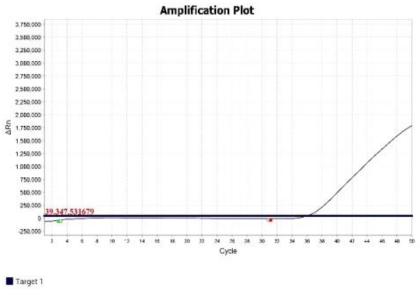

U118

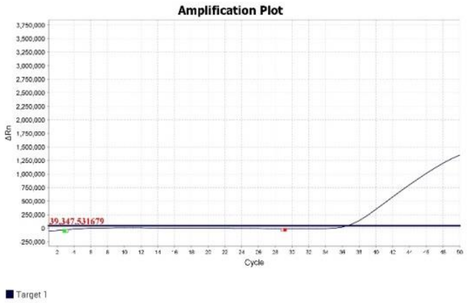

HepG2

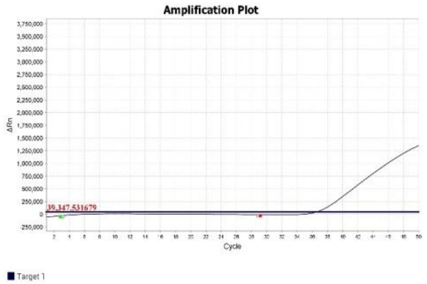

HCT15

Supplemental figure 1A

Beta-actin mRNA Taqman qRT-PCR experiment #1

β-actin

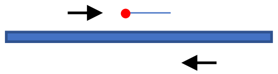

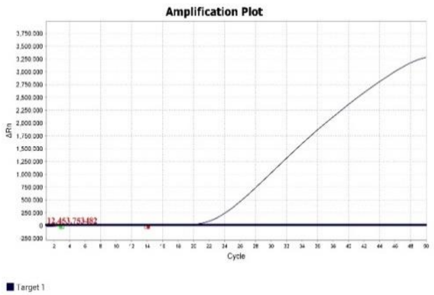

DU145

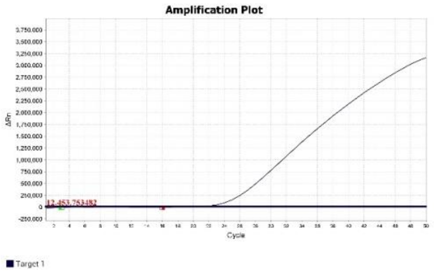

PC3

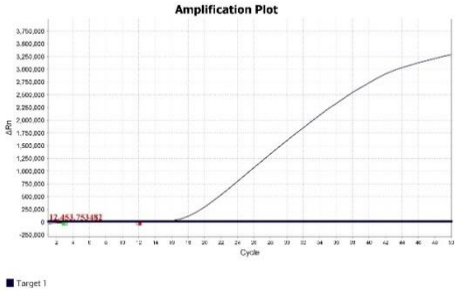

MCF7

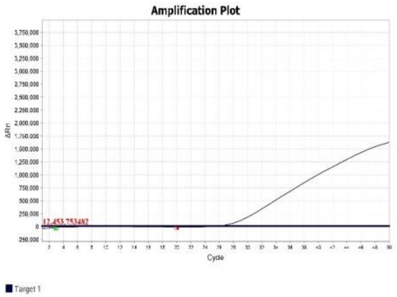

H358

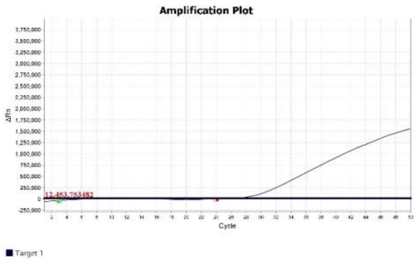

LNCAP

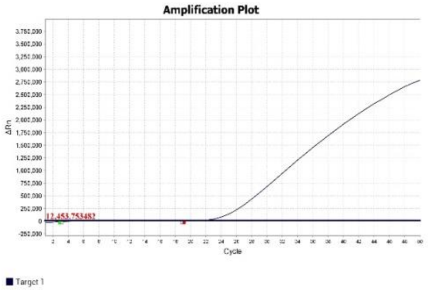

HuH7

Supplemental figure 1A

Beta-actin mRNA Taqman qRT-PCR experiment #1

β-actin

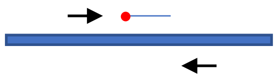

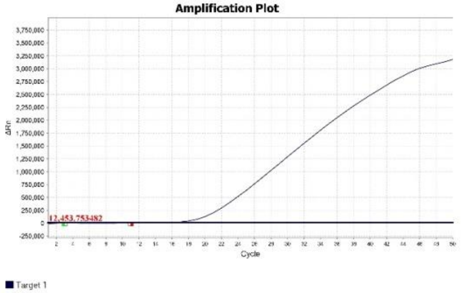

H1299

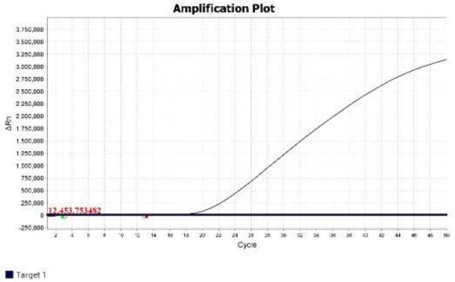

HCT8

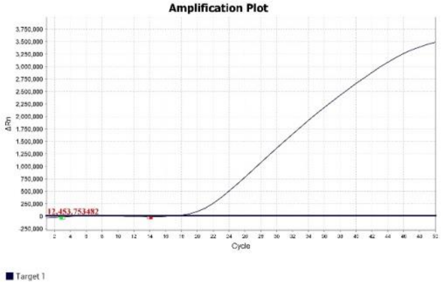

LN229

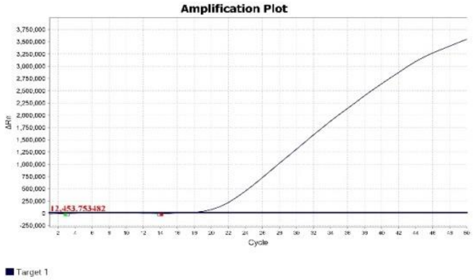

U138

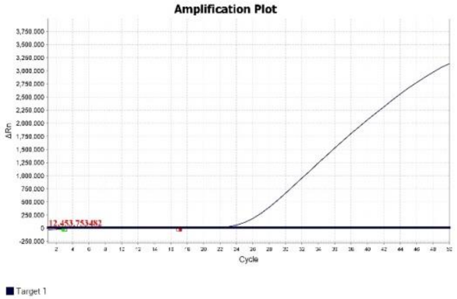

SNU387

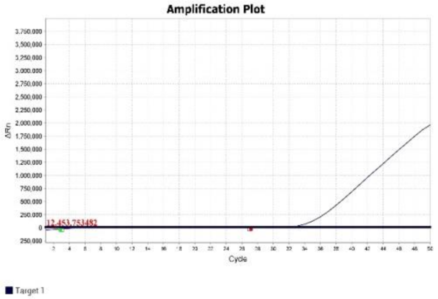

SNU449

Supplemental figure 1A

Beta-actin mRNA Taqman qRT-PCR experiment #1

β-actin

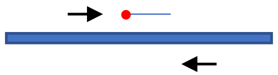

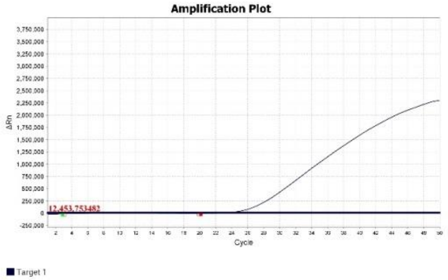

293WT

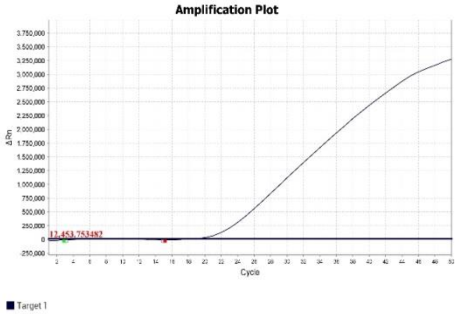

SNU475

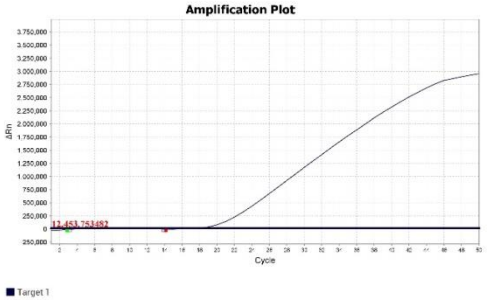

SNU182

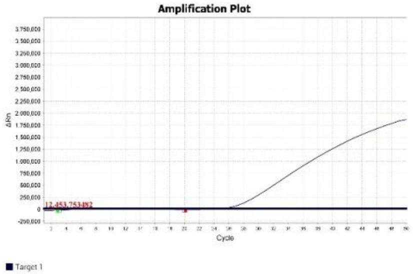

H2198

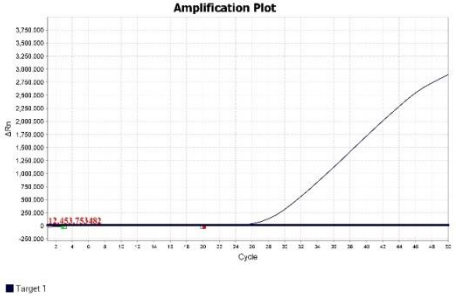

T98G

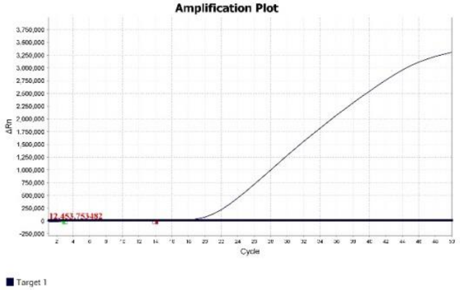

MB231

Supplemental figure 1A

Beta-actin mRNA Taqman qRT-PCR experiment #1

β-actin

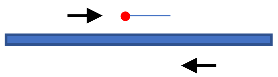

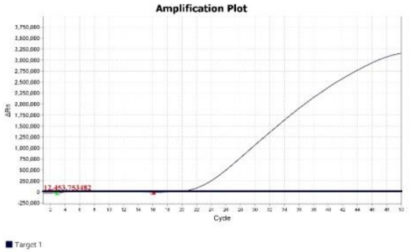

HeP3B

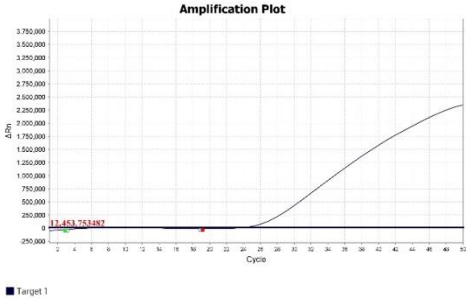

MDA-MB330

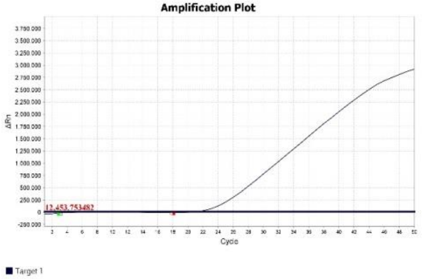

H522

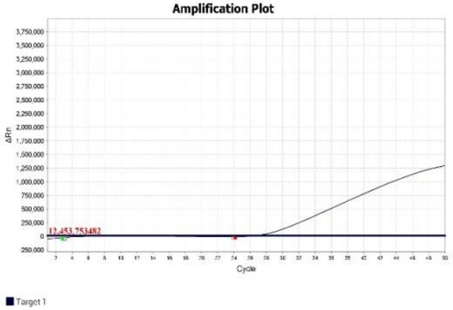

U118

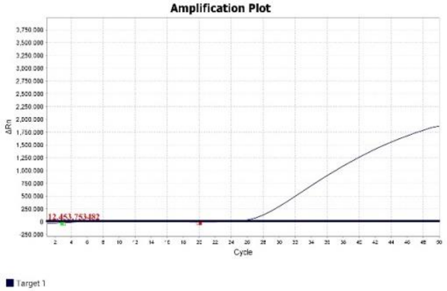

HepG2

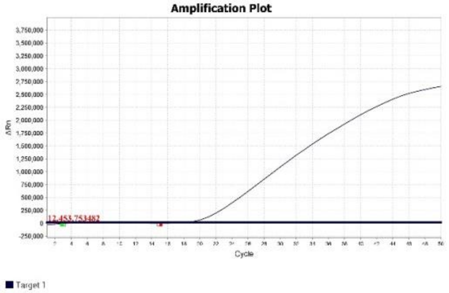

HCT15

Supplemental figure 1A

Beta-actin mRNA Taqman qRT-PCR experiment #2

β-actin

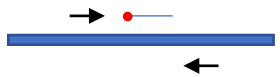

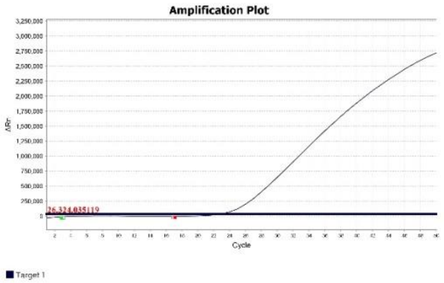

DU145

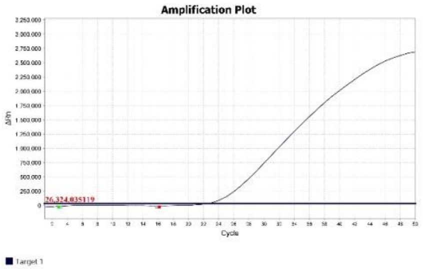

PC3

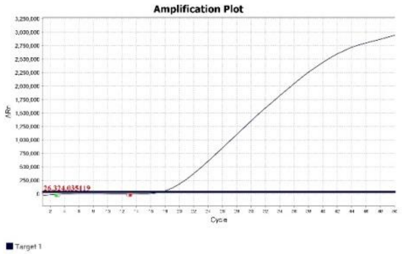

MCF7

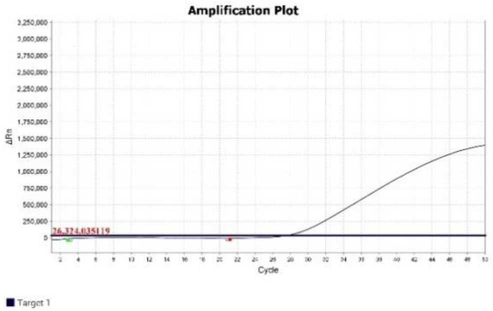

H358

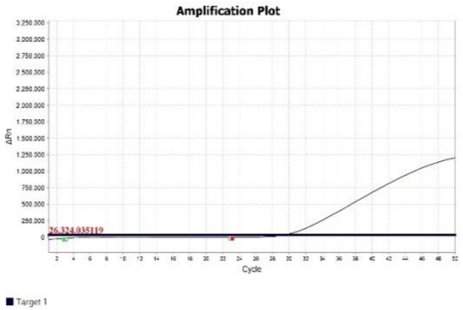

LNCAP

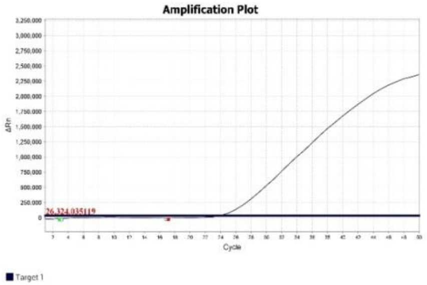

HuH7

Supplemental figure 1A

Beta-actin mRNA Taqman qRT-PCR experiment #2

β-actin

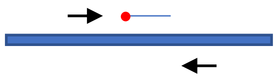

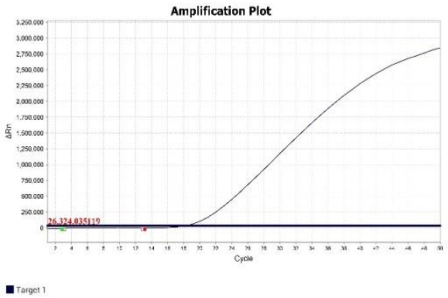

H1299

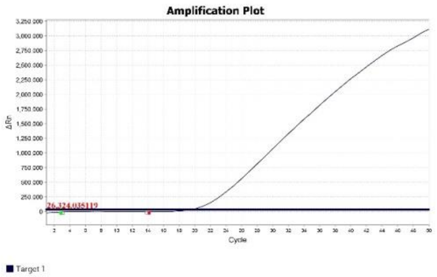

HCT8

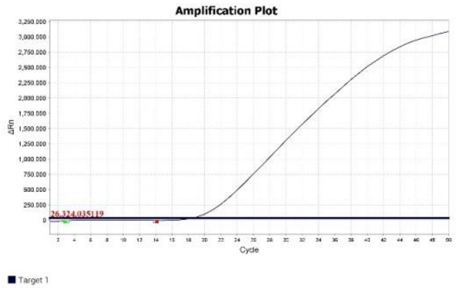

LN229

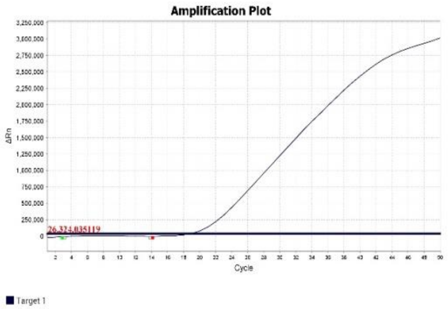

U138

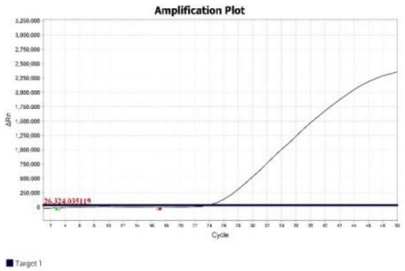

SNU387

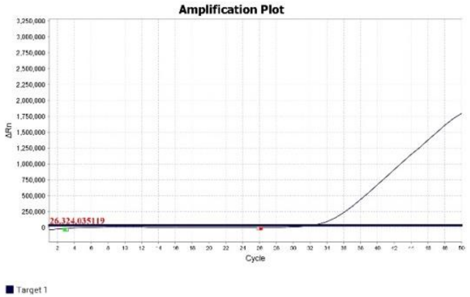

SNU449

Supplemental figure 1A

Beta-actin mRNA Taqman qRT-PCR experiment #2

β-actin

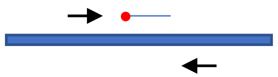

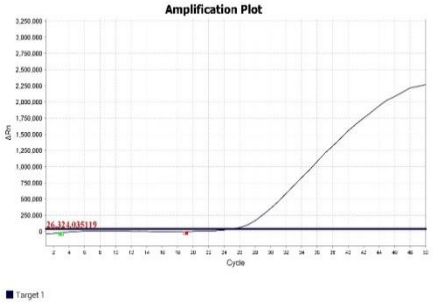

293WT

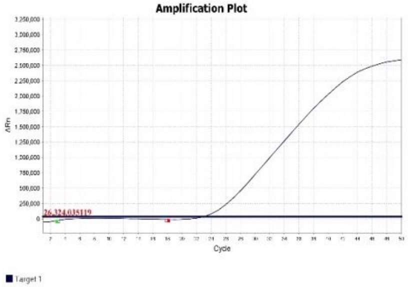

SNU475

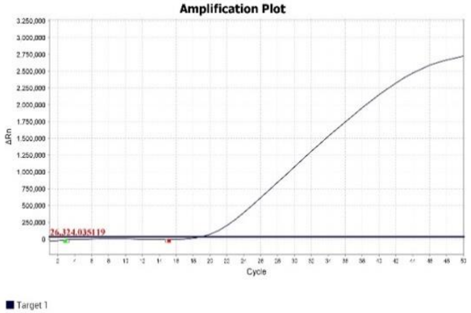

SNU182

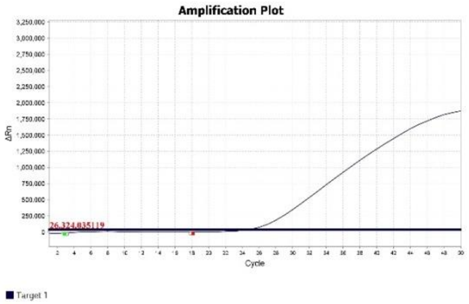

H2198

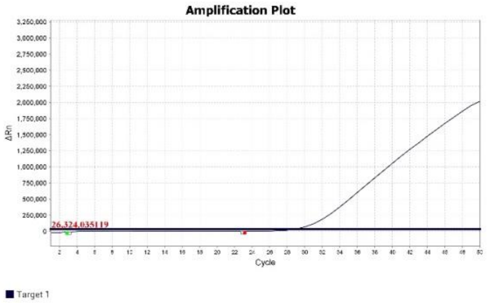

T98G

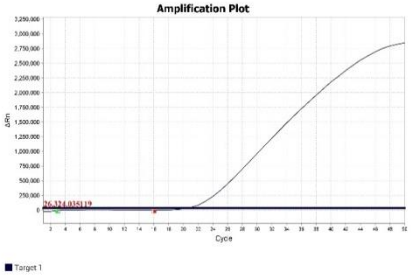

MB231

Supplemental figure 1A

Beta-actin mRNA Taqman qRT-PCR experiment #2

β-actin

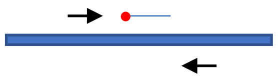

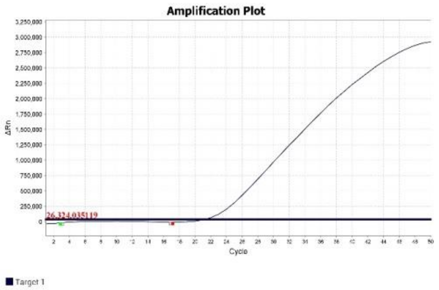

HeP3B

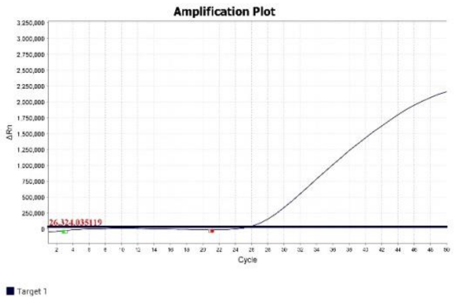

MDA-MB330

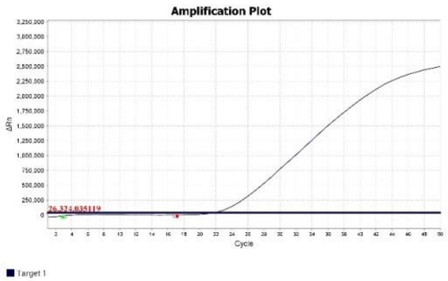

H522

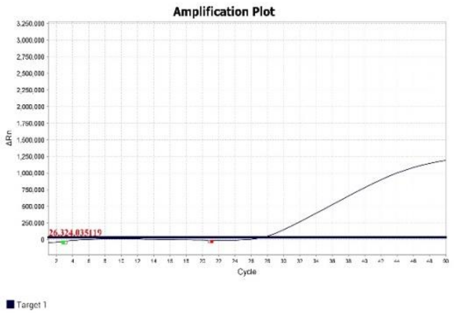

U118

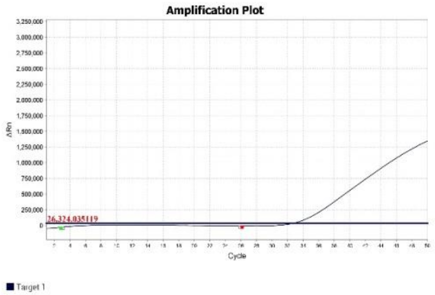

HepG2

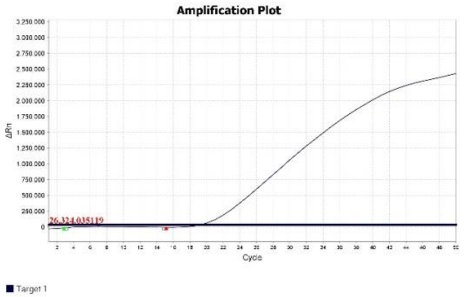

HCT15

Supplemental figure 1B

(B) Images of Chromogram of Sanger's sequencing on the fusion juncture of MAN2A1-FER mRNA.

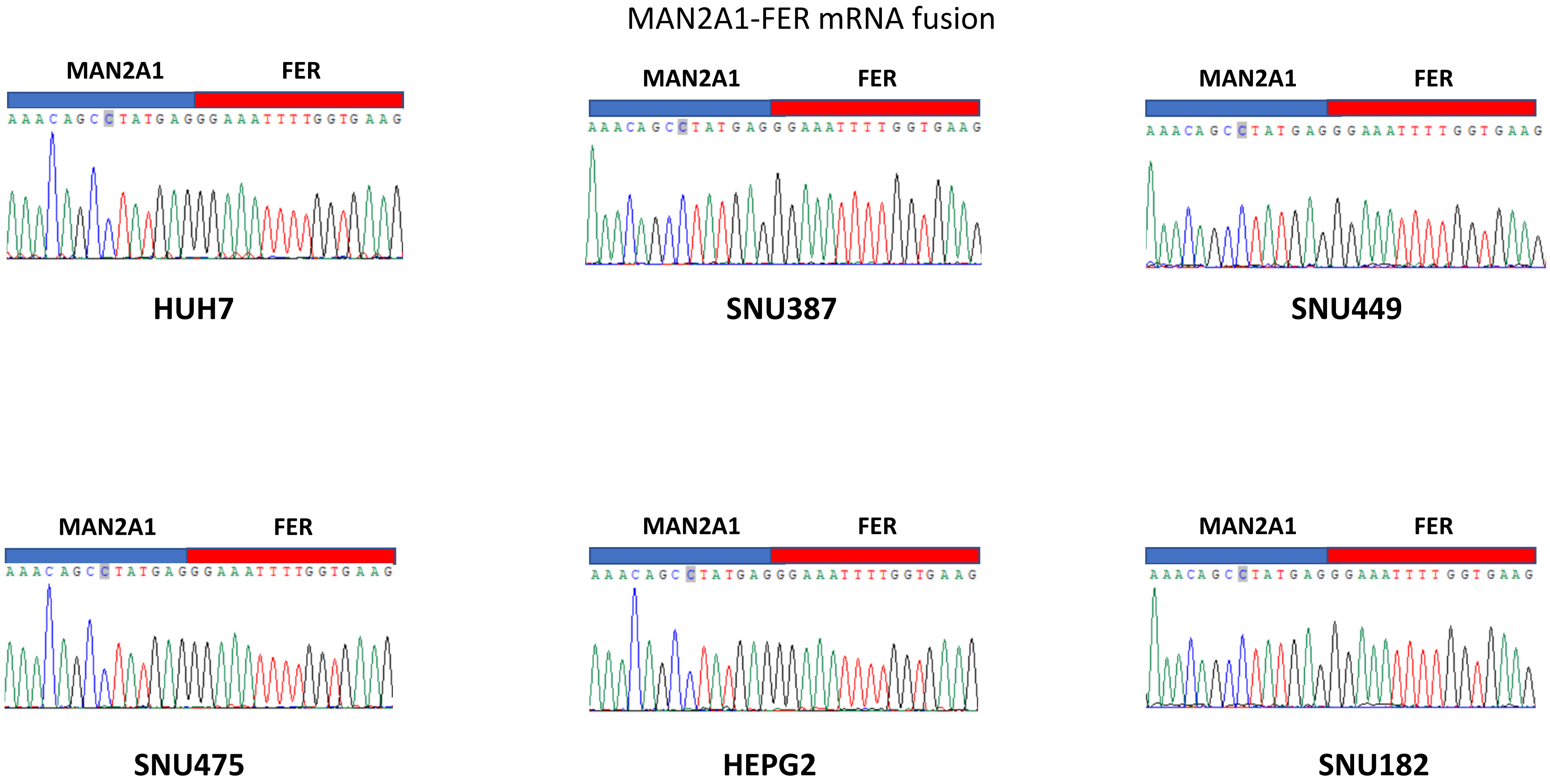

Supplemental figure 1B

MAN2A1-FER mRNA fusion

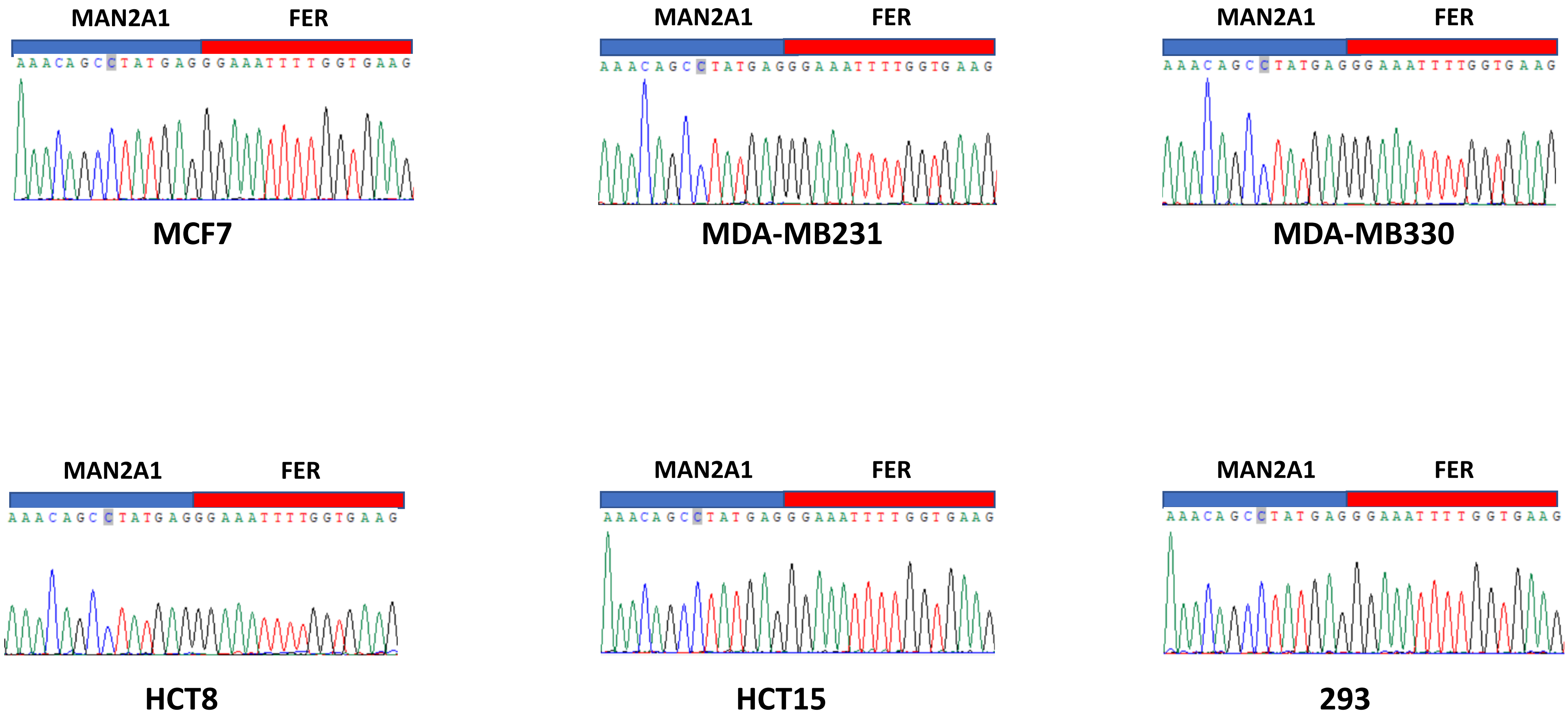

Supplemental figure 1B

MAN2A1-FER mRNA fusion

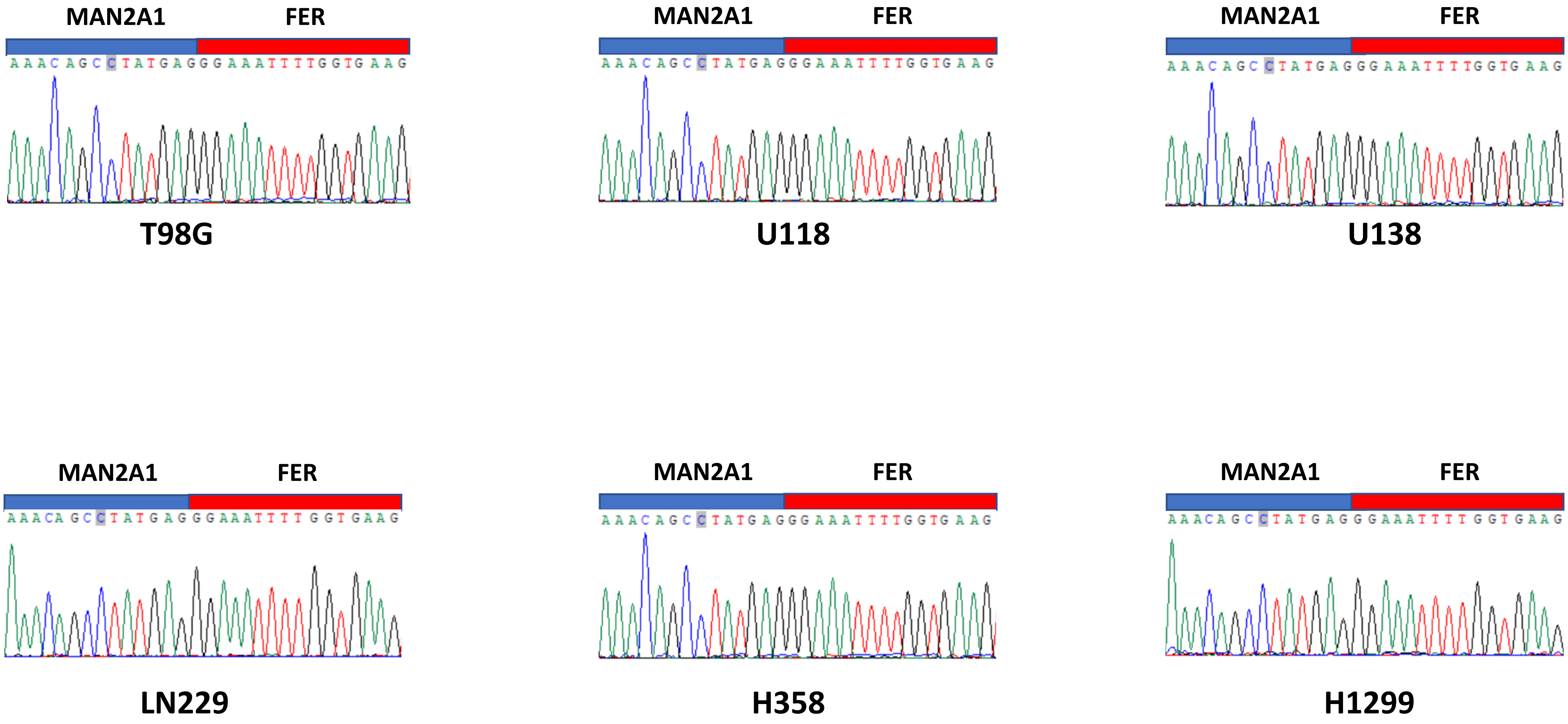

Supplemental figure 1B

MAN2A1-FER mRNA fusion

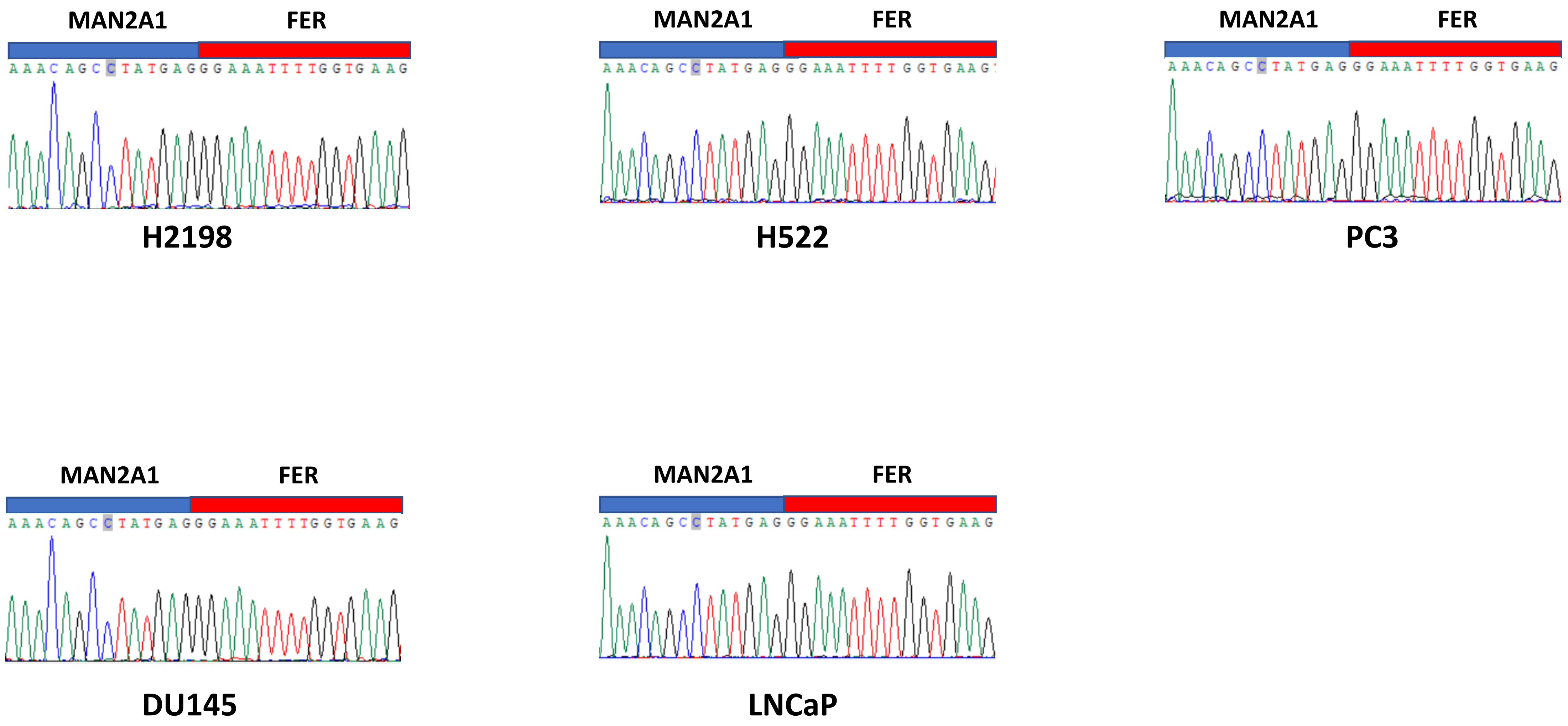

Supplemental figure 1C

(C) Images of Taqman qPCR of MAN2A1-FER on genome DNA of human cancer cell lines.

MAN2A1-FER breakpoint Taqman qPCR experiment #1

MAN2A1-FER Breakpoint

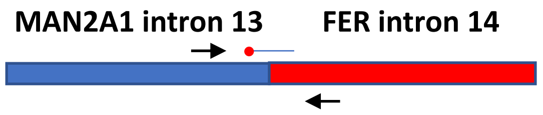

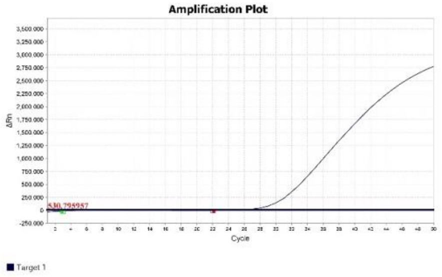

H358

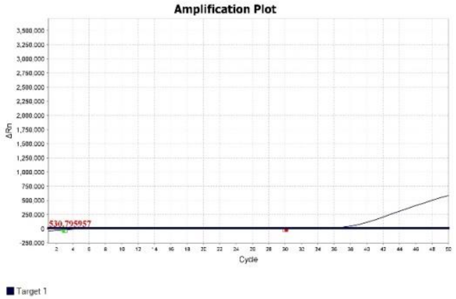

HCT8

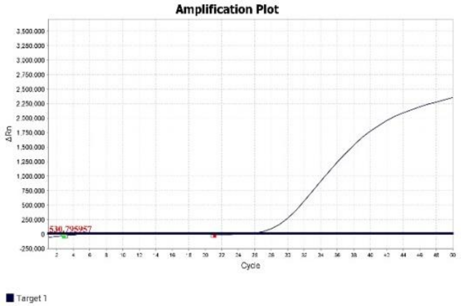

SNU475

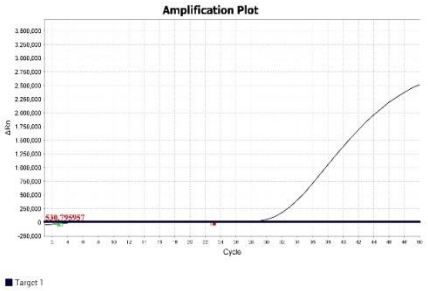

MB231

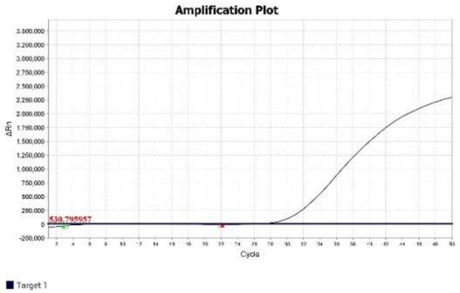

HepG2

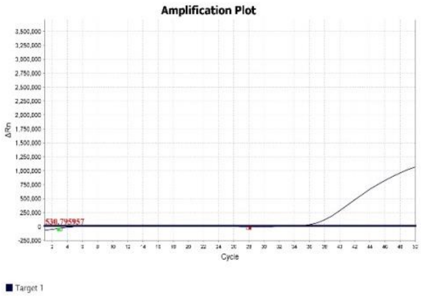

DU145

Supplemental figure 1C

MAN2A1-FER breakpoint Taqman qPCR experiment #1

MAN2A1-FER Breakpoint

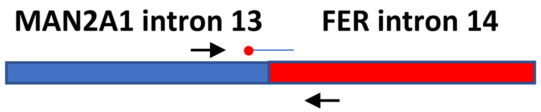

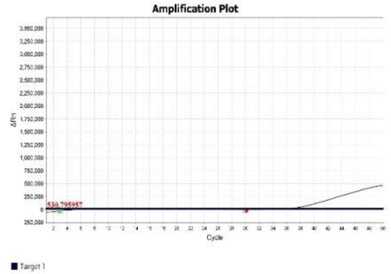

LnCAP

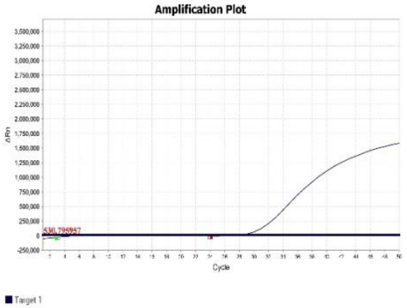

MDA-MB330

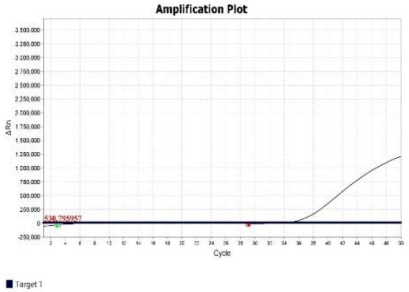

SNU387

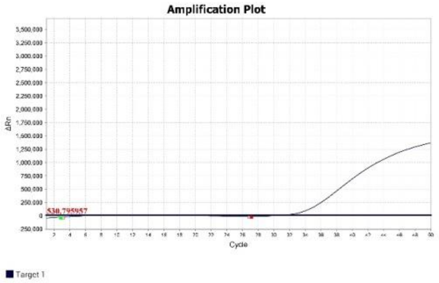

H522

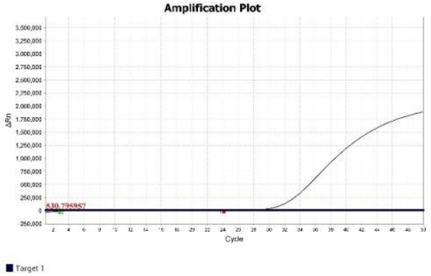

HCT15

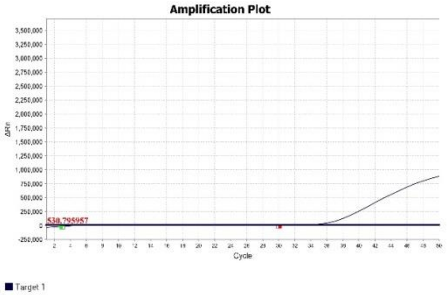

PC3

Supplemental figure 1C

MAN2A1-FER breakpoint Taqman qPCR experiment #1

MAN2A1-FER Breakpoint

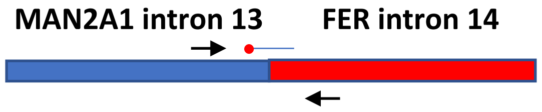

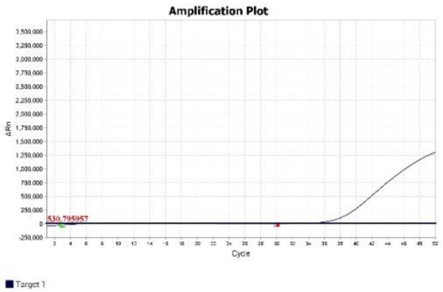

H1299

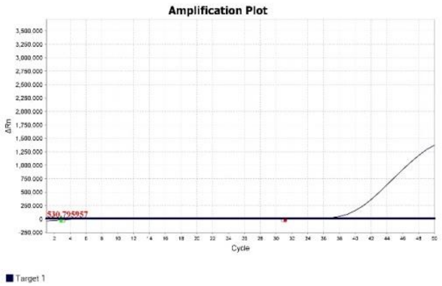

SNU182

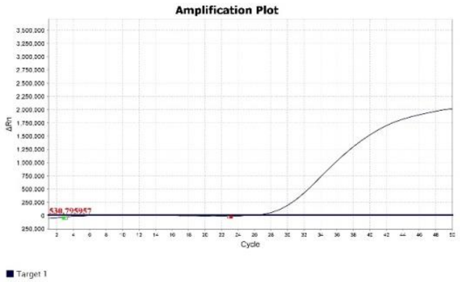

U118

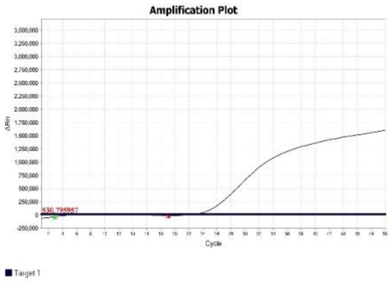

U138

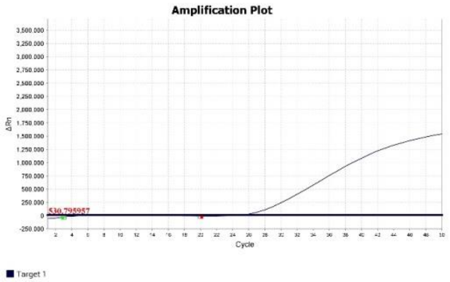

H2198

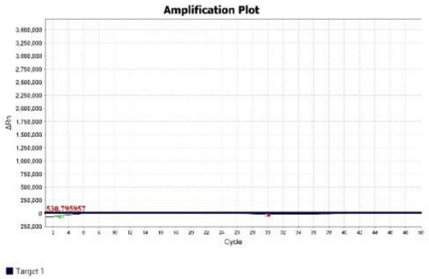

Hep3B

Supplemental figure 1C

MAN2A1-FER breakpoint Taqman qPCR experiment #1

MAN2A1-FER Breakpoint

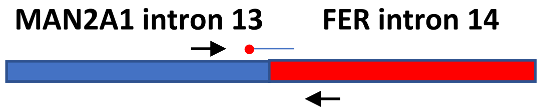

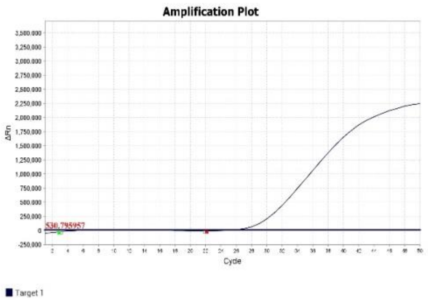

MCF7

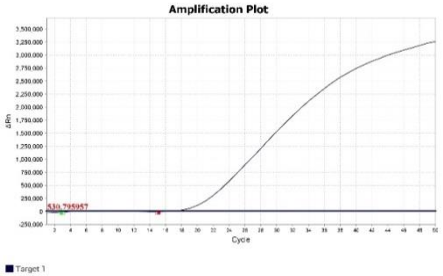

HuH7

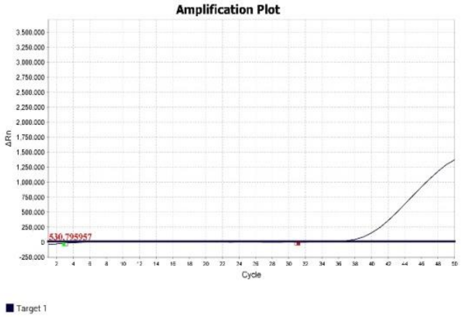

SNU449

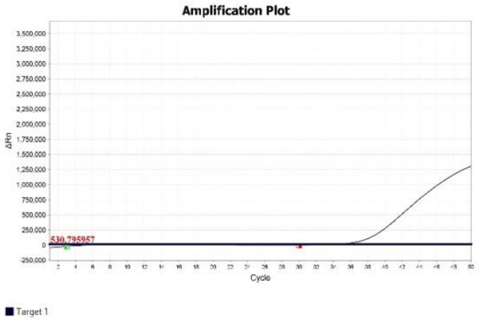

293

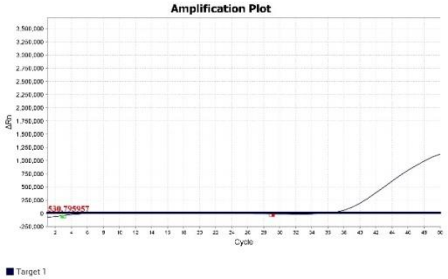

T98G

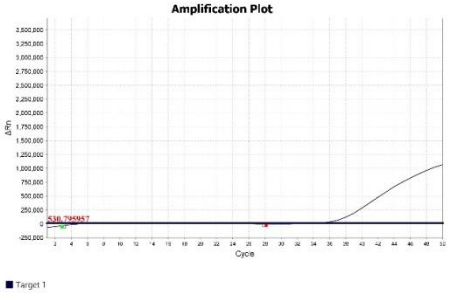

Ln229

Supplemental figure 1C

MAN2A1-FER breakpoint Taqman qPCR experiment #2

MAN2A1-FER Breakpoint

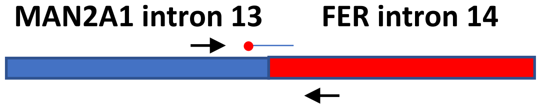

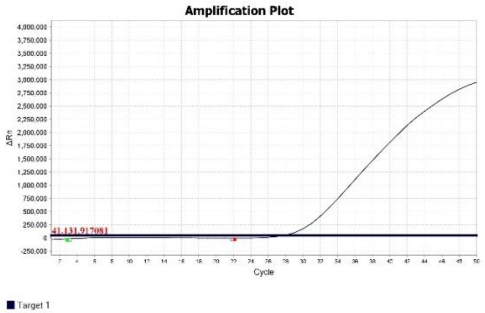

H358

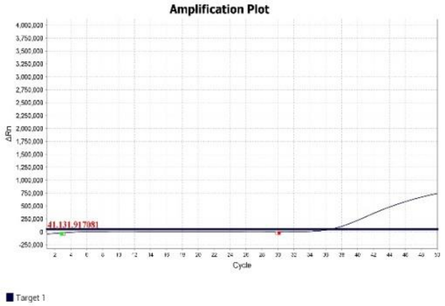

HCT8

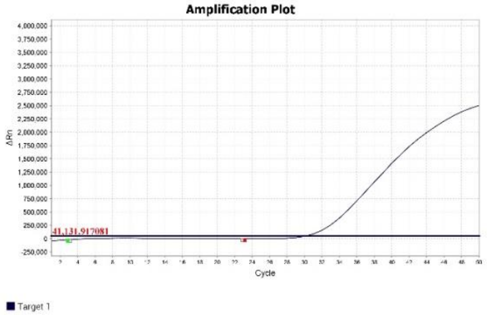

SNU475

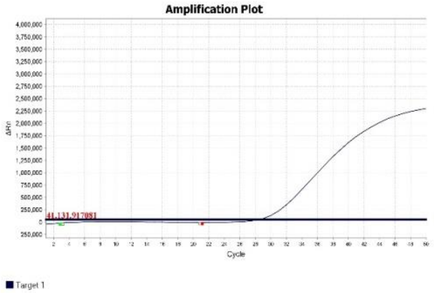

MB231

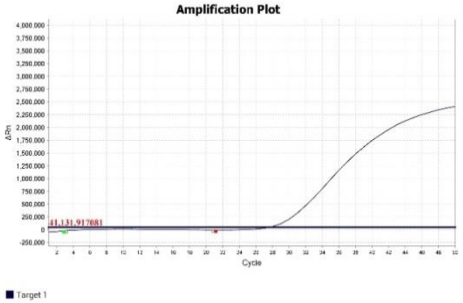

HepG2

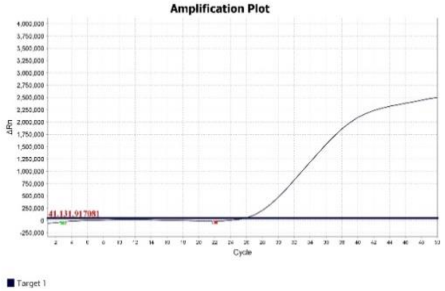

DU145

Supplemental figure 1C

MAN2A1-FER breakpoint Taqman qPCR experiment #2

MAN2A1-FER Breakpoint

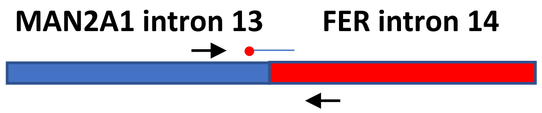

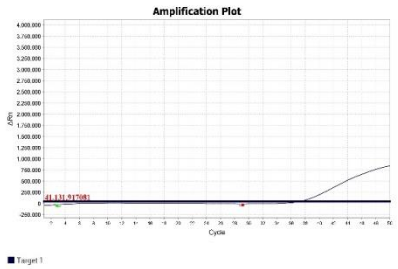

LnCAP

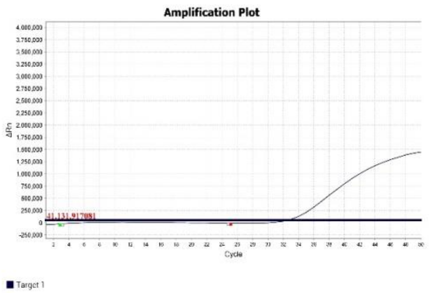

MDA-MB330

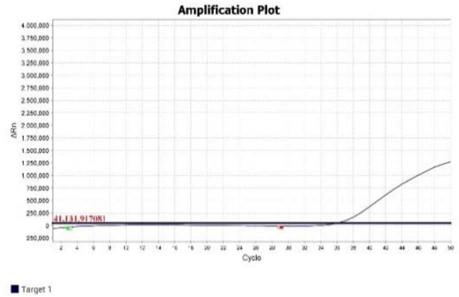

SNU387

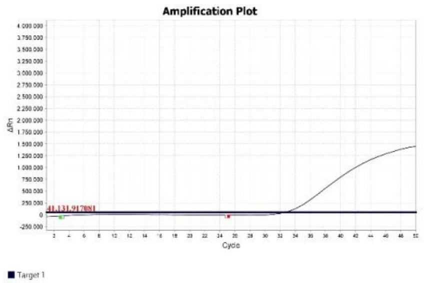

H522

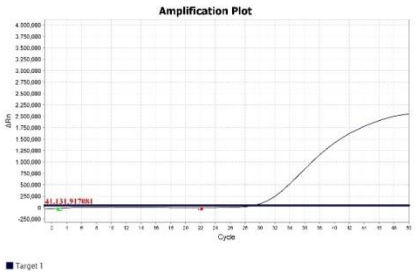

HCT15

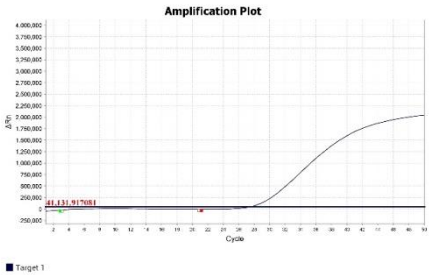

PC3

Supplemental figure 1C

MAN2A1-FER breakpoint Taqman qPCR experiment #2

MAN2A1-FER Breakpoint

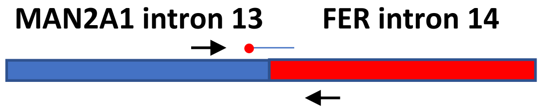

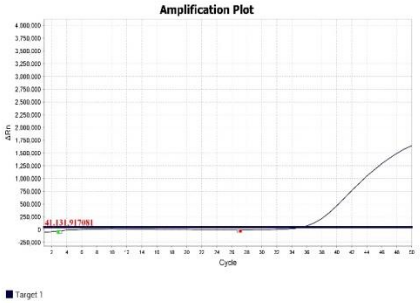

H1299

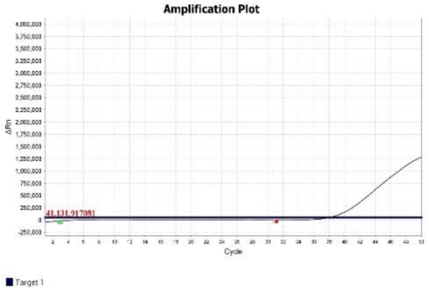

SNU182

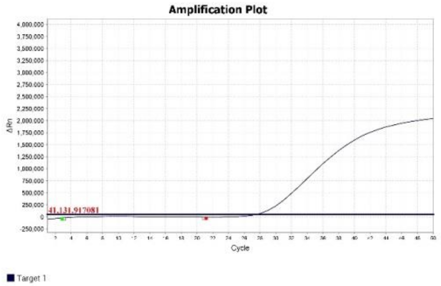

U118

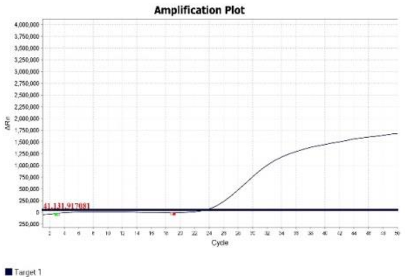

U138

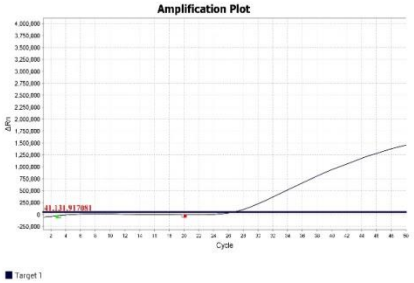

H2198

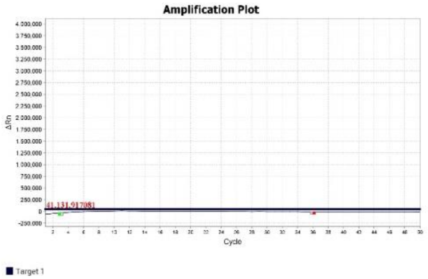

Hep3B

Supplemental figure 1C

MAN2A1-FER breakpoint Taqman qPCR experiment #2

MAN2A1-FER Breakpoint

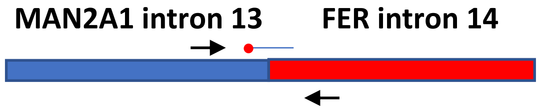

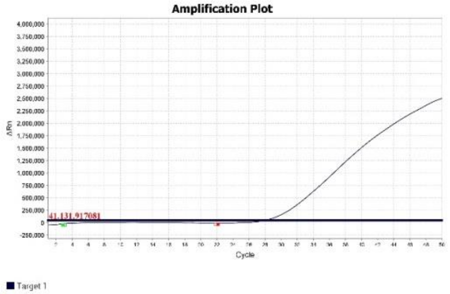

MCF7

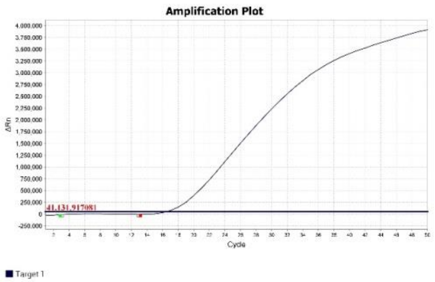

HuH7

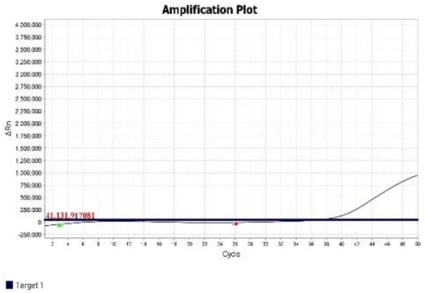

SNU449

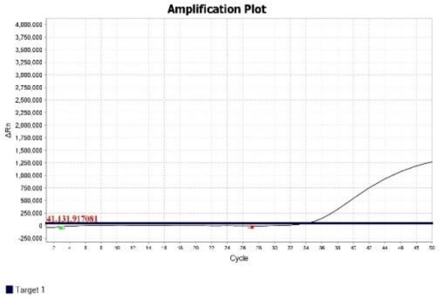

293

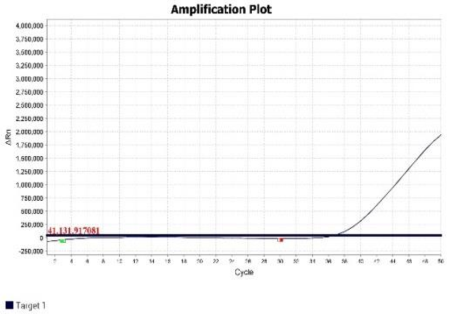

T98G

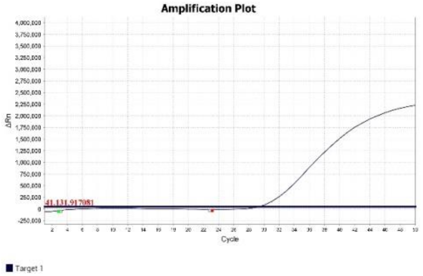

Ln229

Supplemental figure 1C

Beta-actin Taqman qPCR experiment #1

β-actin exon 6

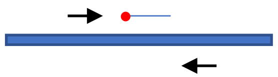

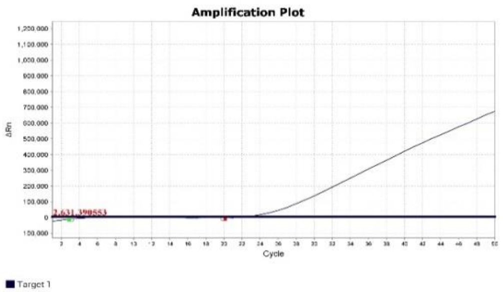

H358

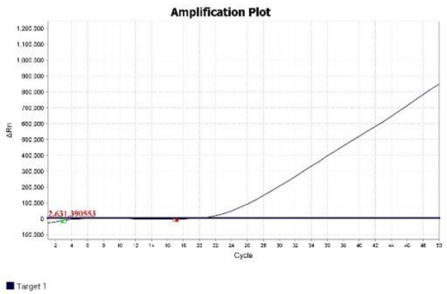

HCT8

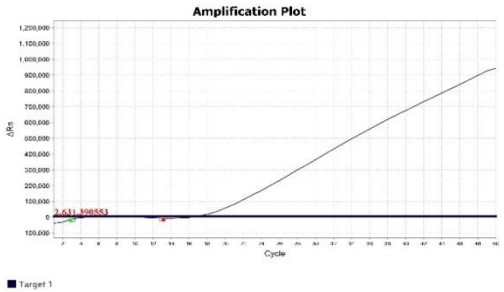

SNU475

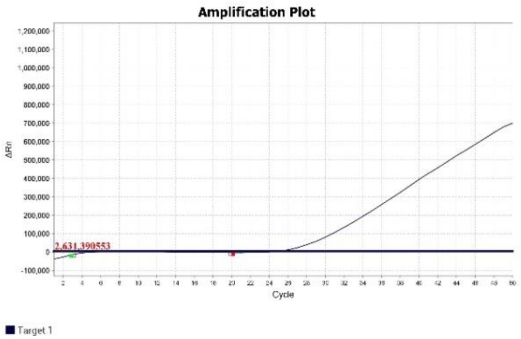

MB231

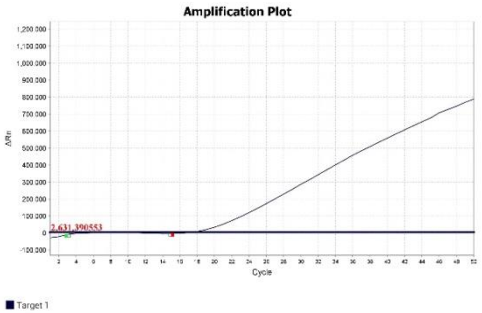

HepG2

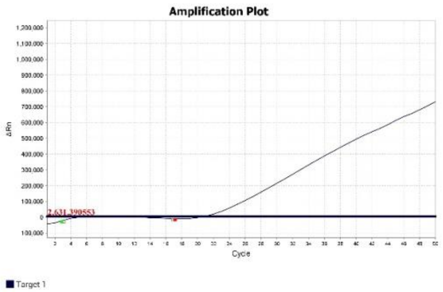

DU145

Supplemental figure 1C

Beta-actin Taqman qPCR experiment #1

β-actin exon 6

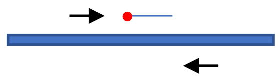

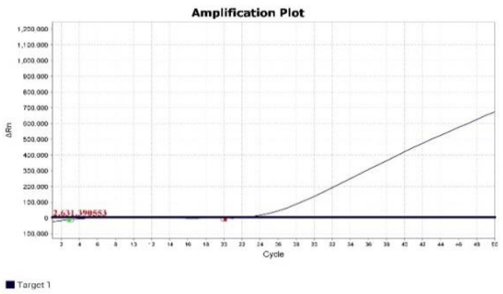

H358

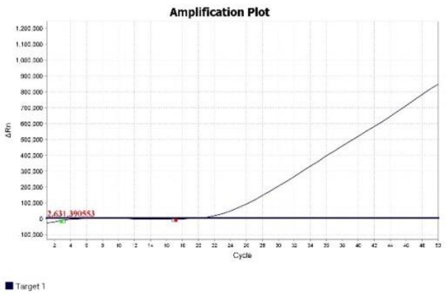

HCT8

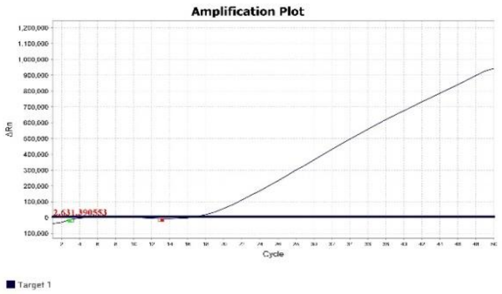

SNU475

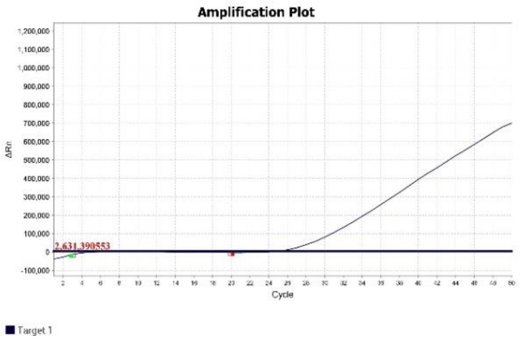

MB231

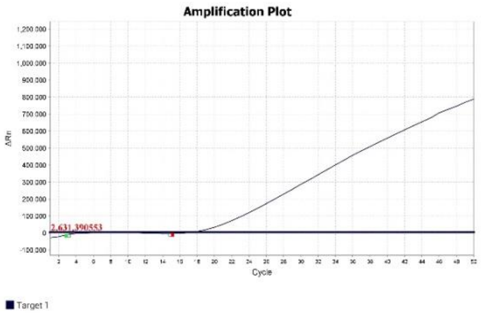

HepG2

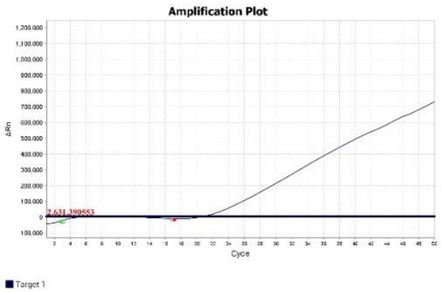

DU145

Supplemental figure 1C

Beta-actin Taqman qPCR experiment #1

β-actin exon 6

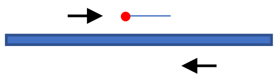

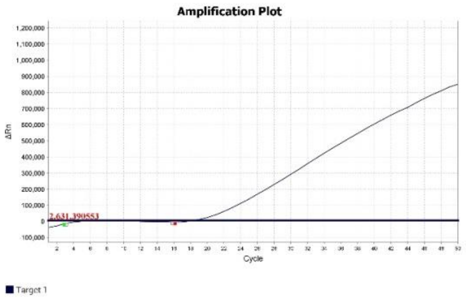

LnCAP

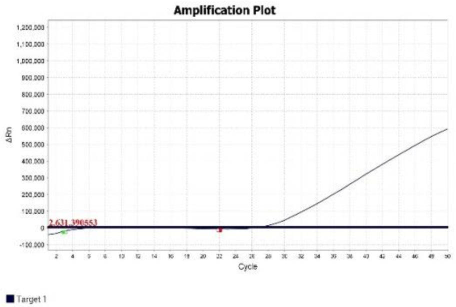

MDA-MB330

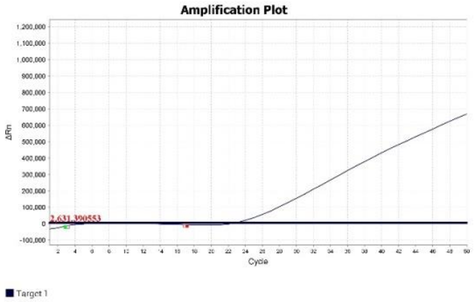

SNU387

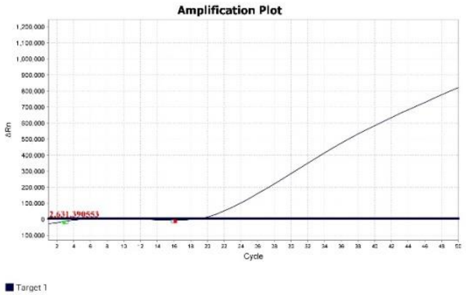

H522

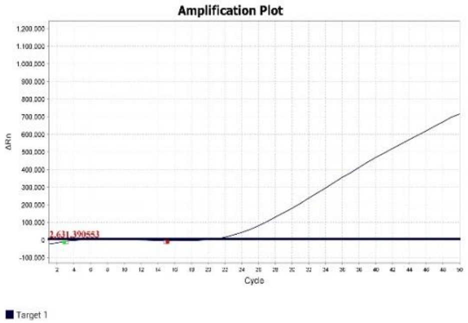

HCT15

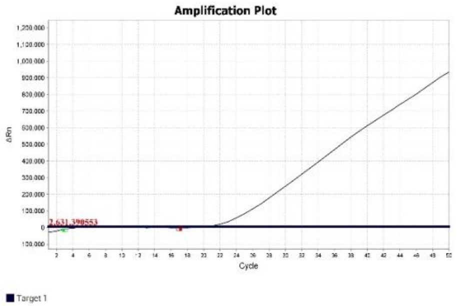

PC3

Supplemental figure 1C

Beta-actin Taqman qPCR experiment #1

β-actin exon 6

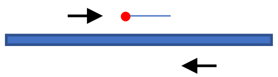

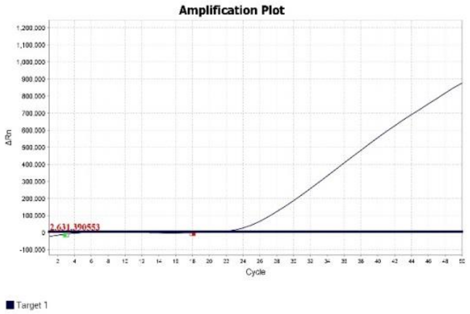

H1299

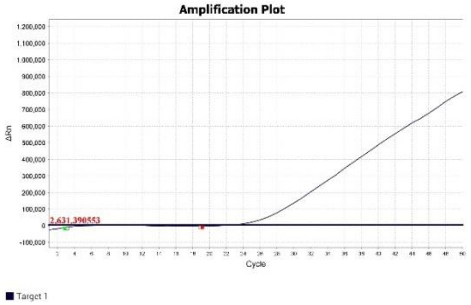

SNU182

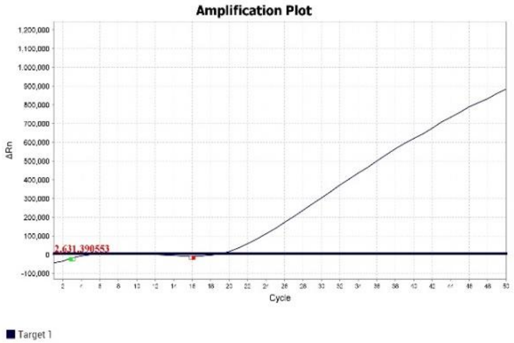

U118

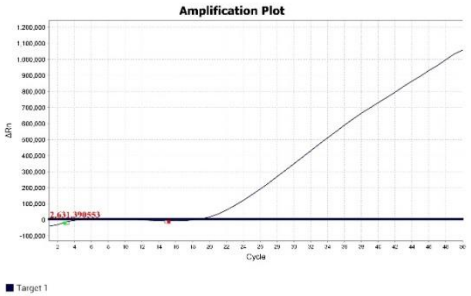

U138

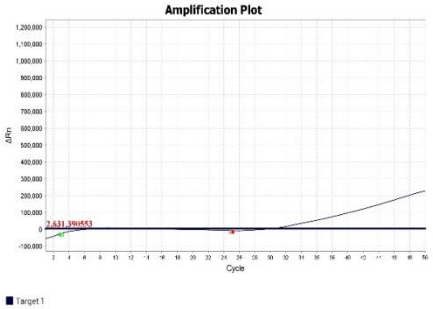

H2198

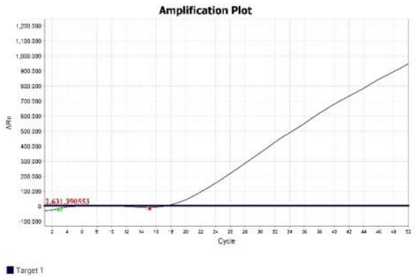

Hep3B

Supplemental figure 1C

Beta-actin Taqman qPCR experiment #1

β-actin exon 6

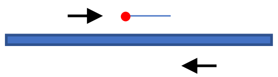

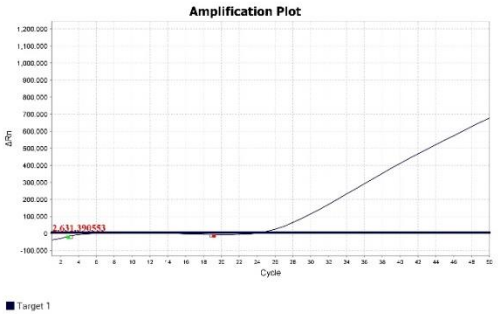

MCF7

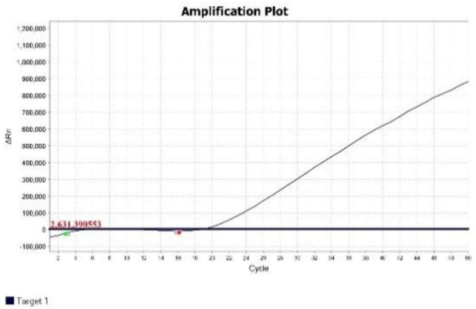

HuH7

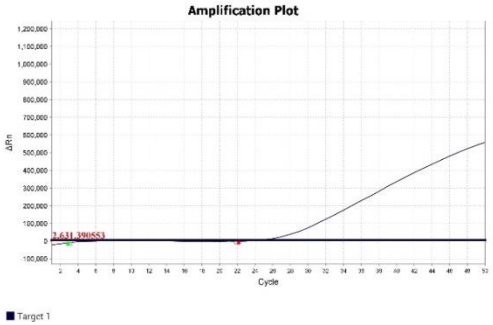

SNU449

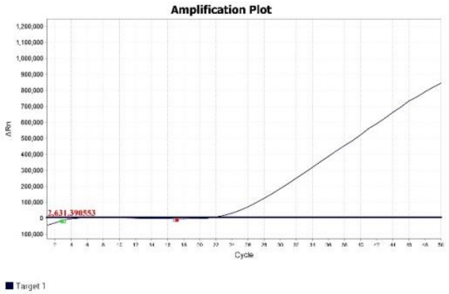

293

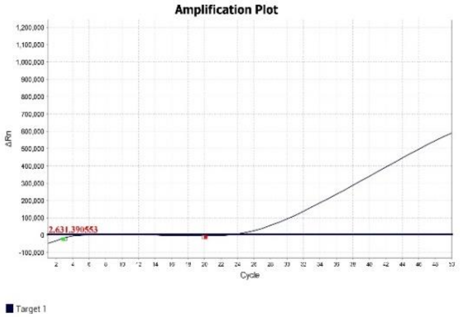

T98G

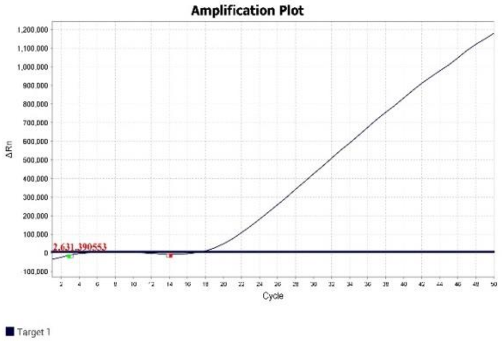

Ln229

Supplemental figure 1C

Beta-actin Taqman qPCR experiment #2

β-actin exon 6

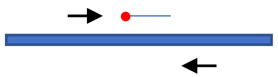

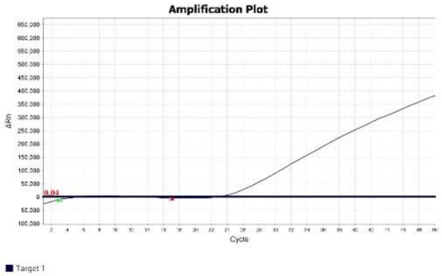

H358

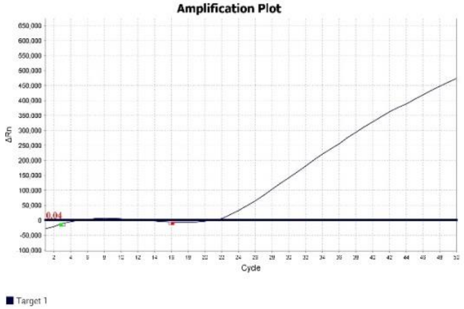

HCT8

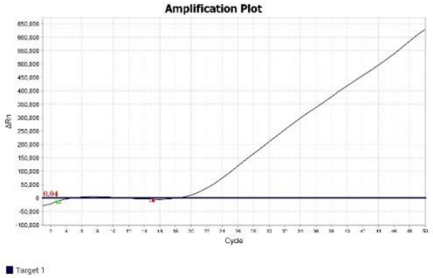

SNU475

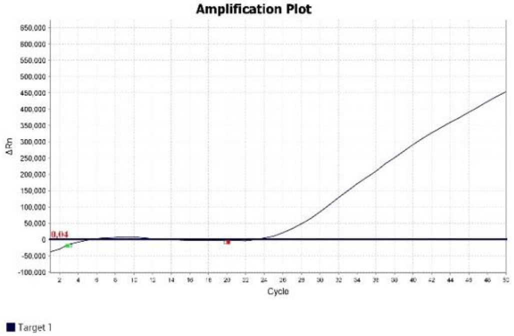

MB231

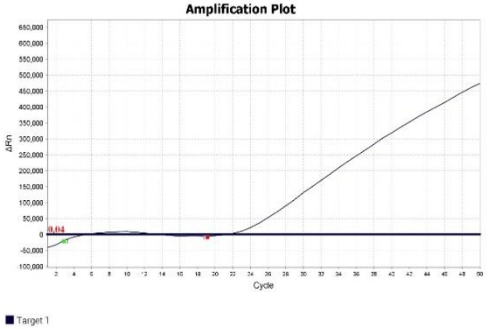

HepG2

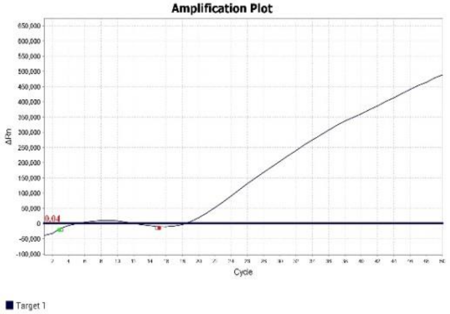

DU145

Supplemental figure 1C

Beta-actin Taqman qPCR experiment #2

β-actin exon 6

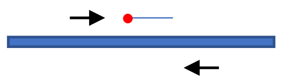

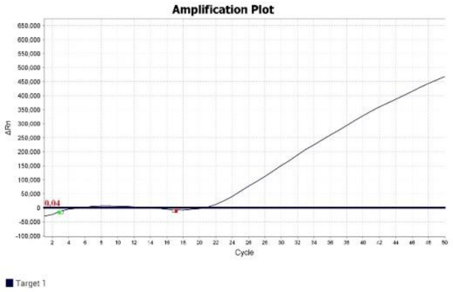

LnCAP

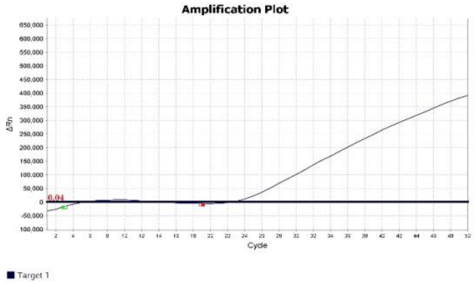

MDA-MB330

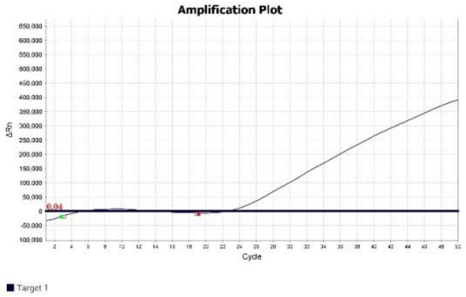

SNU387

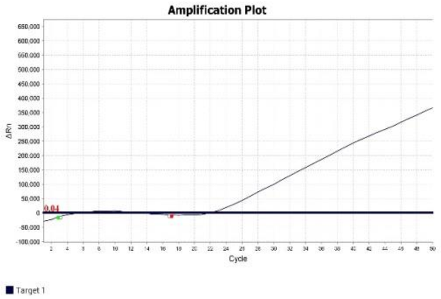

H522

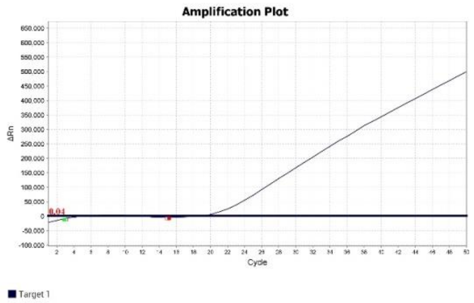

HCT15

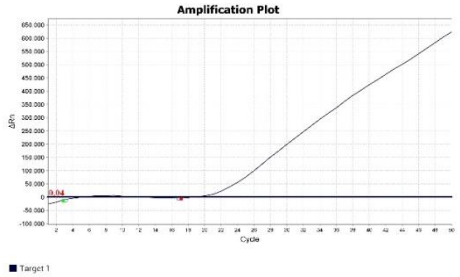

PC3

Supplemental figure 1C

Beta-actin Taqman qPCR experiment #2

β-actin exon 6

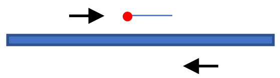

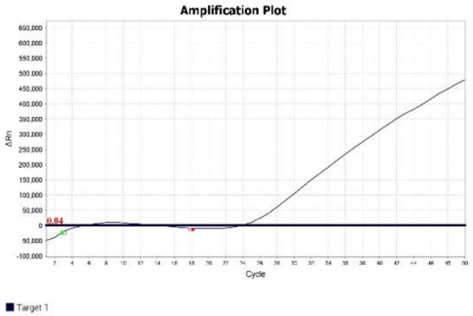

H1299

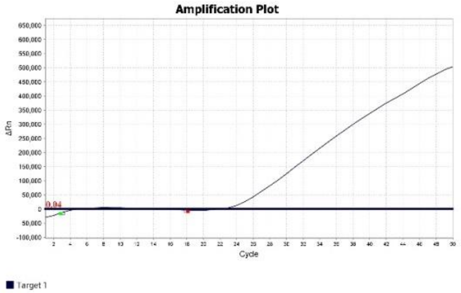

SNU182

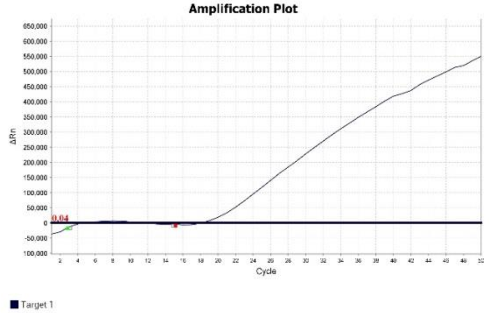

U118

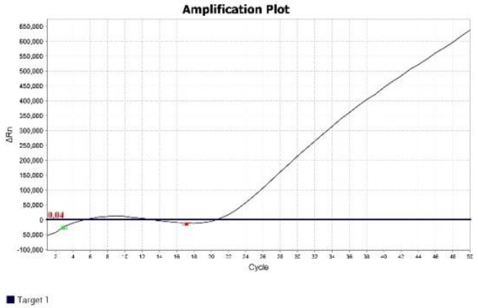

U138

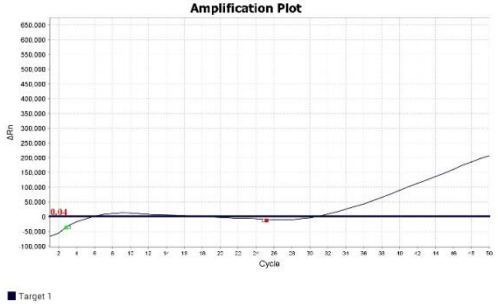

H2198

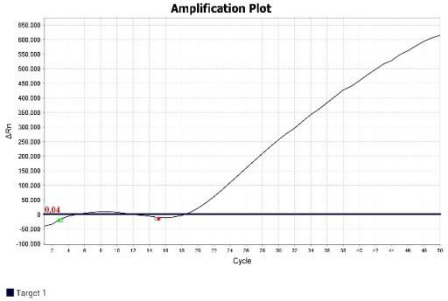

Hep3B

Supplemental figure 1C

Beta-actin Taqman qPCR experiment #2

β-actin exon 6

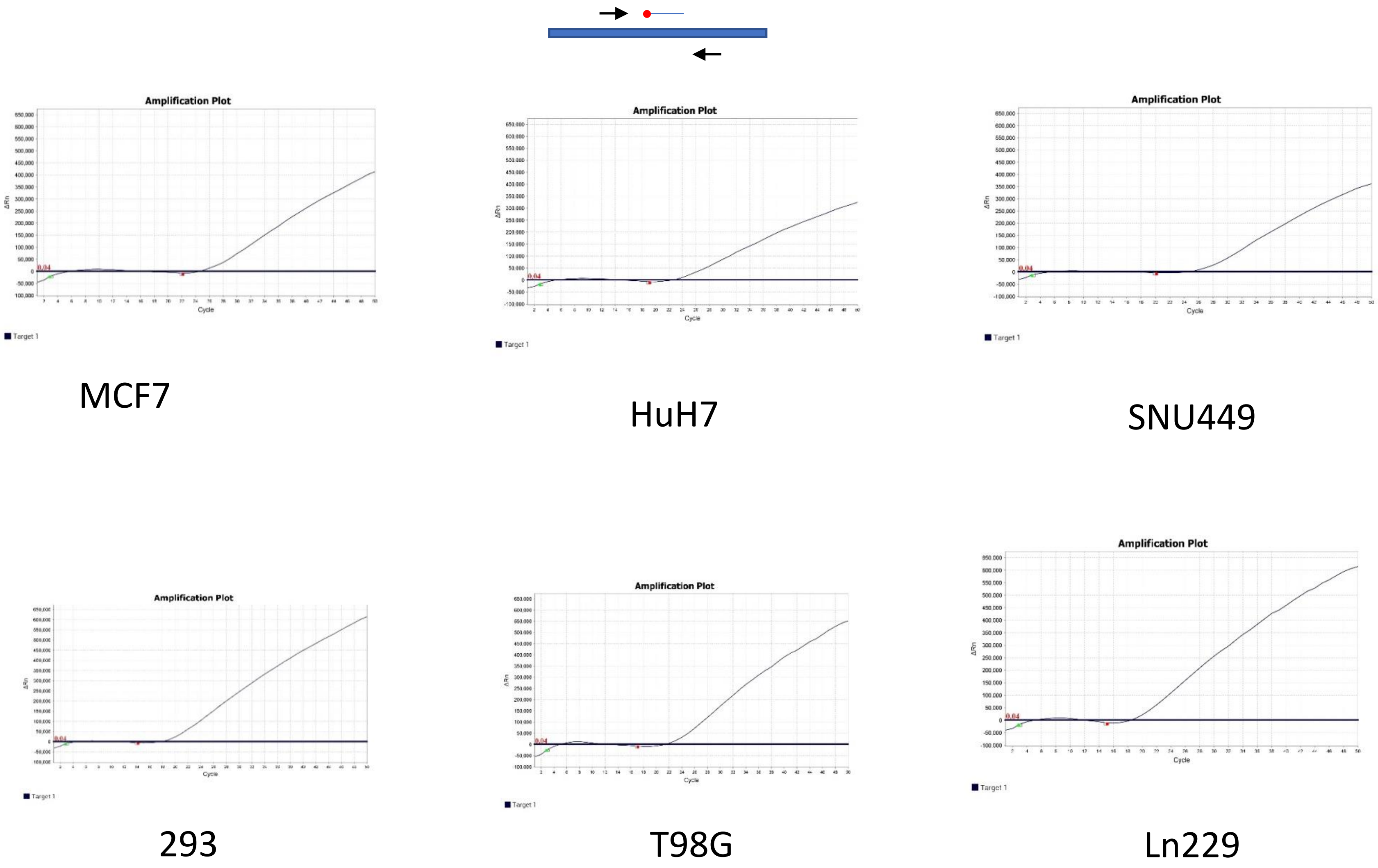

Supplemental figure 1D

(D) Images of Chromogram of Sanger's sequencing on the genome breakpoint juncture of MAN2A1-FER.

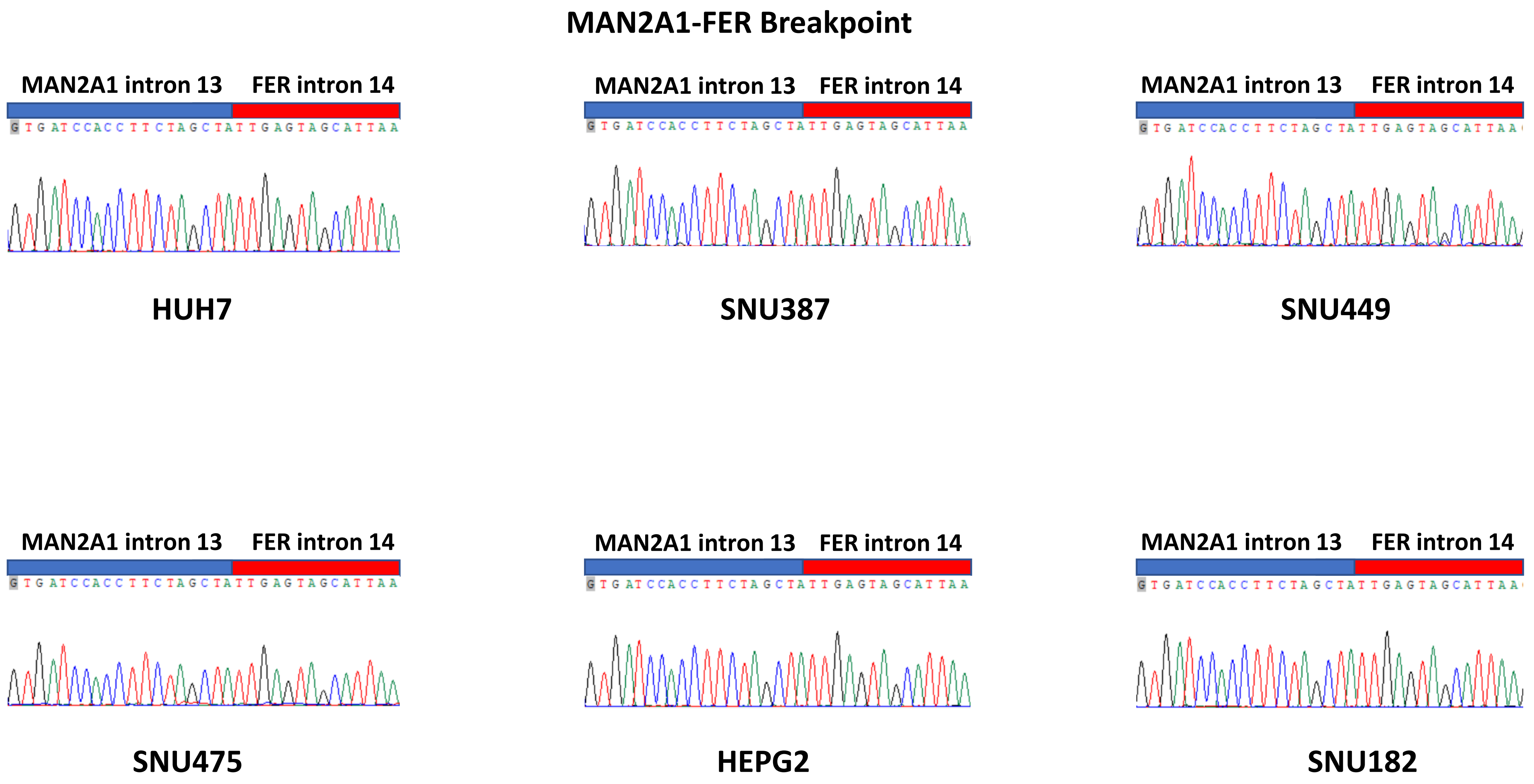

Supplemental figure 1D

MAN2A1-FER Breakpoint

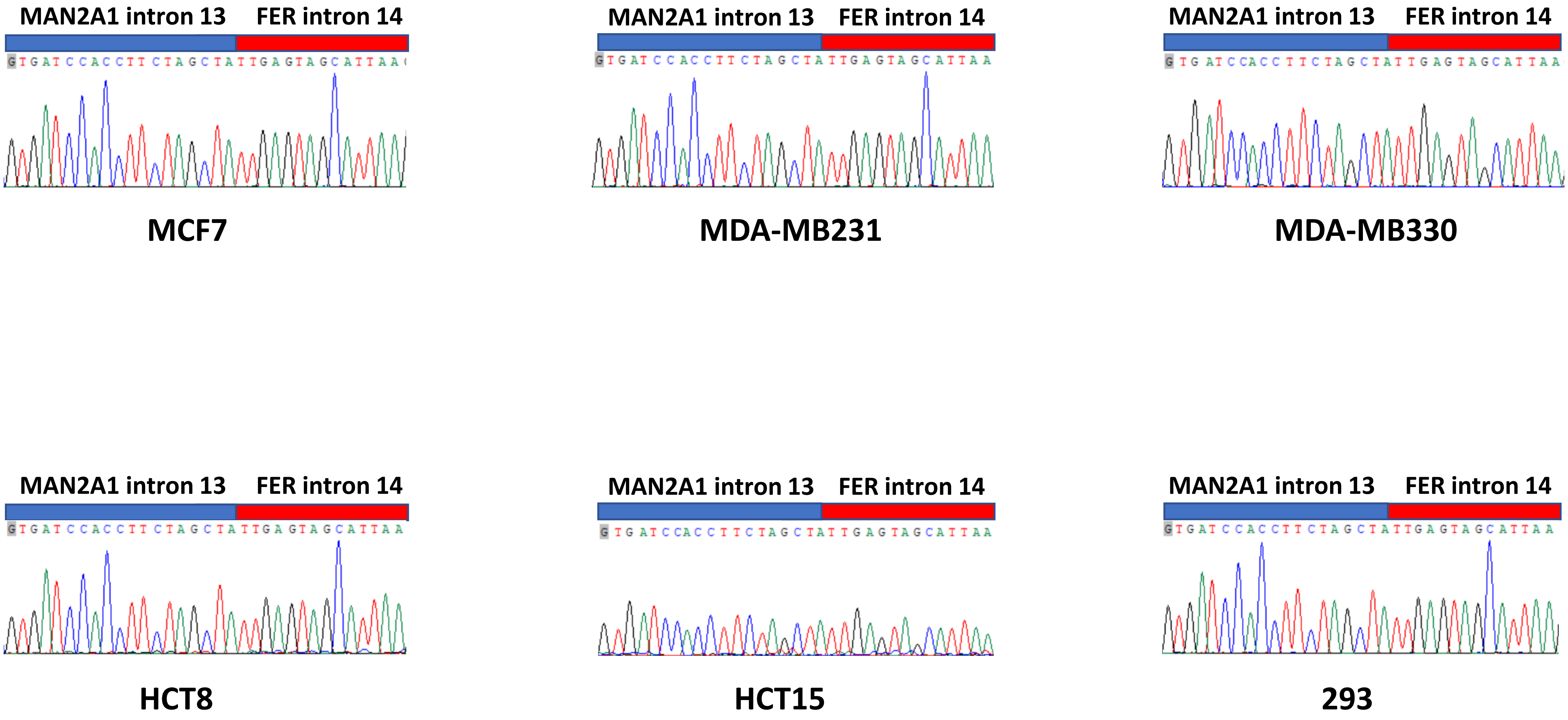

Supplemental figure 1D

MAN2A1-FER Breakpoint

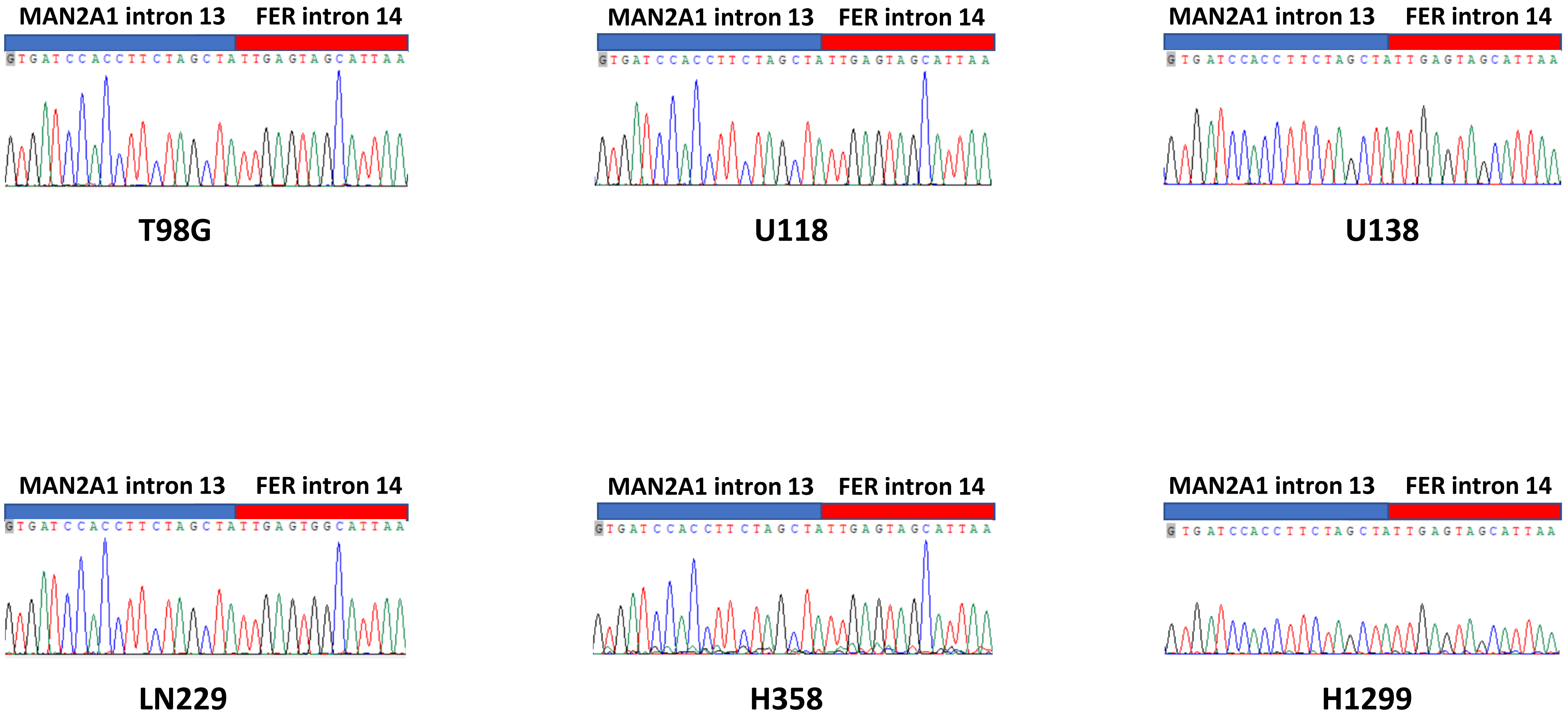

Supplemental figure 1D

MAN2A1-FER Breakpoint

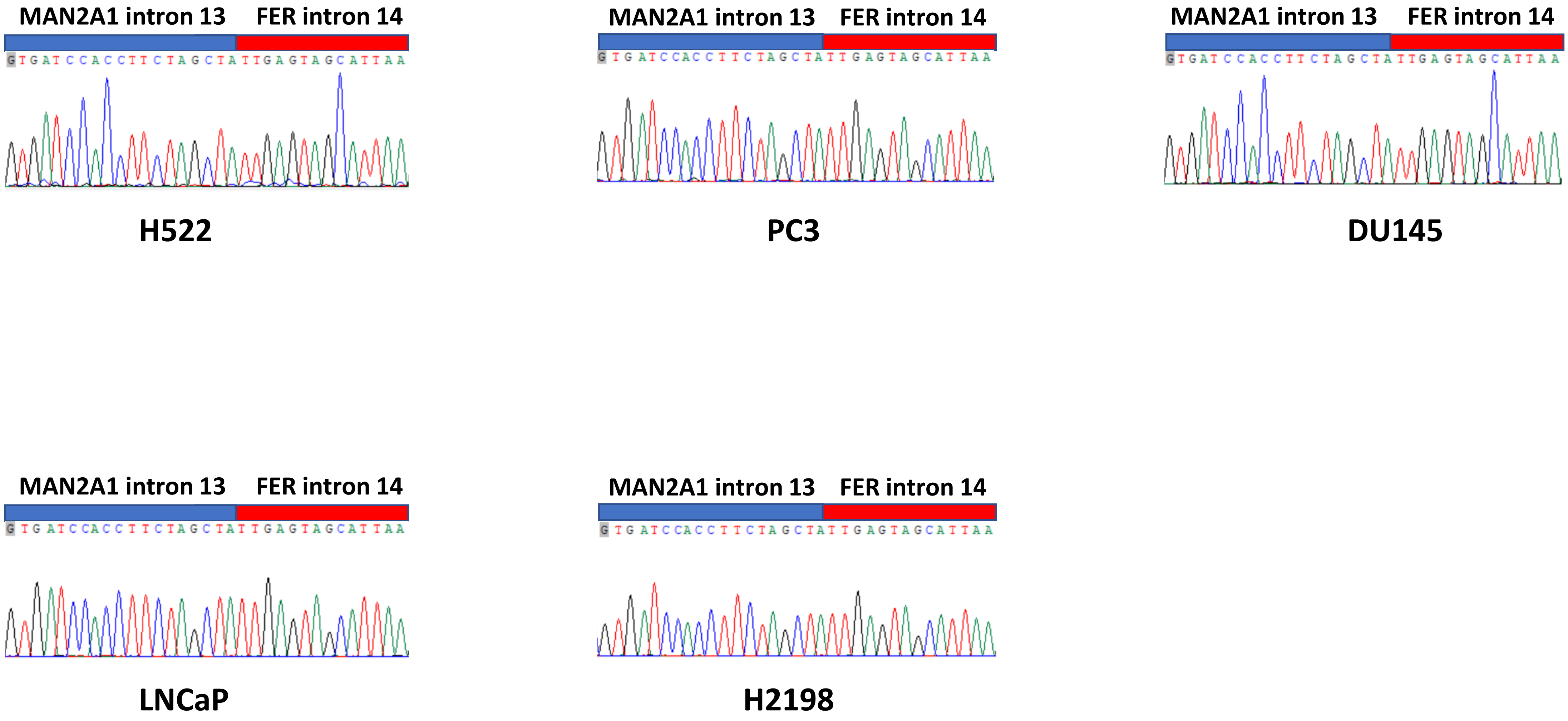

Supplemental figure 2. Frequency of MAN2A1-FER expression in colon and breast cancer. The total number of cases of each type of cancer is indicated.

Frequency of MAN2A1-FER expression in colon and breast cancer. The total number of cases of each type of cancer is indicated.

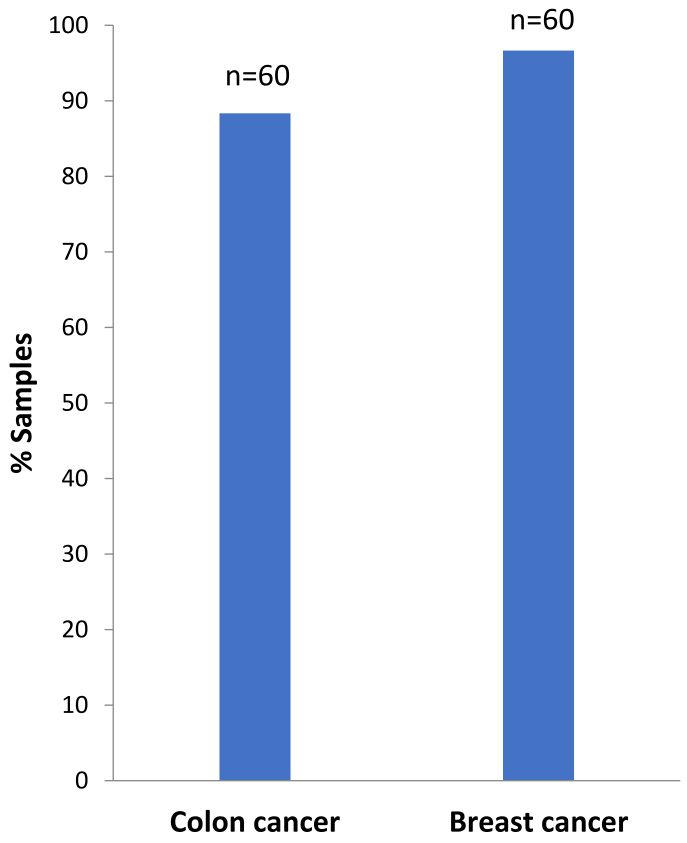

Supplemental figure 3. Co-immunoprecipitation of MAN2A1-FER-FLAG with EGFR, AXL, CDH2, MET and PDGFRA. Upper panel: The protein extract from HEP3B cells transfected with pCDNA4-MAN2A1-FER-FLAG were immunoprecipitated with IgG or anti-FLAG antibody and immunoblotted with anti-EGFR (lanes 1-3), anti-AXL (lanes 4-6), anti-CDH2 (lanes 7-9), anti-MET (lanes 10-12), or anti-PDGFRA (lanes 13-15). The protein extract without immunoprecipitation is the "Input" control. Lower panel: The protein extract from HEP3B cells transfected with pCDNA4-MAN2A1-FER-FLAG was immunoprecipitated with IgG or the indicated antibodies and immunoblotted with anti-FLAG antibody. The protein extract without immunoprecipitation is the "Input" control.

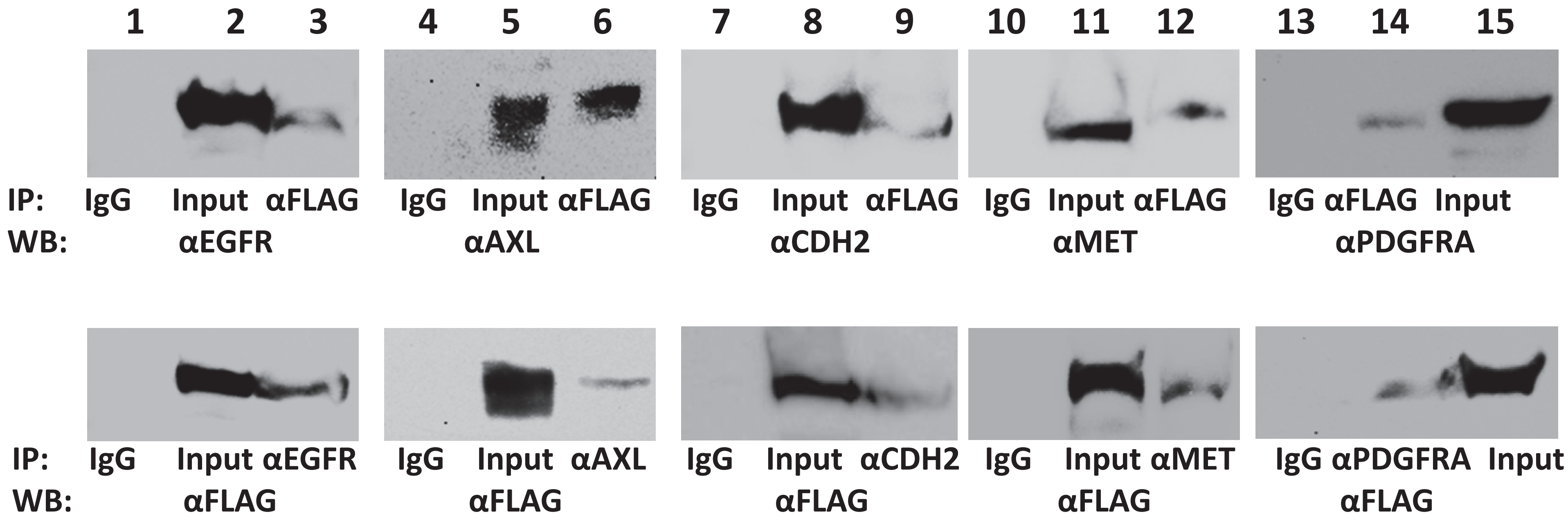

Supplemental figure 4. The impact of PDGFRA neutralizing antibody MA5-38592, PDGFR tyrosine kinase inhibitor AG-1295, and siPDGFRA on HUH7, HEP3B, and HUH7ko cells. (A) Immunoblotting of HUH7, HEP3B, and HUH7ko cells treated with mouse IgG, 2-3b-G8 antibody, MA5-38592 (anti-PDGFRA) antibody, DMSO medium (Mock), AG-1295, Universal negative siRNA control (Scr), and siPDGFRA, with the indicated antibodies. (B) The impact of non-specific IgG, 2-3b-G8 (G8), PDGFRA neutralizing antibody MA5-38592 (anti-PDGFRA), PDGFR tyrosine kinase inhibitor (AG-1295), Universal negative siRNA control (Scr), and siPDGFRA on cell cycles of HUH7, HEP3B, and HUH7ko cells. Triplicate experiments were performed. Standard deviations were shown. Student t-tests were performed. * denotes p<0.05; ** denotes p<0.01. (C) The impact of IgG, 2-3b-G8m (G8), PDGFRA neutralizing antibody MA5-38592 (anti-PDGFRA), PDGFR tyrosine kinase inhibitor (AG-1295), Universal negative siRNA control (Scr), and siPDGFRA on cell death of HUH7, HEP3B, and HUH7ko cells. Triplicate experiments were performed. Standard deviations were shown. Student t-tests were performed. ** denotes p<0.01.

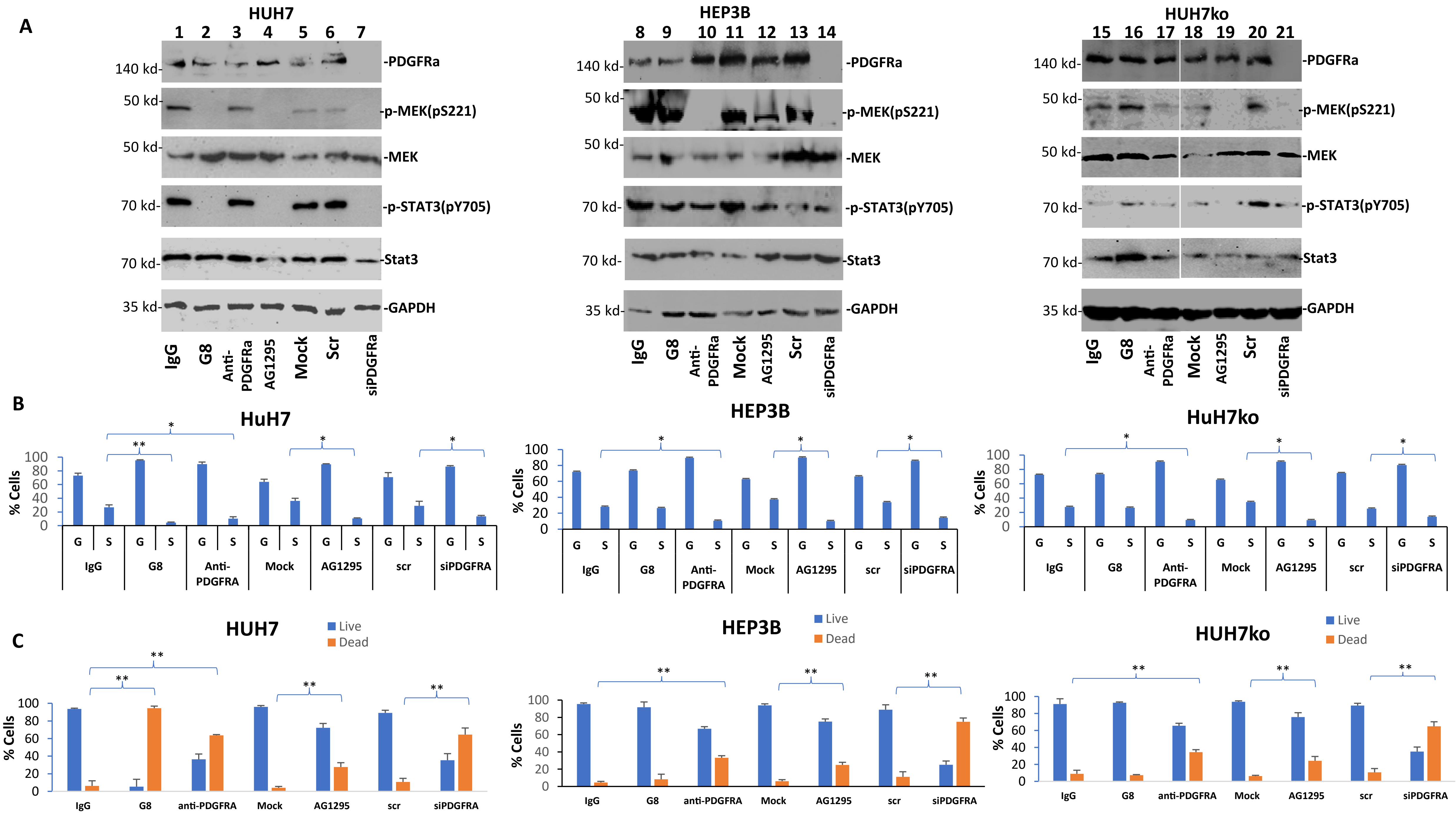

Supplement: SUPPLEMENTARY MATERIAL [file hc9-8-e0511-s002.pdf]
